# Neutral inverse-sandwich rare-earth metal complexes of the benzene tetraanion†

**DOI:** 10.1039/d4sc02491e

**Published:** 2024-05-13

**Authors:** Yi Wang, Yurou Zhang, Jiefeng Liang, Bowen Tan, Chong Deng, Wenliang Huang

**Affiliations:** a Beijing National Laboratory for Molecular Sciences, College of Chemistry and Molecular Engineering, Peking University Beijing 100871 P. R. China wlhuang@pku.edu.cn

## Abstract

Rare-earth metal complexes of the parent benzene tetraanion and neutral inverse-sandwich rare-earth metal arene complexes have remained elusive. Here, we report the first neutral inverse-sandwich rare-earth metal complexes of the parent benzene tetraanion supported by a monoanionic β-diketiminate (BDI) ligand. Reduction of the trivalent rare-earth metal diiodide precursors (BDI)MI_2_(THF) (BDI = HC(C(Me)N[C_6_H_3_-(3-pentyl)_2_-2,6])_2_; M = Y, 1-Y; M = Sm, 1-Sm) in benzene or *para*-xylene by potassium graphite yielded the neutral inverse-sandwich rare-earth metal arene complexes [(BDI)M(THF)_*n*_]_2_(μ-η^6^,η^6^-arene) (M = Y, Sm; arene = benzene, 2-M; arene = *para*-xylene, 3-M). Single crystal X-ray diffraction, spectroscopic and magnetic characterization studies, together with density functional theory (DFT) calculations confirm that these neutral rare-earth metal arene complexes possess an [M^3+^–(arene)^4−^–M^3+^] electronic structure with strong metal–arene δ interactions. The arene exchange reactivity shows that 2-Sm has higher stability than 3-Sm. Furthermore, 2-Sm can behave as a four-electron reductant to reduce unsaturated organic substrates. Particularly, while the reaction of 2-Sm with 1,3,5,7-cyclooctatetraene (COT) yielded (BDI)Sm(η^8^-COT) (4-Sm), 2-Sm reacted with 1,4-diphenylbutadiyne to afford (BDI)Sm(η^4^-C_4_Ph_2_) (5-Sm), the first rare-earth metallacyclopentatriene complex.

## Abstract 2

Neutral inverse-sandwich rare-earth metal complexes of the parent benzene tetraanion with an [M^3+^–(C_6_H_6_)^4−^–M^3+^] core electronic structure were synthesized and characterized. They behave as a four-electron reductant to reduce unsaturated substrates.

## Graphical abstract

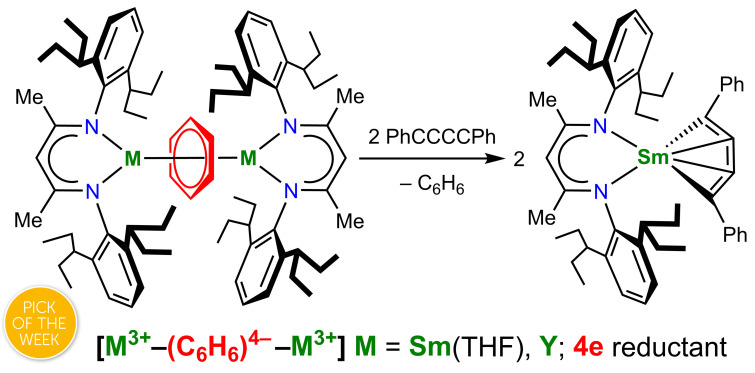

## Introduction

**Fig. 1:**
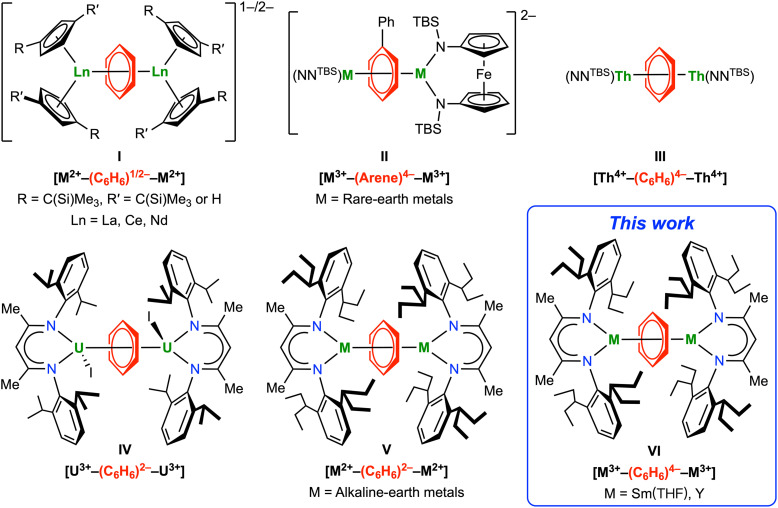
Inverse-sandwich metal complexes of bridging arene anions relevant to this work.

Since Fischer's seminal work on bis(benzene)chromium,^1^ metal arene complexes have grown into one of the most important classes of organometallic compounds.^2,3^ While the bound arenes are usually considered neutral in transition metal arene complexes,^4–6^ the more electropositive rare-earth metals tend to form complexes with reduced arene anions.^7–10^ Compared to readily reducible polyarenes, such as naphthalene and anthracene, the parent benzene is much more difficult to reduce (−3.42 V *vs.* SCE),^11^ which results in fewer rare-earth metal reduced benzene complexes compared to other arenes.^7^ In 1996, Lappert and co-workers reported the first metal complexes containing a benzene 1,4-dianion, [K(18-crown-6)][Cp′′_2_Ln(C_6_H_6_)] (Cp′′ = η^5^-C_5_H_3_(SiMe_3_)_2_-1,3, Ln = La, Ce).^12^ Later, they reported the first inverse-sandwich rare-earth metal benzene complex [K(18-crown-6)(η^2^-C_6_H_6_)_2_][(Cp^tt^_2_La)_2_(μ-η^6^,η^6^-C_6_H_6_)] (Cp^tt^ = η^5^-C_5_H_3_(CMe_3_)_2_-1,3), which is formulated as a benzene radical monoanion bridging two La(ii) ions, *i.e.* [La^2+^–(C_6_H_6_)^−^–La^2+^] (I, Fig. 1).^13^ More recently, Evans and Roesky reported synthesis and structural characterization of several closely relevant inverse-sandwich rare-earth metal benzene complexes [K(18-crown-6)]_*n*_[(X_2_Ln)_2_(μ-η^6^,η^6^-C_6_H_6_)] (*n* = 1 or 2; X = Cp′′ or Cp′, Cp′ = η^5^-C_5_H_4_(SiMe_3_); Ln = La, Ce, Nd), which contain a benzene monoanion or dianion (I, Fig. 1).^14–17^ In addition, Mazzanti and co-workers reported quadruple-decker cerium and triple-decker samarium toluene-bridged complexes, both containing toluene dianion(s).^18,19^ While rare-earth metal complexes of the parent benzene tetraanion remain elusive, Diaconescu and Huang reported a series of rare-earth metal biphenyl complexes [K(solvent)]_2_[[(NN^TBS^)M]_2_(μ-η^6^,η^6^-C_6_H_5_Ph)] (NN^TBS^ = fc(NSi^*t*^BuMe_2_)_2_, fc = 1,1′-ferrocenediyl; M = Sc, Y, La, Lu, Gd, Dy, Er, Sm), featuring a μ-η^6^,η^6^-bound biphenyl tetraanion with most negative charges localized at the bound ring (II, Fig. 1).^20–22^ Notably, all these inverse-sandwich rare-earth metal complexes of reduced benzene or substituted benzenes are ion-pair complexes with negative charges balanced by alkali metal counter cations. This is probably because the large ionic radii of rare-earth ions require two or more anionic ligands, such as Cp derivatives, amides or siloxides, for steric protection. On the other hand, inverse-sandwich uranium arene complexes are more common and the majority of them are neutral complexes without a counter cation.^23–41^ While it is challenging to unambiguously assign the oxidation states of uranium and the bound arene in these complexes due to covalent δ bonding interactions,^25^ in most cases the bridging arenes are considered as dianionic.^35,40^ In 2020, our group reported the first unambiguous parent benzene tetraanion in a neutral inverse-sandwich thorium benzene complex [(NN^TBS^)Th]_2_(μ-η^6^,η^6^-C_6_H_6_) (III, Fig. 1).^42^ Later, Mazzanti and co-workers reported an inverse-sandwich thorium naphthalene complex [K(OSi(O^*t*^Bu)_3_)_3_Th]_2_(μ-η^6^,η^6^-C_10_H_8_), also featuring a naphthalene tetraanion.^43^ The DFT calculations on a series of inverse-sandwich f-block metal arene complexes [(NN^TBS^)M]_2_(μ-η^6^,η^6^-arene) (M = rare-earth metals, Th, U) show that the covalency of δ bonding interactions follows the trend: rare-earth metals < Th < U.^22,42^

The lack of rare-earth metal complexes of the parent benzene tetraanion and neutral inverse-sandwich rare-earth metal arene complexes prompted us to pursue the synthesis of neutral inverse-sandwich rare-earth metal complexes of the parent benzene tetraanion. We rationalized that the anionic nature of previously reported inverse-sandwich rare-earth metal arene complexes may weaken the rare-earth metal–arene δ interaction by electrostatic repulsion between anionic ancillary ligands and the anionic arene ligand. Therefore, the use of a sterically demanding, monoanionic ligand may stabilize the target product, which should be a neutral complex with a formula of (LM)_2_(μ-η^6^,η^6^-C_6_H_6_) (L = a monoanionic ligand). As a widely used monoanionic ligand in coordination chemistry across the periodic table,^44–47^ β-diketiminate ligands have shown some advantages in stabilizing inverse-sandwich metal arene complexes. In 2013, Liddle and co-workers reported the synthesis of an inverse-sandwich uranium benzene complex [(BDI′)UI]_2_(μ-η^6^,η^6^-C_6_H_6_) (BDI′ = HC(C(Me)N[C_6_H_3_-(2-propyl)_2_-2,6])_2_) supported by the BDI′ ligand, albeit in low yield (IV, Fig. 1).^36^ More recently, Anker and co-workers showed that the BDI′ ligand can be employed to stabilize dinuclear ytterbium naphthalene and anthracene complexes [(BDI′)Yb(Et_2_O)]_2_(μ-η^6^,η^6′^-arene) (arene = C_10_H_8_ or C_14_H_10_).^48^ In addition, Harder and co-workers showed that the bulky BDI ligand can be employed to stabilize inverse-sandwich alkaline-earth metal benzene complexes [(BDI)M]_2_(μ-η^6^,η^6^-C_6_H_6_) (BDI = HC(C(Me)N[C_6_H_3_-(3-pentyl)_2_-2,6])_2_; M = Mg, Ca, Sr), featuring a bridging benzene dianion (V, Fig. 1).^49–51^ Encouraged by these reports as well as the recent success of bulky BDI ligands in stabilizing low-valent main group metals,^47,49–59^ we considered that the bulky BDI ligand may be suitable for supporting neutral rare-earth metal benzene complexes. Here, we report the synthesis and characterization of neutral inverse-sandwich rare-earth metal benzene and *para*-xylene (*p*-xylene) complexes [(BDI)M(THF)_*n*_]_2_(μ-η^6^,η^6^-arene) (BDI = HC(C(Me)N[C_6_H_3_-(3-pentyl)_2_-2,6])_2_; M = Y, Sm; arene = benzene, 2-M; arene = *p*-xylene, 3-M; VI, Fig. 1) supported by the bulky BDI ligand. Combined experimental and calculation results agree with the formulation of [M^3+^–(arene)^4−^–M^3+^] for 2-M and 3-M, *i.e.* a benzene tetraanion bridging two trivalent rare-earth metal centres. Moreover, reactivity studies show that these rare-earth metal arene complexes can serve as four-electron reductants to reduce unsaturated organic molecules.

## Results and discussion

**Scheme 1:**
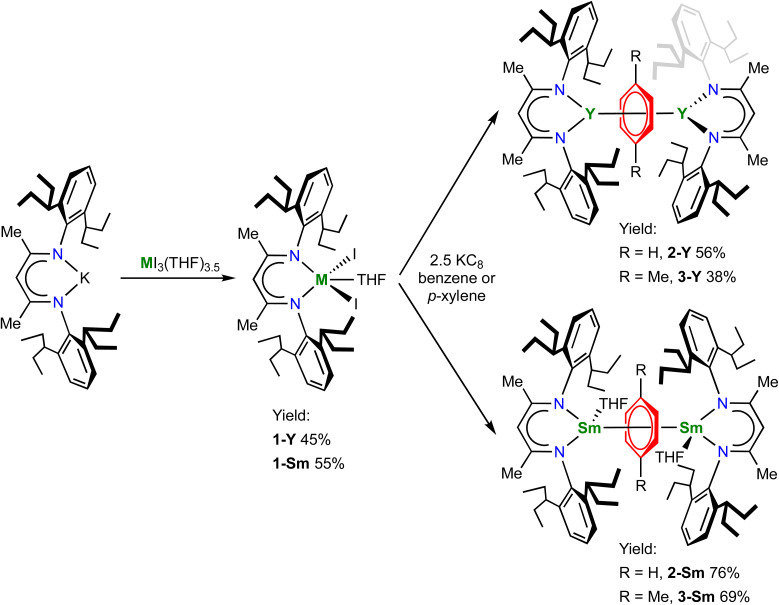
Synthesis of 1-M, 2-M and 3-M.

The pro-ligand (BDI)H and the potassium salt (BDI)K were prepared according to literature procedures.^49,50^ We choose samarium and yttrium as representative light and heavy rare-earth metals, because they have the intermediate ionic radii among their leagues, respectively. In addition, since samarium has a readily accessible +2 oxidation state and benzene is more difficult to reduce than biphenyl, it will be of interest to compare the electronic structures of the target samarium benzene complexes with the samarium biphenyl complexes recently reported by our group.^22^ The salt metathesis reaction between (BDI)K and YI_3_(THF)_3.5_ or SmI_3_(THF)_3.5_ gave the trivalent metal precursors (BDI)YI_2_(THF) (1-Y) and (BDI)SmI_2_(THF) (1-Sm) in moderate yields (Scheme 1). The ^1^H NMR spectrum of 1-Y in C_6_D_6_ shows the characteristic C*H* peak of the BDI ligand backbone at 5.17 ppm, which shifted downfield to 7.86 ppm in 1-Sm probably due to the paramagnetism of the Sm^3+^ ion. X-ray crystallography confirmed their monomeric structures (Fig. S14 and S15†). 1-Y and 1-Sm are isostructural and both adopt a distorted trigonal bipyramidal geometry. The oxygen atom of THF and one of the nitrogen atoms occupy the apical positions (O–Y–N: 169.8(2)°; O–Sm–N: 166.5(2)°), and the sum of angles of three equatorial ligands is close to 360° (1-Y: 359.6°; 1-Sm: 359.5°). The average Y–N and Y–I distances in 1-Y are 2.272(5) and 2.935(1) Å, respectively. The average Sm–N (2.311(4) Å) and Sm–I (3.003(1) Å) distances in 1-Sm are slightly longer than those in 1-Y, in line with the ionic radii difference (six-coordinate Y^3+^ 0.90 Å, Sm^3+^ 0.96 Å).^60^

### Synthesis and structural characterization

With 1-M in hand, we then attempted the synthesis of rare-earth metal benzene complexes under reducing conditions. Reduction of 1-M with 2.5 equiv. of potassium graphite (KC_8_) in benzene at room temperature led to a black suspension within several hours, indicating the full consumption of KC_8_. After work-up, the target product [(BDI)M(THF)_*n*_]_2_(μ-η^6^,η^6^-C_6_H_6_) (2-Y, M = Y, *n* = 0; 2-Sm, M = Sm, *n* = 1) was isolated as a black solid in 56% and 76% yields for yttrium and samarium, respectively (Scheme 1). In comparison, the reduction in *p*-xylene was more sluggish and took several days to complete, which may be attributed to the low solubility of the trivalent precursors 1-M in the less polar solvent *p*-xylene. Despite the slow kinetics, the corresponding *p*-xylene analogues [(BDI)M(THF)_*n*_]_2_(μ-η^6^,η^6^-C_8_H_10_) (3-Y, M = Y, *n* = 0; 3-Sm, M = Sm, *n* = 1) could be obtained as a black solid in slightly lower yields at 38% and 69% for yttrium and samarium, respectively (Scheme 1). The ^1^H NMR spectra of 2-Y and 3-Y are both diamagnetic, consistent with a singlet ground state. The ^1^H NMR spectrum of 2-Y in C_6_D_6_ features an upfield shifted peak for the protons of the bound benzene at 2.37 ppm, which appears as a triplet due to weak ^89^Y–^1^H coupling (*J*_Y–H_ = 1.6 Hz), indicating significant weakening of the aromatic ring current of the bound benzene. The ^13^C NMR spectrum of 2-Y in C_6_D_6_ also shows an upfield shifted triplet for the bound benzene at 65.7 ppm with a *J*_Y–C_ value of 4.8 Hz. In addition, the ^1^H and ^13^C NMR spectra of 3-Y in C_6_D_6_ exhibit a triplet at 2.71 ppm (*J*_Y–H_ = 1.7 Hz), and two triplets at 73.0 (CMe, *J*_Y–C_ = 6.8 Hz) and 71.1 (CH, *J*_Y–C_ = 4.0 Hz) ppm, respectively, similar to 2-Y. Notably, the peak of the two methyl groups of the bound *p*-xylene also shifts upfield to around 0.80 ppm, which is likely due to close proximity to the π face of the aryl group of the BDI ligand (Fig. S18†). For comparison, the ^1^H and ^13^C NMR signals of the bound ring in [K(18-crown-6)(THF)_2_]_2_[[(NN^TBS^)Y]_2_(μ-η^6^,η^6^-C_6_H_5_Ph)] appear at the range of 3.03–3.83 ppm and 54.0–78.8 ppm (*J*_Y–C_ = 6.3 Hz for the peak at 54.0 ppm), respectively.^20^ The similarity of ^1^H and ^13^C NMR signals of the bound ring for 2-Y and 3-Y with [K(18-crown-6)(THF)_2_]_2_[[(NN^TBS^)Y]_2_(μ-η^6^,η^6^-C_6_H_5_Ph)] suggests they may share the same electronic structure as [Y^3+^–(arene)^4−^–Y^3+^]. Moreover, while the ^1^H NMR spectra of 2-Sm and 3-Sm are less informative due to the paramagnetism of Sm^3+^ ions, the proton signal of the bound ring could be observed as a relatively sharp singlet at 21.73 and 26.70 ppm, respectively, which was further validated by the absence of the signal for 2-Sm synthesized in C_6_D_6_. Notably, immediately after dissolution in C_6_D_6_, 3-Sm rapidly underwent arene exchange to form [(BDI)Sm(THF)]_2_(μ-η^6^,η^6^-C_6_D_6_) and free *p*-xylene (Fig. S10†). The ^1^H NMR of 3-Sm could be satisfactorily obtained by measuring the spectrum immediately after dissolution in toluene-*d*_8_ (Fig. S11†), owing to the slower arene exchange rate between 3-Sm and toluene. In contrast, 2-Sm showed no evidence of arene exchange in C_6_D_6_ even after pro-long heating at 50 °C, indicating higher stability of 2-Sm than 3-Sm. Similar stability difference between the parent benzene and alkyl substituted benzenes has previously been observed in inverse-sandwich uranium and thorium arene complexes,^25,42^ which may be attributed to the stronger δ acceptor character of the parent benzene than alkylbenzenes.

**Fig. 2:**
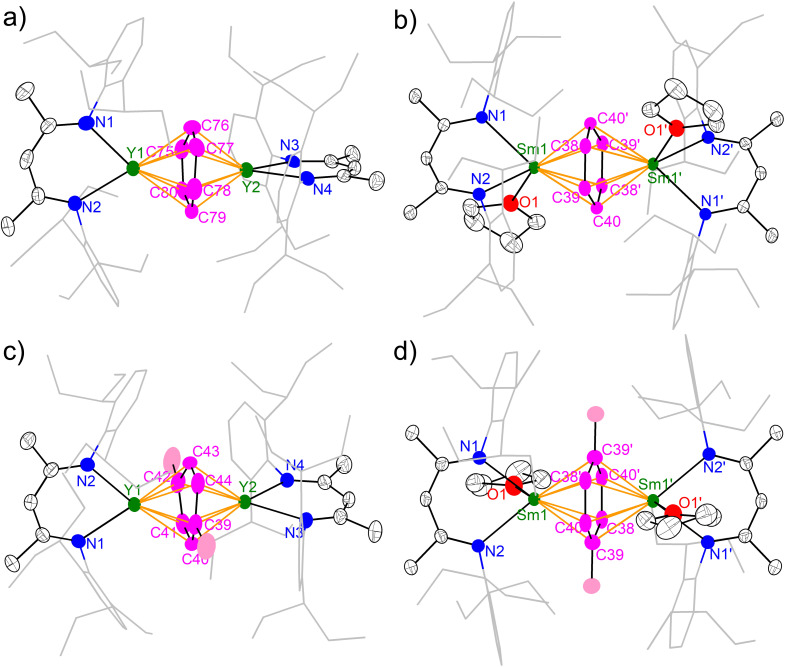
X-ray crystal structures of 2-Y (a), 2-Sm (b), 3-Y (c) and 3-Sm (d) depicted as 50% probability ellipsoids. Hydrogens are omitted and the aryl substituents are shown as wireframe for clarity. For 2-Y, only one of the crystallographically independent molecules in an unsymmetric unit is shown.

The molecular structures of 2-M and 3-M were unambiguously established by single crystal X-ray diffraction and are depicted in Fig. 2. All four complexes are neutral molecules without a counter cation, featuring an inverse-sandwich structure with a μ-η^6^,η^6^ coordination mode for the bound arene ligand. A major difference between yttrium and samarium complexes is the latter containing a coordinated THF molecule per samarium, which is probably due to the larger ionic radius of samarium. Moreover, the coordination of THF causes a conformation change: while the two N–Y–N planes are almost orthogonal to each other in 2-Y (84.0(1)°) and 3-Y (85.1(1)°), the two N–Sm–N planes are co-planar in 2-Sm and 3-Sm as enforced by a crystallographic inversion centre. This also results in a difference in the dihedral angle between the N–M–N planes and the bound arene. While the N–Y–N planes are nearly perpendicular to the bound arene plane (2-Y 88.6(1)–89.6(1)°; 3-Y 88.5(1)–89.8(1)°), the dihedral angle between the N–Sm–N plane and the bound arene significantly deviates from orthogonality (2-Sm 57.12(4)°; 3-Sm 60.95(8)°). Similar conformation change from perpendicular to co-planar accompanied with THF coordination has previously been observed in the series of [(NN^TBS^)Th(THF)_*n*_]_2_(μ-η^6^,η^6^-arene) (*n* = 0 or 1).^42^ Despite the difference in the conformation, the arrangement of M–C_cent_–M (C_cent_ = the ring centroid of the bound arene) is close to linearity (Y–C_cent_–Y 178.70(2)–179.58(3)°, Sm–C_cent_–Sm 180.00°), which is the same as [(NN^TBS^)Th(THF)_n_]_2_(μ-η^6^,η^6^-arene) and indicative of similar electronic structures for these inverse-sandwich f-block metal arene complexes.

**Fig. 3:**
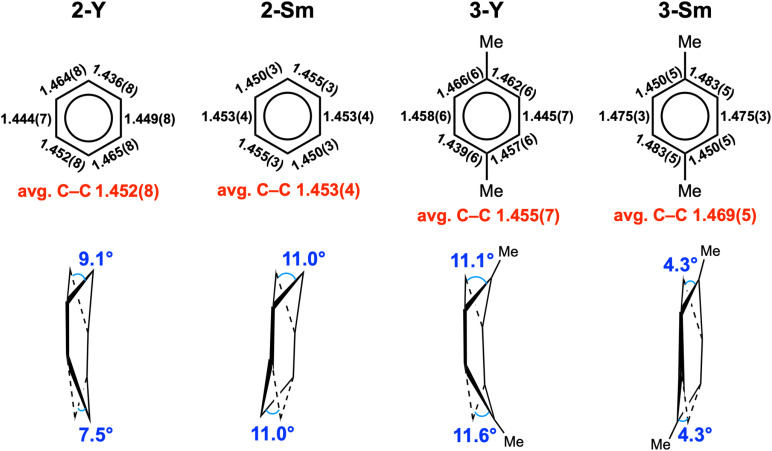
The structural features of the bound rings in 2-M and 3-M.

The key structural parameters are summarized in Table 1. The average Y–N distances in 2-Y (2.378(5) Å) and 3-Y (2.398(3) Å) are *ca.* 0.1 Å longer than that in 1-Y (2.272(5) Å). For samarium complexes, the average Sm–N distances in 2-Sm (2.510(2) Å) and 3-Sm (2.532(2) Å) are elongated by *ca.* 0.2 Å compared with that of 1-Sm (2.311(4) Å). Such elongation between the metal and the nitrogen donors of the ancillary ligand has previously been observed in inverse-sandwich f-block metal arene complexes supported by ferrocene diamide ligands^20,22,28,42^ as well as inverse-sandwich uranium benzene complexes supported by the BDI′ ligand,^36^ which was attributed to the weakening of M–N bonds due to the strong bonding interaction between the metal and the bound arene. In addition, the larger elongation in 2-Sm and 3-Sm may also be caused by the additional THF coordination, since the increase of coordination number leads to a larger ionic radius for Sm^3+^ (the ionic radius increases about 0.06 Å when the coordination number increases one).^60^ The average Y–C_cent_ distances of 2.000(1) (2-Y) and 2.011(1) Å (3-Y) are appreciably shorter than that of [(NN^TBS^)Y]_2_(μ-η^6^,η^6^-C_6_H_5_Ph)[K(toluene)]_2_ (2.093(1) Å) and [K(18-crown-6)(THF)_2_]_2_[[(NN^TBS^)Y]_2_(μ-η^6^,η^6^-C_6_H_5_Ph)] (2.053(1) Å),^20^ indicating stronger metal–arene interactions in neutral inverse-sandwich rare-earth metal arene complexes than anionic ones. In addition, the average Sm–C_cent_ distances of 2.095(1) (2-Sm) and 2.109(1) Å (3-Sm) are appreciably shorter than that of [(NN^TBS^)Sm]_2_(μ-η^6^,η^6^-C_6_H_5_Ph)[K(toluene)]_2_ (2.196(7) Å) and [K(18-crown-6)(THF)_2_]_2_[[(NN^TBS^)Sm]_2_(μ-η^6^,η^6^-C_6_H_5_Ph)] (2.146(8) Å),^22^ consistent with stronger Sm–arene interactions in the neutral compounds as well as the assignment of the +3 oxidation state for samarium. The C–C distances within the bound ring have been correlated with the extent of reduction of the bound arene as well as the distribution of negative charges.^12,13,61^ The average C–C distances of 2-M (Y 1.452(8) Å; Sm 1.453(4) Å) and 3-M (Y 1.455(7) Å; Sm 1.469(5) Å) are significantly longer than that of free benzene (1.39 Å),^62^ but close to the average C–C distances of the bound ring in [(NN^TBS^)Y]_2_(μ-η^6^,η^6^-C_6_H_5_Ph)[K(toluene)]_2_ (1.46 Å) and [K(18-crown-6)(THF)_2_]_2_[[(NN^TBS^)Y]_2_(μ-η^6^,η^6^-C_6_H_5_Ph)] (1.47 Å),^20^ as well as [(NN^TBS^)Sm]_2_(μ-η^6^,η^6^-C_6_H_5_Ph)[K(toluene)]_2_ (1.45 Å), [K(18-crown-6)(THF)_2_]_2_[[(NN^TBS^)Sm]_2_(μ-η^6^,η^6^-C_6_H_5_Ph)] (1.46 Å),^22^ [(NN^TBS^)Th]_2_(μ-η^6^,η^6^-C_6_H_6_) (1.46 Å), and [(NN^TBS^)Th]_2_(μ-η^6^,η^6^-C_6_H_5_Me) (1.45 Å),^42^ in line with quadruply reduced bridging arene ligands in 2-M and 3-M. Moreover, the C–C distances of the bound ring are all within a relatively narrow range for 2-M and 3-M (Table 1, entry 5 & Fig. 3), suggesting the negative charges are evenly distributed among six carbons of the bound ring. This is in accord with previously reported inverse-sandwich f-block metal arene complexes with a [M^3/4+^–(arene)^4−^–M^3/4+^] electronic structure,^20–22,42^ but different from the benzene 1,4-dianion with “two-short, four-long C–C distances” in several rare-earth and alkaline-earth metal benzene complexes.^12,49–51,61^

**Table 1:** Key structural parameters for 2-M and 3-M

Distance (Å), angles (°)	2-Y^a^	2-Sm	3-Y	3-Sm
M–N^a^	2.378(5)	2.510(2)	2.398(3)	2.532(2)
M–C_cent_	2.000(1)	2.095(1)	2.011(1)	2.109(1)
M–C	2.393(5)–2.534(4)	2.493(2)–2.602(2)	2.393(4)–2.595(4)	2.526(4)–2.598(3)
	Avg. 2.464(5)	Avg. 2.565(2)	Avg. 2.479(4)	Avg. 2.570(4)
M–O	NA	2.576(2)	NA	2.585(4)
C–C (ring)	1.436(8)–1.465(8)	1.450(3)–1.455(3)	1.439(6)–1.466(6)	1.450(5)–1.483(5)
	Avg. 1.452(8)	Avg. 1.453(4)	Avg. 1.455(7)	Avg. 1.469(5)
M–C_cent_–M	179.24(3)	180.00	178.70(2)	180.00
Torsion angle^b^	9.1/7.5	11.0/11.0	11.1/11.6	4.3/4.3

a Average values.

b For the boat conformation of 2-Y and 3-Y, defined by the plane of the bridgehead carbon atoms and the neighbouring two carbon atoms and the average plane of other four carbon atoms; for the chair conformation of 2-Sm and 3-Sm, defined by the average plane of the central four carbon atoms and the plane defined by the carbon atoms above and below it together with neighbouring two carbon atoms (see Fig. 3 for illustration).

Notably, the bound rings are not fully planar in 2-M and 3-M, as illustrated in Fig. 3. For 2-Y and 3-Y, the bound rings exhibit a boat conformation with dihedral angles of 9.1°/7.5° (2-Y) and 11.1°/11.6° (3-Y) (defined by the plane of the bridgehead carbon atom and the two neighbouring carbon atoms with the average plane of other four carbon atoms). In contrast, for 2-Sm and 3-Sm, the bound rings adopt a chair conformation with dihedral angles of 11.0°/11.0° (2-Sm) and 4.3°/4.3° (3-Sm) (defined by the average plane of the central four carbon atoms with the plane of the carbon atoms above (or below) the central plane and the neighbouring two carbon atoms). The distortion of the bound rings from planarity has been previously observed in the inverse-sandwich rare-earth metal biphenyl complexes^20–22^ and thorium arene complexes.^42^ Taking into account the above mentioned structural features, we consider that it is appropriate to assign a [M^3+^–(arene)^4−^–M^3+^] electronic structure for 2-M and 3-M. To the best of our knowledge, 2-Y and 2-Sm represent the first rare-earth metal complexes containing the parent benzene tetraanion.

### Spectroscopic and magnetic characterization

**Fig. 4:**
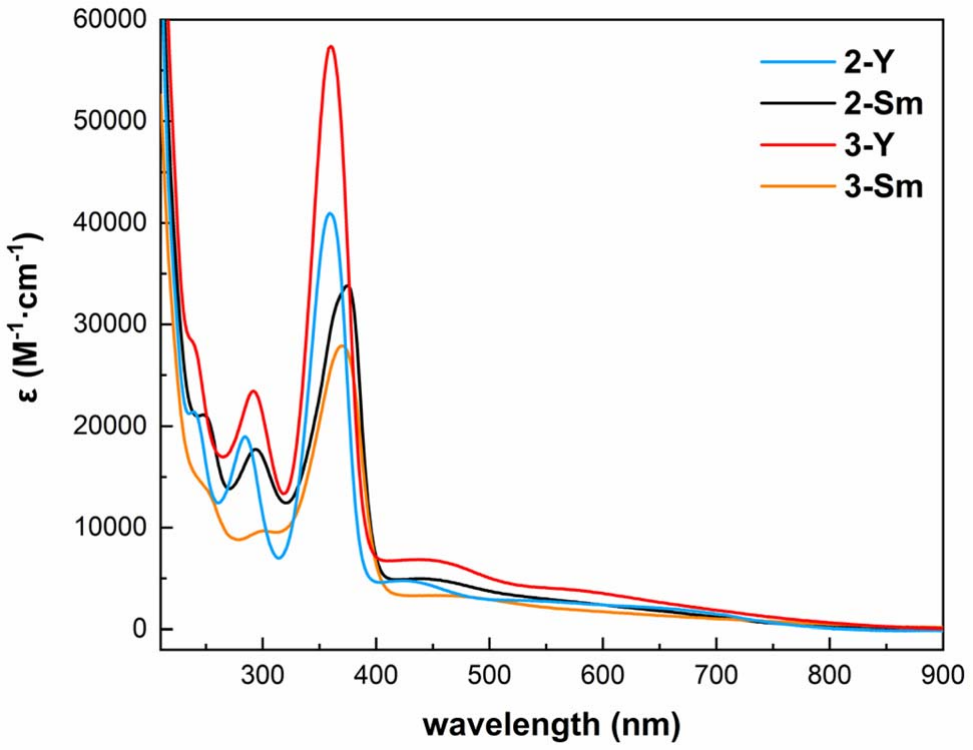
UV-vis-NIR spectra of 2-Y (blue), 2-Sm (black), 3-Y (red) and 3-Sm (orange) in *n*-pentane (298 K, 0.5 mM, 220–900 nm).

In order to further investigate the electronic structures of these inverse-sandwich rare-earth metal arene complexes, we performed the spectroscopic and magnetic measurements. The UV-vis-NIR absorption spectra of 2-Y, 3-Y, 2-Sm and 3-Sm were recorded in *n*-pentane (Fig. 4 and S24–S27†). All four complexes share similar features: (1) sharp intense peaks in the UV region (around 290 nm and 360–370 nm, *ε* > 10^4^ M^−1^ cm^−1^) likely originate from the BDI ligand, since they are also present in 1-M (Fig. S22 and S23†); (2) broad bands covering the entire visible region (*ε* ∼1000–5000 M^−1^ cm^−1^) may be attributed to ligand to metal charge transfer, which are similar to the inverse-sandwich samarium biphenyl complexes,^22^ but bathochromically shifted compared to the inverse-sandwich thorium arene complexes.^42^ The room temperature solution magnetic moment of 2-Sm was determined by the Evans method^63^ to be 0.49 emu mol^−1^ K and 1.98*μ*_B_ (1.40*μ*_B_ per samarium). This value is comparable to the room temperature solution magnetic moments of [(NN^TBS^)Sm]_2_(μ-η^6^,η^6^-C_6_H_5_Ph)[K(toluene)]_2_ (2.47 *μ*_B_, 1.75 *μ*_B_ per samarium) and [K(18-crown-6)(THF)_2_]_2_[[(NN^TBS^)Sm]_2_(μ-η^6^,η^6^-C_6_H_5_Ph)] (2.39 *μ*_B_, 1.69 *μ*_B_ per samarium),^22^ and falls in the normal range of Sm^3+^ (1.3–1.9 *μ*_B_), but differs substantially from the typical range of Sm^2+^ (3.4–3.8 *μ*_B_).^64–70^ These results are consistent with the assignment of a benzene tetraanion bridging between two 4f^5^ Sm(iii) ions, *i.e.* [Sm^3+^–(C_6_H_6_)^4−^–Sm^3+^]. In addition, the room temperature solution magnetic moment of 3-Sm was measured to be 0.576 emu mol^−1^ K and 2.15 *μ*_B_ (1.52 *μ*_B_ per samarium), close to 2-Sm, which is indicative of a similar electronic structure of [Sm^3+^–(C_8_H_10_)^4−^–Sm^3+^].

### Density functional theory calculations

We performed DFT calculations to further investigate the electronic structures and bonding interactions of 2-M and 3-M. The DFT calculations were conducted on simplified model complexes with the 3-pentyl groups truncated to methyl groups. The geometry optimization of 2-Y and 3-Y was done with a closed-shell ground state, and the calculated and crystal structures match well (Tables S3 and S4†). For 2-Sm and 3-Sm, two possible states, ^11^A and ^13^A, were considered. The optimized structures of 2-Sm and 3-Sm with the ^11^A state match well with the crystal structures (Tables S5 and S6†). For instance, the calculated average Sm–C and Sm–C_cent_ distances of 2-Sm (^11^A) are 2.53 and 2.07 Å, respectively, close to the experimental values (2.565(2) and 2.095(1) Å). However, the optimized structures of 2-Sm and 3-Sm with the ^13^A state show significant elongation for Sm–C and Sm–C_cent_ distances compared to the experimental values (Tables S5 and S6†). Therefore, based on the DFT results, the ground state of 2-Sm and 3-Sm is the ^11^A state, in line with the [Sm^3+^–(arene)^4−^–Sm^3+^] electronic structure suggested by structural, spectroscopic and magnetic characterization.

**Fig. 5:**
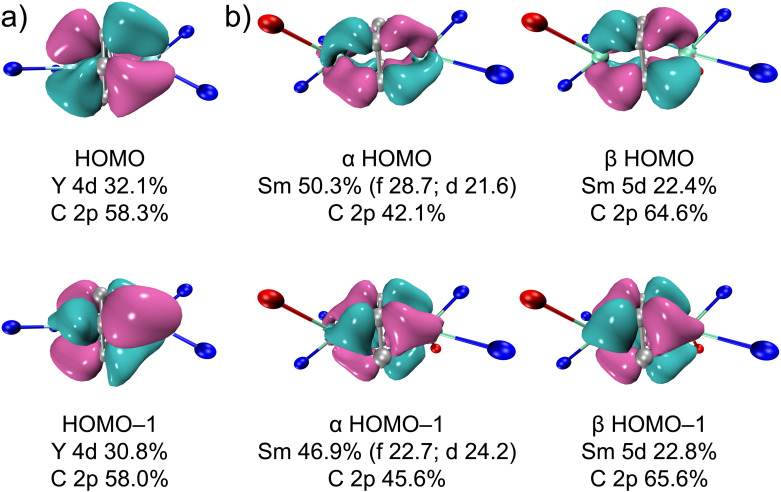
Kohn–Sham orbitals (isovalue 0.04) with composition analysis for the frontier orbitals of 2-Y (a) and 2-Sm (^11^A) (b). Only the metal, the atoms directly bound to the metal, and all carbon atoms of the bound arene are shown for clarity. Nitrogen in blue, oxygen in red, metal in cyan, and carbon in grey.

The highest occupied molecular orbital (HOMO) and HOMO-1 of 2-Y and 2-Sm (both α and β orbitals) are shown in Fig. 5 (3-Y and 3-Sm mostly resemble 2-Y and 2-Sm, see Fig. S38 and S39†). Both HOMO and HOMO-1 feature δ bonding interactions between the rare-earth metals and the bound arene. For 2-Y, the HOMO and HOMO-1 are nearly degenerate (−3.55 and −3.59 eV) and composed of slightly over 30% yttrium 4d orbitals and around 58% carbon 2p orbitals of the bound ring. The higher contribution from yttrium-based orbitals in δ bonding orbitals in 2-Y than that in [[(NN^TBS^)Y]_2_(μ-η^6^,η^6^-C_6_H_5_Ph)]^2–^ (*ca.* 20% of Y 4d orbitals) suggests stronger δ bonding interactions and higher covalency in the former, which is in line with the shorter Y–C_cent_ distance in 2-Y than [(NN^TBS^)Y]_2_(μ-η^6^,η^6^-C_6_H_5_Ph)[K(toluene)]_2_ and [K(18-crown-6)(THF)_2_]_2_[[(NN^TBS^)Y]_2_(μ-η^6^,η^6^-C_6_H_5_Ph)].^20^ In fact, the contribution from yttrium-based orbitals in the δ bonding orbitals in 2-Y is on par with the contribution from the thorium-based orbitals in the δ bonding orbitals in [(NN^TBS^)Th]_2_(μ-η^6^,η^6^-C_6_H_6_) (*ca.* 30%).^42^ Taking into account that both 2-Y and [(NN^TBS^)Th]_2_(μ-η^6^,η^6^-C_6_H_6_) are neutral complexes, and [(NN^TBS^)Y]_2_(μ-η^6^,η^6^-C_6_H_5_Ph)[K(toluene)]_2_ is an ion-pair species, these results support the hypothesis that neutral metal arene complexes will have higher stability over anionic ones due to stronger and more covalent metal–arene δ interactions. For 2-Sm, the α orbitals are slightly lower in energy than the corresponding β orbitals and have some additional 4f characters, which is probably due to energy degeneracy between δ bonding orbitals and 4f orbitals. For comparison, the α orbitals in [[(NN^TBS^)Sm]_2_(μ-η^6^,η^6^-C_6_H_5_Ph)]^2−^ have negligible 4f characters.^22^ The difference in 4f participation in the α orbitals of HOMO and HOMO-1 between 2-Sm and [[(NN^TBS^)Sm]_2_(μ-η^6^,η^6^-C_6_H_5_Ph)]^2−^ may be attributed to the lower energy level of the π* orbitals of biphenyl than that of benzene, which breaks the energy degeneracy between 4f orbitals and δ bonding orbitals. While 3-M mostly resembles 2-M in δ bonding interactions, the lower symmetry of *p*-xylene results in larger energy difference between HOMO and HOMO-1 (Tables S7 and S8†), which may explain the lower stability of 3-M compared to 2-M.

We also performed population analysis on 2-M and 3-M (Tables S9–S13†). The natural population analysis (NPA) charges for the bound benzene (C + H) are −1.87 and −1.56 for 2-Y and 2-Sm, respectively, which are comparable to those of [[(NN^TBS^)M]_2_(μ-η^6^,η^6^-C_6_H_5_Ph)]^2−^ (M = Y, −1.90; M = Sm, −1.56).^20,22^ The NPA charges for yttrium (1.57) and samarium (1.22) are lower than 3, which reflects the covalency in δ bonding interactions. For 3-M, the NPA charges for the bound ring of *p*-xylene and the metal are similar to that of 2-M (Table S13†). The calculated spin density on each samarium is 5.15 for both 2-Sm and 3-Sm, consistent with a 4f^5^ electronic configuration. In addition, the average Wiberg bond index for the C–C bonds of the bound ring is around 1.12 for 2-M and 3-M, indicating significant weakening of the π bonds of the arene compared to neutral arene. The average Wiberg bond indexes for M–C bonds ranging from 0.22–0.27 for 2-M and 3-M are even larger than that of M–N bonds (0.19–0.23) (Table S14†), suggesting relatively strong metal–arene δ interactions. Overall, the population analysis and calculated bond index support the assignment of the [M^3+^–(C_6_H_6_)^4−^–M^3+^] electronic structure for these neutral inverse-sandwich rare-earth metal arene complexes.

### Reactivity studies

**Scheme 2:**
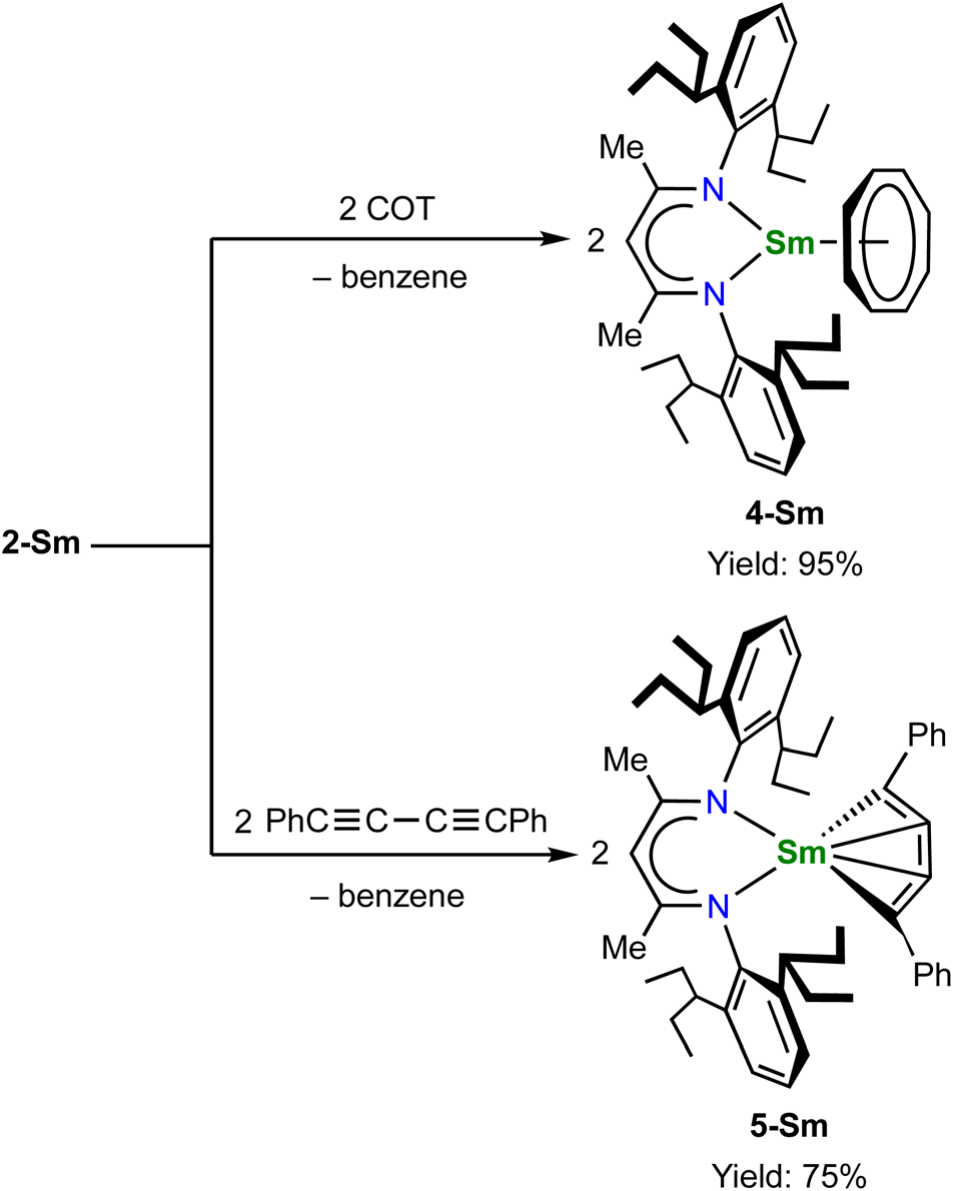
Reactivity of 2-Sm as a four-electron reductant.

**Fig. 6:**
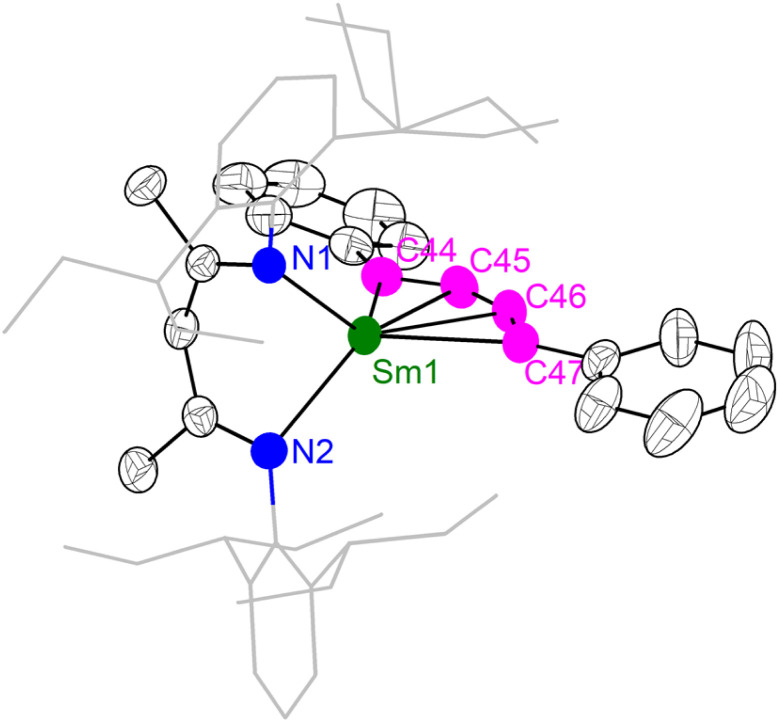
X-ray crystal structure of 5-Sm depicted as 50% probability ellipsoids. Hydrogens are omitted and the aryl substituents are shown as wireframe for clarity. Selected bond distances (Å) and angles (°): Sm1–C44 2.449(3), Sm1–C45 2.446(3), Sm1–C46 2.447(3), Sm1–C47 2.491(3), C44–C45 1.320(5), C45–C46 1.310(4), C46–C47 1.306(5), C44–C45–C46 149.0(4), C47–C46–C45 151.0(3), C44–Sm1–C47 93.0(2).

The unique [M^3+^–(C_6_H_6_)^4−^–M^3+^] electronic structure and the strong metal–arene δ interactions prompted us to explore their potential as multielectron reductants. In addition, the better solubility of neutral compounds in non-polar solvents compared to ion-pair complexes may also be advantageous for reactivity study. We chose 2-Sm as the representative to study its reactivity toward unsaturated organic substrates, since the more readily accessible +2 oxidation state of samarium may play a role in redox reactivity. Treatment of 2-Sm with 2 equiv. of cyclooctatetraene (COT) quantitatively yielded a mononuclear Sm(iii) product (BDI)Sm(η^8^-C_8_H_8_) (4-Sm) with an isolated yield of 95% (Scheme 2). The mononuclear structure of 4-Sm was confirmed by X-ray crystallography (Fig. S20†). In this reaction, two neutral COT molecules are reduced to (COT)^2−^ with the concomitant formation of neutral benzene. The reaction of 2-Sm with 2 equiv. of 1,4-diphenylbutadiyne quantitatively afforded the first rare-earth metallacyclopentatriene complex (BDI)Sm(η^4^-C_4_Ph_2_) (5-Sm) with an isolated yield of 75%, along with the formation of neutral benzene (Scheme 2). The molecular structure of 5-Sm is shown in Fig. 6. The planar structure of the [η^4^-C_4_Ph_2_]^2−^ unit precludes the presence of a 1,4-diphenyl-2-butyne-1,4-diyl species.^71^ The C–C distances for the central four consecutive carbon atoms are 1.320(5), 1.310(4) and 1.306(5) Å, respectively, in line with the [η^4^-C_4_Ph_2_]^2−^ unit being a cumulene. The Sm–C distances for the four carbon atoms are similar at 2.449(3), 2.446(3), 2.447(3) and 2.491(3) Å, consistent with a η^4^ coordination mode. The metal–cumulene bonding scheme in 5-Sm is similar to previously reported actinide metallacyclocumulene complexes,^72–75^ but differs from rare-earth metal [3]cumulene complexes, in which the 1,3-butatrienediyl ligand always bridges between two rare-earth ions in a μ-η^3^,η^3′^ coordination mode.^76–79^ The room temperature magnetic moments of 4-Sm and 5-Sm were determined by the Evans method to be 0.243 emu mol^−1^ K (1.40*μ*_B_) and 0.304 emu mol^−1^ K (1.56*μ*_B_), respectively, in line with the formation of Sm(iii) products. Overall, 2-Sm can serve as a four-electron reductant to reduce unsaturated organic substrates.

## Conclusions

In summary, we synthesized and characterized the rare-earth metal complexes of the parent benzene tetraanion and neutral inverse-sandwich rare-earth metal arene complexes for the first time with a bulky BDI ligand. Structural, spectroscopic and magnetic data agree with a [M^3+^–(C_6_H_6_)^4−^–M^3+^] electronic structure with strong metal–arene interactions. DFT calculation results support the assignment of the [M^3+^–(C_6_H_6_)^4−^–M^3+^] electronic structure and unveil the strong δ bonding interactions between rare-earth metals and the bound arene. Reactivity studies show that the inverse-sandwich samarium benzene complex can act as a four-electron reductant to reduce unsaturated organic substrates. Our results highlight the advantages of bulky monoanionic ligands in stabilizing neutral inverse-sandwich rare-earth metal arene complexes and the potential of these metal arene complexes as multi-electron reservoirs for synthesis and reactivity.

## Data availability

Synthetic procedures, NMR spectra, X-ray crystallographic data, UV-vis-NIR and IR spectra and DFT calculation details are documented in the ESI.† Crystallographic data is available *via* the CCDC.

## Author contributions

Y. W. and Y. Z. synthesized and characterized the compounds. J. L. performed the theoretical calculations. B. T. and C. D. assisted in experiment and synthesized some compounds. Y. W. and C. D. collected and analysed crystallographic data. W. H. acquired fundings. W. H. supervised the study. Y. W., Y. Z., J. L., and W. H. wrote the manuscript with input from all of the authors.

## Conflicts of interest

There are no conflicts to declare.

## Supplementary Material

SC-015-D4SC02491E-s001

SC-015-D4SC02491E-s002

SC-015-D4SC02491E-s003
