# Correction: Neutral inverse-sandwich rare-earth metal complexes of the benzene tetraanion

**DOI:** 10.1039/d4sc90114b

**Published:** 2024-06-24

**Authors:** Yi Wang, Yurou Zhang, Jiefeng Liang, Bowen Tan, Chong Deng, Wenliang Huang

**Affiliations:** a Beijing National Laboratory for Molecular Sciences, College of Chemistry and Molecular Engineering, Peking University Beijing 100871 P. R. China wlhuang@pku.edu.cn

## Abstract

Correction for ‘Neutral inverse-sandwich rare-earth metal complexes of the benzene tetraanion’ by Yi Wang *et al.*, *Chem. Sci.*, 2024, **15**, 8740–8749, https://doi.org/10.1039/D4SC02491E.

The authors regret that one very recently published article and one preprint have not been referenced in the original article. For the samarium benzene complex [(BDI)Sm(THF)]_2_(μ-η^6^,η^6^-C_6_H_6_) (**2-Sm**) reported in our article, two structurally related complexes [(BDI)Sm]_2_(μ-η^6^,η^6^-C_6_H_6_) and [(BDI^Cy^)Sm]_2_(μ-η^6^,η^6^-C_6_H_6_) (BDI^Cy^ = CH[C(Me)NDicyp]_2_, Dicyp = 2,6-dicyclohexylphenyl) have been recently reported in ref. 1 and ref. 2, respectively.

The Royal Society of Chemistry apologises for these errors and any consequent inconvenience to authors and readers.
