# Supplementary material for: Neutral inverse-sandwich rare-earth metal complexes of the benzene tetraanion
Source: Chem Sci. 2024 May 13;15(23):8740–9. doi: 10.1039/d4sc02491e (PMC11185217; doi:10.1039/d4sc02491e)
Supplement: SC-015-D4SC02491E-s002 [file SC-015-D4SC02491E-s002.pdf]

Electronic Supplementary Material (ESI) for Chemical Science.
This journal is © The Royal Society of Chemistry 2024

Supplementary Information for

Neutral Inverse-Sandwich Rare-Earth Metal Complexes of the Benzene Tetraanion

Yi Wang,[†a] Yurou Zhang,[†a] Jiefeng Liang,[a] Bowen Tan,[a,b] Chong Deng,[a] Wenliang Huang*[a]

[a] Beijing National Laboratory for Molecular Sciences, College of Chemistry and Molecular Engineering, Peking University; Beijing, 100871, P. R. China

[b] Present address: Department of Chemistry, Massachusetts Institute of Technology, Cambridge, MA 02139, USA

[†] These authors contributed equally.

Table of Contents

S1. Materials and Methods S3

S2. NMR Spectra S8

S3. X-ray Crystallography S21

S4. UV–Vis–NIR Spectra S33

S5. IR Spectra S41

S6. Density Functional Theory (DFT) Calculations S49

S7. References........ S78

S1. Materials and Methods

S1.1. General Considerations

All experiments were performed under a dry argon atmosphere using standard Schlenk techniques or in a nitrogen filled Vigor glove box unless otherwise specified. Celite (purchased from Sigma-Aldrich) and 4 Å molecular sieves (purchased from Sinopharm) were dried under dynamic vacuum at 250 °C for at least 48 h prior to use. Solvents including hexanes, *n*-pentane, diethyl ether (Et_2O) and toluene, were collected from a Vigor YJC-5 Solvent Purification System under argon, transferred to the glove box without exposure to air, and stored over activated molecular sieves. Tetrahydrofuran (THF) was freshly distilled over sodium benzophenone ketyl and stored over activated molecular sieves prior to use. Deuterated solvents, benzene-d_6 (C_6D_6) and toluene-d_8 (C_7D_8) were obtained from Cambridge Isotope Laboratories, degassed three times using freeze-pump-thaw method and stored over activated molecular sieves for one week prior to use. Super dry benzene was purchased from J&K and stored over activated molecular sieves for one week prior to use. *para*-Xylene (*p*-xylene) was obtained from Energy Chemical, degassed three times using freeze-pump-thaw method and stored over activated molecular sieves for one week prior to use. (BDI)H (HC{C(Me)N[2,6-(3-pentyl-phenyl)]}$_2$),[1] (BDI)K,[2] KC_8,[3] $YI_3(THF)_{3.5}$ and $SmI_3(THF)_{3.5}$[4] were prepared according to literature procedures. All other reagents were purchased from commercial suppliers and used as received.

^{1}H and ^{13}C NMR spectra were recorded on Bruker Avance 400 MHz or 600 MHz spectrometers at room temperature. Variable temperature ^{1}H NMR was recorded on Bruker Avance 500 MHz spectrometers. Chemical shifts are referenced internally to the residual proteo-solvent signal, i.e., C_6D_5H at 7.16 ppm. CHN analyses were performed on a Vario EL elemental analyzer at the Analytical Instrumentation Center of Peking University. The single crystal X-ray diffraction data were collected at 180 K using a Rigaku Oxford diffractometer equipped with a CCD collector using Mo Kα radiation, XtaLAB PRO 007HF(Mo). UV–Vis–NIR spectra were obtained on a SHIMADZU UV3600Plus in a 1 mm quartz cuvette at the Analytical Instrumentation Center of Peking University. Fourier transform infrared (FTIR) spectra in the range of 4000–400 cm^{-1} were recorded on a Bruker Tensor 27 spectrometer using a KBr cell (for solution samples) at the Analytical Center of Peking University.

S1.2. Synthetic Details

Synthesis of (BDI)YI$_2$(THF) (1-Y). (BDI)K (200 mg, 0.35 mmol) was dissolved in 5 mL of Et_2O and $YI_3(THF)_{3.5}$ (305 mg, 0.42 mmol, 1.2 equiv) was dispersed in 2 mL of Et_2O. Both mixtures were cooled at –78 °C for 15 min. The cold solution of (BDI)K was added to the suspension of $YI_3(THF)_{3.5}$ dropwise with stirring. The mixture was warmed to room temperature and stirred for 5 h. The volatiles were removed under reduced pressure and the residue was extracted into hexanes (ca. 10 mL). Then the filtrate was concentrated to ca. 2 mL. After storing at –35 °C for 2 d, colorless crystals precipitated from the solution. The mother liquor was decanted and the crystals were washed with cold *n*-pentane and dried under reduced pressure to yield **1-Y** as a pale yellow solid (Yield: 148 mg, 45%). Single crystals of **1-Y** suitable for X-ray crystallography were grown from a hexanes solution at –35 °C. ^{1}H NMR (400 MHz, C_6D_6) δ, ppm: 7.14–7.04 (m, 6H, C*H* of aryl), 5.17 (s, 1H, CC*H*C), 3.61 (s, 4H,

C*H*($(CH_2CH_3)_2$), 3.28 (s, 4H, OCH_2), 2.01–1.80 (m, 8H, CH_2CH$_3$), 1.77–1.57 (m, 14H, CH_2CH$_3$ (8H) & CCH_3 (6H)), 1.23–1.11 (m, 16H, OCH$_2$CH_2 (4H) & CH$_2$CH_3 (12H)), 0.90 (s, 12H, CH$_2$CH_3). ^{13}C{H} NMR (101 MHz, C_6D_6) δ, ppm: 168.25 (*C*(CH_3)N), 146.62, 139.46, 126.61, 125.13 (*C* of aryl), 96.60 (C*C*HC), 71.92 (O*C*H$_2$), 40.71 (*C*H($(CH_2CH_3)_2$)), 27.36 (*C*H$_2$CH$_3$), 26.99 (OCH$_2$*C*H$_2$), 25.71 (C*C*H$_3$), 24.91 (*C*H$_2$CH$_3$), 11.80 (CH$_2$*C*H$_3$), 10.82 (CH$_2$*C*H$_3$). Anal. (%): Calcd. for $C_{44}H_{71}I_2N_2OY$, M_w = 986.78, as in the formula of (BDI)YI_2(THF)·0.5C_6H_{14}(hexanes): C, 53.56; H, 7.25; N, 2.84. Found: C, 53.77; H, 7.08; N, 2.81.

Synthesis of (BDI)SmI$_2$(THF) (1-Sm). (BDI)K (300 mg, 0.53 mmol) was dissolved in 12 mL of Et_2O and $SmI_3(THF)_{3.5}$ (496 mg, 0.63 mmol, 1.2 equiv) was dispersed in 5 mL of Et_2O. Both were cooled at –78 °C for 15 min. The cold solution of (BDI)K was added to the suspension of $SmI_3(THF)_{3.5}$ dropwise with stirring. The mixture was warmed to room temperature and stirred overnight. The brown suspension was then filtered through Celite and washed with Et_2O (ca. 5 mL × 3). The volatiles were removed under reduced pressure and the residue was extracted into hexanes/Et_2O (ca. 15 mL, 1:1 v/v). A small amount of THF (100 μL) was added to facilitate the dissolution. Then the filtrate was concentrated to ca. 3 mL. After standing at room temperature for 1 h, orange crystals precipitated from the solution. The mother liquor was decanted and the crystals were dried under reduced pressure to yield **1-Sm** as an orange solid (Yield: 292 mg, 55%). Single crystals of **1-Sm** suitable for X-ray crystallography were grown from a Et_2O solution at room temperature. ^{1}H NMR (400 MHz, C_6D_6) δ, ppm: 7.86 (s, 1H, CC*H*C), 7.04 (t, *J* = 7.7 Hz, 2H, *para*-C*H* of aryl), 6.73 (d, *J* = 7.8 Hz, 4H, *meta*-C*H* of aryl), 4.51 (s, 4H, OCH_2), 2.68 (s, 6H, CCH_3), 2.00 (s, 4H, OCH$_2$CH_2), 1.88–1.11 (m, 16H, CH_2CH$_3$), 0.84 (s, 12H, CH$_2$CH_3), 0.65 (s, 4H, C*H*($(CH_2CH_3)_2$)), 0.42 (s, 12H, CH$_2$CH_3). Anal. (%): Calcd. for $C_{42}H_{67.5}I_2N_2O_{1.25}Sm$, M_w = 1024.68, as in the formula of (BDI)SmI_2(THF)·0.25$C_4H_{10}O$(Et_2O): C, 49.23; H, 6.64; N, 2.73. Found: C, 49.41; H, 6.69; N, 2.78.

Synthesis of [(BDI)Y]$_2$(μ–η^6,η^6-C_6H_6) (2-Y). 1-Y (100 mg, 0.11 mmol) and KC_8 (34 mg, 0.25 mmol, 2.4 equiv) were dispersed in 1 mL of C_6H_6. The mixture stirred vigorously at room temperature overnight. The volatiles of resulting black suspension were removed under reduced pressure. The residue was extracted into *n*-pentane (ca. 10 mL) and the volatiles were removed under reduced pressure. The black remaining solid was then extracted into Et_2O (ca. 8 mL) and the filtrate was concentrated to ca. 3 mL. After storing at –35 °C for 10 d, black crystals precipitated from the solution. The mother liquor was decanted and the crystals were washed with cold *n*-pentane and dried under reduced pressure to yield **2-Y** as a black solid (Yield: 39 mg, 56%). Single crystals of **2-Y** suitable for X-ray crystallography were grown from a *n*-pentane solution at –35 °C. ^{1}H NMR (400 MHz, C_6D_6) δ, ppm: 7.12–7.06 (m, 4H, *para*-C*H* of aryl), 6.99 (d, *J* = 7.6 Hz, 8H, *meta*-C*H* of aryl), 4.92 (s, 2H, CC*H*C), 3.03–2.95 (m, 8H, C*H*($(CH_2CH_3)_2$)), 2.37 (t, $J_{Y\text{-}H}$ = 1.6 Hz, 6H, C*H* of the bound benzene), 1.92–1.82 (m, 16H, CH_2CH$_3$), 1.75 (s, 12H, CCH_3), 1.71–1.58 (m, 16H, CH_2CH$_3$), 1.08 (t, *J* = 7.3 Hz, 24H, CH$_2$CH_3), 0.80 (t, *J* = 7.4 Hz, 24H, CH$_2$CH_3). ^{13}C{H} NMR (101 MHz, C_6D_6) δ, ppm: 164.57 (*C*(CH_3)N), 144.56, 140.58, 125.05, 123.96 (*C* of aryl), 96.01 (C*C*HC), 65.74 (*C*H of the bound benzene, $J_{Y\text{-}C}$ = 4.8 Hz), 41.61 (*C*H($(CH_2CH_3)_2$)), 28.40 (*C*H$_2$CH$_3$), 24.50 (*C*H$_2$CH$_3$), 23.99 (*C*H($(CH_2CH_3)_2$)), 11.92 (CH$_2$*C*H$_3$), 10.84 (CH$_2$*C*H$_3$). Anal. (%): Calcd. For $C_{80}H_{120}N_4Y_2$, M_w

= 1315.68, as in the formula of $[(BDI)Y]_2(\mu\text{–}\eta^6,\eta^6\text{-}C_6H_6)$: C, 73.03; H, 9.19; N, 4.26. Found: C, 73.26; H, 9.42; N, 4.12.

Synthesis of $[(BDI)Sm(THF)]_2(\mu\text{–}\eta^6,\eta^6\text{-}C_6H_6)$ (2-Sm). 1-Sm (150 mg, 0.15 mmol) and KC_8 (48 mg, 0.36 mmol, 2.4 equiv) were dispersed in 1.5 mL of C_6H_6. The mixture stirred vigorously at room temperature for 4 h. The volatiles of resulting black suspension were removed under reduced pressure. The residue was extracted into Et_2O (ca. 10 mL) and the filtrate was concentrated to ca. 5 mL. A small amount of THF (50 μL) was added to the solution. After storing at –35 °C overnight, black crystals precipitated from the solution. The mother liquor was decanted and the crystals were dried under reduced pressure to yield **2-Sm** as a black solid (Yield: 89 mg, 76%). Single crystals of **2-Sm** suitable for X-ray crystallography were grown from a *n*-pentane solution containing a few drops of THF at –35 °C. ^{1}H NMR (400 MHz, C_6D_6) δ, ppm: 21.73 (s, 6H, C*H* of the bound benzene), 10.68 (s, 2H, CC*H*C), 6.68 (t, *J* = 7.8 Hz, 4H, *para*-C*H* of aryl), 6.31 (d, *J* = 8.0 Hz, 8H, *meta*-C*H* of aryl), 5.39 (s, 12H, CC*H*$_3$), 2.10–1.95 (m, 8H, C*H*$_2$CH$_3$), 1.45–1.31 (m, 8H, C*H*$_2$CH$_3$), 1.00 (t, *J* = 7.4 Hz, 24H, CH$_2$C*H*$_3$), –0.22 (s, 8H, C*H*(CH$_2$CH$_3$)$_2$), –1.82 (t, *J* = 7.1 Hz, 24H, CH$_2$C*H*$_3$), –2.67 to –2.82 (m, 8H, C*H*$_2$CH$_3$), –5.40 to –5.64 (m, 8H, C*H*$_2$CH$_3$). The peaks of coordinating THF molecules are not observed probably due to the loss of THF molecules under reduced pressure during drying process. Anal. (%): Calcd. For $C_{86}H_{126}N_4Sm_2$, M_w = 1516.70, as in the formula of $[(BDI)Sm]_2(\mu\text{–}\eta^6,\eta^6\text{-}C_6H_6)\cdot C_6H_6$(benzene): C, 68.10; H, 8.37; N, 3.69. Found: C, 67.91; H, 8.70; N, 3.88.

Synthesis of $[(BDI)Y]_2(\mu\text{–}\eta^6,\eta^6\text{-}C_8H_{10})$ (3-Y). 1-Y (150 mg, 0.16 mmol) and KC_8 (52 mg, 0.38 mmol, 2.4 equiv) were dispersed in 2 mL of *p*-xylene. The mixture was stirred vigorously at room temperature for 3 d. The volatiles of resulting black suspension were removed under reduced pressure. The residue was extracted into *n*-pentane (ca. 20 mL) and the filtrate was concentrated to ca. 5 mL. After storing at –35 °C for 7 d, black crystals precipitated from the solution. The mother liquor was decanted and the crystals were washed with cold *n*-pentane and dried under reduced pressure to yield **3-Y** as a black solid (Yield: 41 mg, 38%). Single crystals of **3-Y** suitable for X-ray crystallography were grown from a *n*-pentane solution at –35 °C. ^{1}H NMR (400 MHz, C_6D_6) δ, ppm: 7.05 (s, 12H, C*H* of aryl), 4.93 (s, 2H, CC*H*C), 3.03–2.92 (m, 8H, C*H*(CH$_2$CH$_3$)$_2$), 2.71 (t, $J_{Y\text{–}H}$ = 1.7 Hz, 4H, C*H* of the bound xylene), 2.00–1.77 (m, 16H, C*H*$_2$CH$_3$), 1.74–1.57 (m, 28H, CC*H*$_3$ (12H) & C*H*$_2$CH$_3$ (16H)), 1.14 (t, *J* = 7.3 Hz, 24H, CH$_2$C*H*$_3$), 0.82–0.76 (m, 30H, CH$_2$C*H*$_3$ (24H) & C*H*$_3$ of the bound xylene (6H)). ^{13}C{H} NMR (101 MHz, C_6D_6) δ, ppm: 165.48 (*C*(CH$_3$)N), 146.23, 140.46, 125.49, 123.84 (*C* of aryl), 96.40 (C*C*HC), 73.01 (*C*Me of the bound xylene, $J_{Y\text{–}C}$ = 6.8 Hz), 71.14 (*C*H of the bound xylene, $J_{Y\text{–}C}$ = 4.0 Hz), 41.93 (*C*H(CH$_2$CH$_3$)$_2$), 28.27 (*C*H$_2$CH$_3$), 24.75 (*C*H(CH$_2$CH$_3$)$_2$), 23.77 (*C*H$_2$CH$_3$), 19.05 (*C*H$_3$ of the bound xylene), 12.63 (CH$_2$*C*H$_3$), 10.53 (CH$_2$*C*H$_3$). Anal. (%): Calcd. For $C_{82}H_{124}N_4Y_2$, M_w = 1343.73, as in the formula of $[(BDI)Y]_2(\mu\text{–}\eta^6,\eta^6\text{-}C_8H_{10})$: C, 73.30; H, 9.30; N, 4.17. Found: C, 73.08; H, 9.26; N, 4.12.

Synthesis of $[(BDI)Sm(THF)]_2(\mu\text{–}\eta^6,\eta^6\text{-}C_8H_{10})$ (3-Sm). 1-Sm (100 mg, 0.10 mmol) and KC_8 (32 mg, 0.24 mmol, 2.4 equiv) were dispersed in 1 mL of *p*-xylene. The mixture stirred vigorously at room temperature for 2 d. The volatiles of resulting black suspension were removed under reduced pressure. The residue was extracted into *n*-pentane (ca. 5 mL) and the filtrate was concentrated to ca. 3 mL. A small amount of THF (50 μL) was added to the solution.

After storing at –35 °C overnight, black crystals precipitated from the solution. The mother liquor was decanted and the crystals were washed with cold *n*-pentane and dried under reduced pressure to yield **3-Sm** as a black solid (Yield: 55 mg, 69%). Single crystals of **3-Sm** suitable for X-ray crystallography were grown from a *n*-pentane solution containing a few drops of THF at –35 °C. ^{1}H NMR (400 MHz, C_7D_8) immediately δ, ppm: 26.43 (s, 4H, C*H* of the bound *p*-xylene), 8.80 (s, 2H, CC*H*C), 6.36 (t, *J* = 7.7 Hz, 4H, *para*-C*H* of aryl), 6.19 (d, *J* = 7.7 Hz, 8H, *meta*-C*H* of aryl), 5.17 (s, 12H, CC*H*$_3$), 2.28–2.15 (m, 8H, C*H*$_2$CH$_3$), 1.50–1.35 (m, 8H, C*H*$_2$CH$_3$), 0.98 (t, *J* = 7.4 Hz, 24H, CH$_2$C*H*$_3$), 0.53 (s, 8H, C*H*(CH$_2$CH$_3$)$_2$), 0.20 (t, *J* = 6.9 Hz, 24H, CH$_2$C*H*$_3$), –0.54 to –0.72 (m, 8H, C*H*$_2$CH$_3$), –1.64 to –1.82 (m, 8H, C*H*$_2$CH$_3$), –7.95 (s, 6H, C*H*$_3$ of the bound *p*-xylene). The peaks of coordinating THF molecules are not observed probably due to the loss of THF molecules under reduced pressure during drying process. Anal. (%): Calcd. for $C_{82}H_{124}N_4Sm_2$, M_w = 1466.64, as in the formula of [(BDI)Sm]$_2$(μ–η^6,η^6-C_8H_{10}): C, 67.15; H, 8.52; N, 3.82. Found: C, 67.11; H, 8.73; N, 3.67.

Arene exchange reaction of 3-Sm. 3-Sm (6 mg, 0.004 mmol) was dissolved in C_6D_6 (ca. 0.6 mL) and the black solution was transferred to a J. Young tube. The arene exchange reaction was monitored by ^{1}H NMR spectroscopy at room temperature over the course of 24 h. There was significant exchange after 5 min (Figure S9). The reaction reached completion and **3-Sm** was fully converted to [(BDI)Sm(THF)]$_2$(μ–η^6,η^6-C_6D_6) after 24 h (Figure S10).

Synthesis of (BDI)Sm(η^8-COT) (4-Sm). 2-Sm (80 mg, 0.05 mmol) and cyclooctatetraene (COT) (13 mg, 0.13 mmol, 2.5 equiv) were dissolved in 6 mL and 2 mL of Et_2O, respectively. Both solutions were cooled at –78 °C for 15 min. The cold solution of **2-Sm** was added to the solution of COT dropwise with stirring. The mixture was warmed to room temperature and stirred for 30 min. The volatiles of resulting brown solution were removed under reduced pressure. The residue was extracted into *n*-pentane (ca. 8 mL) and the filtrate was concentrated to ca. 3 mL. A small amount of THF (30 μL) was added to the solution. After standing at room temperature for 1 h, light purple solid precipitated from the solution. The mother liquor was decanted and the solid was dried under reduced pressure to yield **4-Sm** as a light purple solid (Yield: 82 mg, 95%). Single crystals of **4-Sm** suitable for X-ray crystallography were grown from a Et_2O solution at –35 °C. ^{1}H NMR (400 MHz, C_6D_6) δ, ppm: 8.20 (s, 8H, C*H* of COT), 7.99 (t, *J* = 7.8 Hz, 2H, *para*-C*H* of aryl), 7.77 (s, 1H, CC*H*C), 7.49 (d, *J* = 7.8 Hz, 4H, *meta*-C*H* of aryl), 3.58 (s, 10H, CC*H*$_3$ & OC*H*$_2$ of free THF), 1.72–1.59 (m, 4H, C*H*$_2$CH$_3$), 1.45–1.37 (m, 4H, OCH$_2$C*H*$_2$ of free THF), 1.00–0.89 (m, 4H, C*H*$_2$CH$_3$), 0.70 (t, *J* = 7.4 Hz, 12H, CH$_2$C*H*$_3$), –1.46 (t, *J* = 7.2 Hz, 12H, CH$_2$C*H*$_3$), –1.78 to –1.92 (m, 4H, C*H*$_2$CH$_3$), –1.99 to –2.11 (m, 4H, C*H*$_2$CH$_3$), –2.21 (s, 4H, C*H*(CH$_2$CH$_3$)$_2$). Anal. (%): Calcd. For $C_{51.5}H_{79}N_2OSm$, M_w = 878.54, as in the formula of (BDI)Sm(η^8-COT)·THF·0.5C_5H_{12}(*n*-pentane): C, 69.30; H, 8.92; N, 3.14. Found: C, 69.22; H, 8.89; N, 3.34.

Synthesis of (BDI)Sm(η^4-C_4Ph_2) (5-Sm). 2-Sm (25 mg, 0.016 mmol) and 1,4-diphenylbutadiyne (8 mg, 0.038 mmol, 2.4 equiv) were each dissolved in 1 mL of Et_2O. Both solutions were cooled at –78 °C for 15 min. The cold solution of **2-Sm** was added to the solution of 1,4-diphenylbutadiyne dropwise with stirring. The mixture was warmed to room temperature and stirred for 1 h. The volatiles of resulting red brown solution were removed under reduced pressure. The residue was extracted into *n*-pentane (ca. 3 mL) and the filtrate was concentrated to ca. 1 mL. After storing at –78 °C for 1 h, red crystals precipitated from the

solution. The mother liquor was decanted and the crystals were washed with cold *n*-pentane. The remaining solid was dried under reduced pressure to yield **5-Sm** as a dark red solid (Yield: 21 mg, 75%). Single crystals of **5-Sm** suitable for X-ray crystallography were grown from a *n*-pentane solution at –35 °C. ^{1}H NMR (400 MHz, C_6D_6) δ, ppm: 15.23 (s, 1H, CC*H*C), 13.39 (d, J = 7.4 Hz, 4H), 8.42 (t, J = 7.0 Hz, 4H, *meta*-C*H* of C_4Ph_2), 8.22 (t, J = 7.2 Hz, 2H), 5.15 (t, J = 7.6 Hz, 2H), 4.10 (s, 4H), 2.18 (s, 6H, CCH_3), 0.25 (s, 12H, CH_2CH_3), –0.26 to –1.07 (m, 20H, CH_2CH_3 (12H) & CH_2CH_3 (8H)), –2.69 (s, 4H), –3.99 (s, 4H), –12.01 (br s, 4H). The signals cannot be completely assigned due to the signal broadening and similar signal splitting in aryl groups of the ligand backbone and the [C_4Ph_2] unit. Anal. (%): Calcd. For $C_{53}H_{67}N_2Sm$, M_w = 882.49, as in the formula of (BDI)Sm(η^4-C_4Ph_2): C, 72.13; H, 7.65; N, 3.17. Found: C, 72.27; H, 7.66; N, 3.00.

S2. NMR Spectra

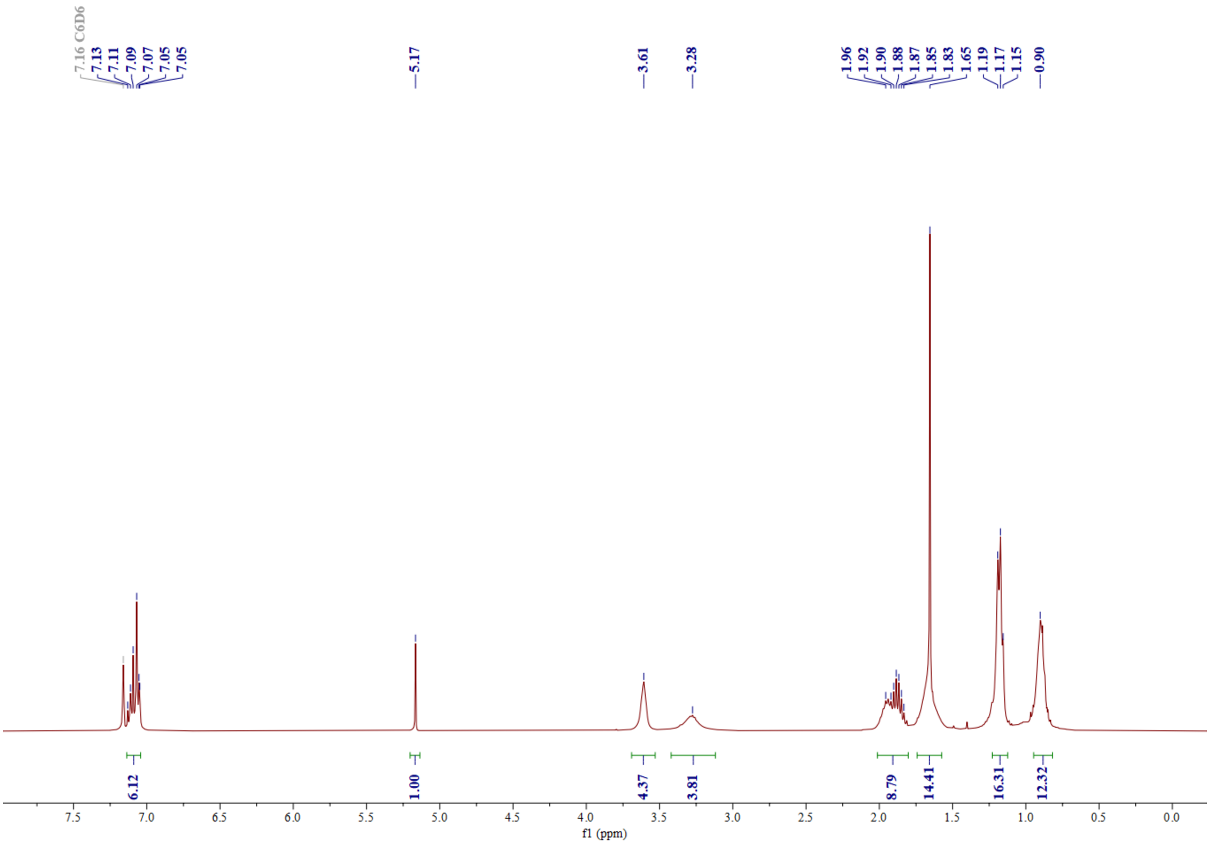

Fig. S1. ^{1}H NMR (400 MHz, C_6D_6, 298K) spectrum of (BDI)YI_2(THF) (**1-Y**), δ, ppm: 7.14–7.04 (m, 6H, C*H* of aryl), 5.17 (s, 1H, CC*H*C), 3.61 (s, 4H, C*H*(CH_2CH_3)$_2$), 3.28 (s, 4H, OCH_2), 2.01–1.80 (m, 8H, C$H_2$$CH_3$), 1.77–1.57 (m, 14H, C$H_2$$CH_3$ (8H) & CCH_3 (6H)), 1.23–1.11 (m, 16H, OCH_2CH_2 (4H) & CH_2CH_3 (12H)), 0.90 (s, 12H, CH_2CH_3).

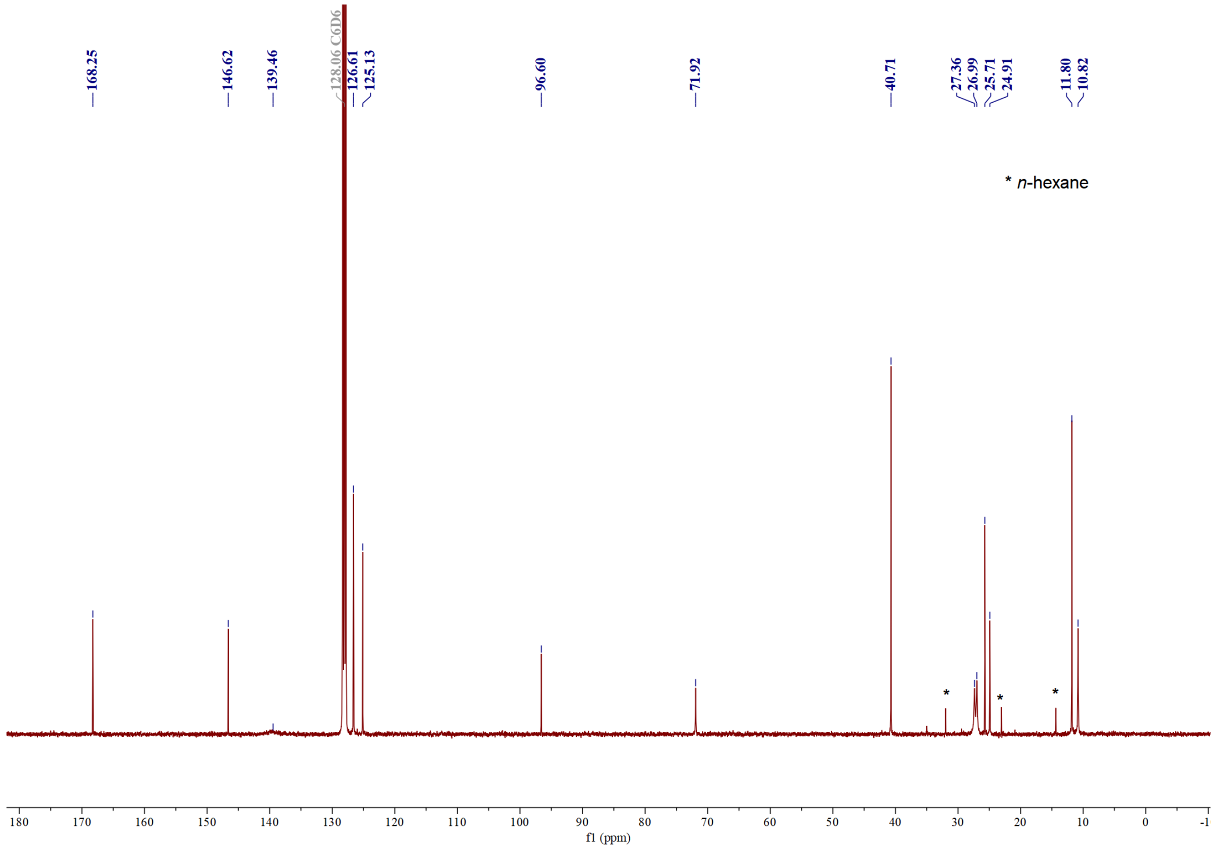

Fig. S2. ^{13}C{H} NMR (101 MHz, C_6D_6, 298K) spectrum of (BDI)YI_2(THF) (**1-Y**), δ, ppm: 168.25 (*C*(CH_3)N), 146.62, 139.46, 126.61, 125.13 (*C* of aryl), 96.60 (C*C*HC), 71.92 (O*C*H_2), 40.71 (*C*H(CH_2CH_3)$_2$), 27.36 (*C*H_2CH_3), 26.99 (OCH_2*C*H_2), 25.71 (C*C*H_3), 24.91 (*C*H_2CH_3), 11.80 (CH_2*C*H_3), 10.82 (CH_2*C*H_3).

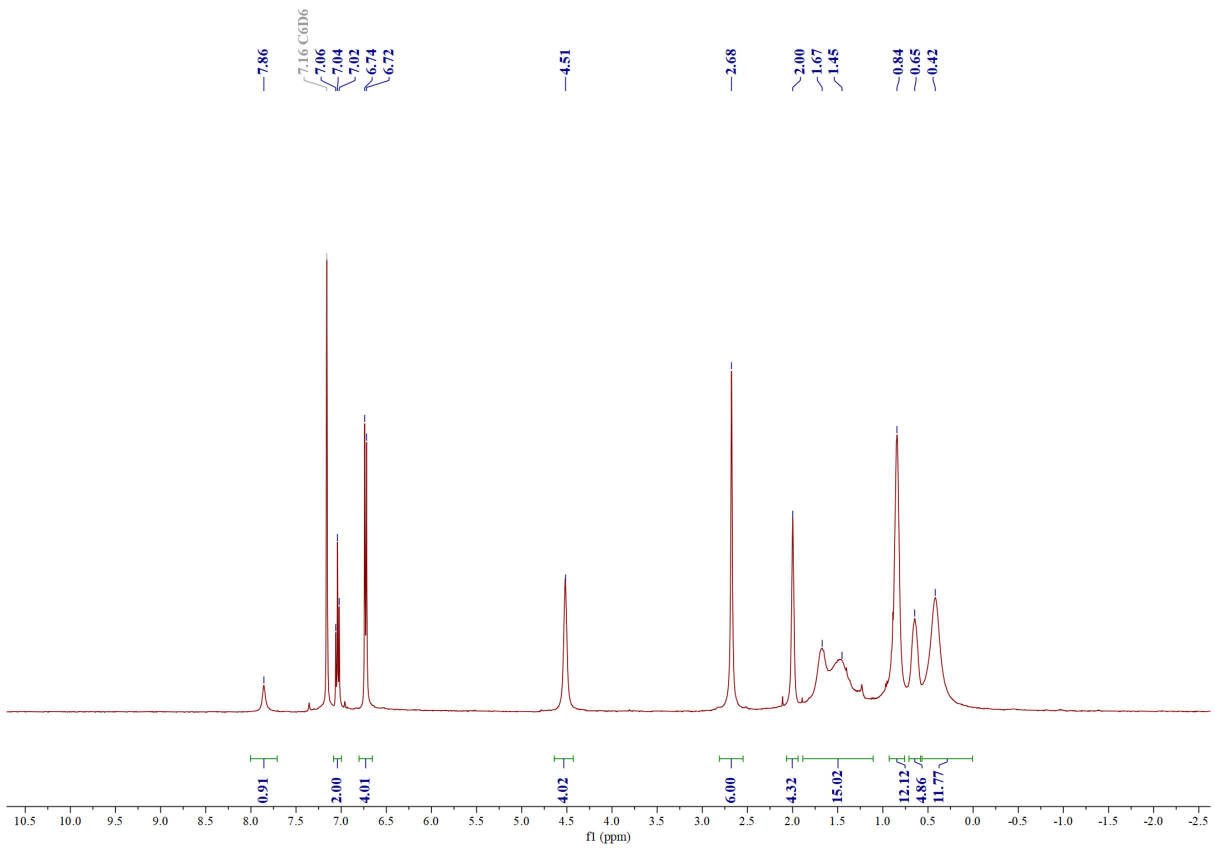

Fig. S3. ^{1}H NMR (400 MHz, C_6D_6, 298K) spectrum of (BDI)SmI_2(THF) (**1-Sm**), δ, ppm: 7.86 (s, 1H, CC*H*C), 7.04 (t, *J* = 7.7 Hz, 2H, *para*-C*H* of aryl), 6.73 (d, *J* = 7.8 Hz, 4H, *meta*-C*H* of aryl), 4.51 (s, 4H, OCH_2), 2.68 (s, 6H, CCH_3), 2.00 (s, 4H, OCH_2CH_2), 1.88–1.11 (m, 16H, CH_2CH_3), 0.84 (s, 12H, CH_2CH_3), 0.65 (s, 4H, C*H*(CH_2CH_3)$_2$), 0.42 (s, 12H, CH_2CH_3).

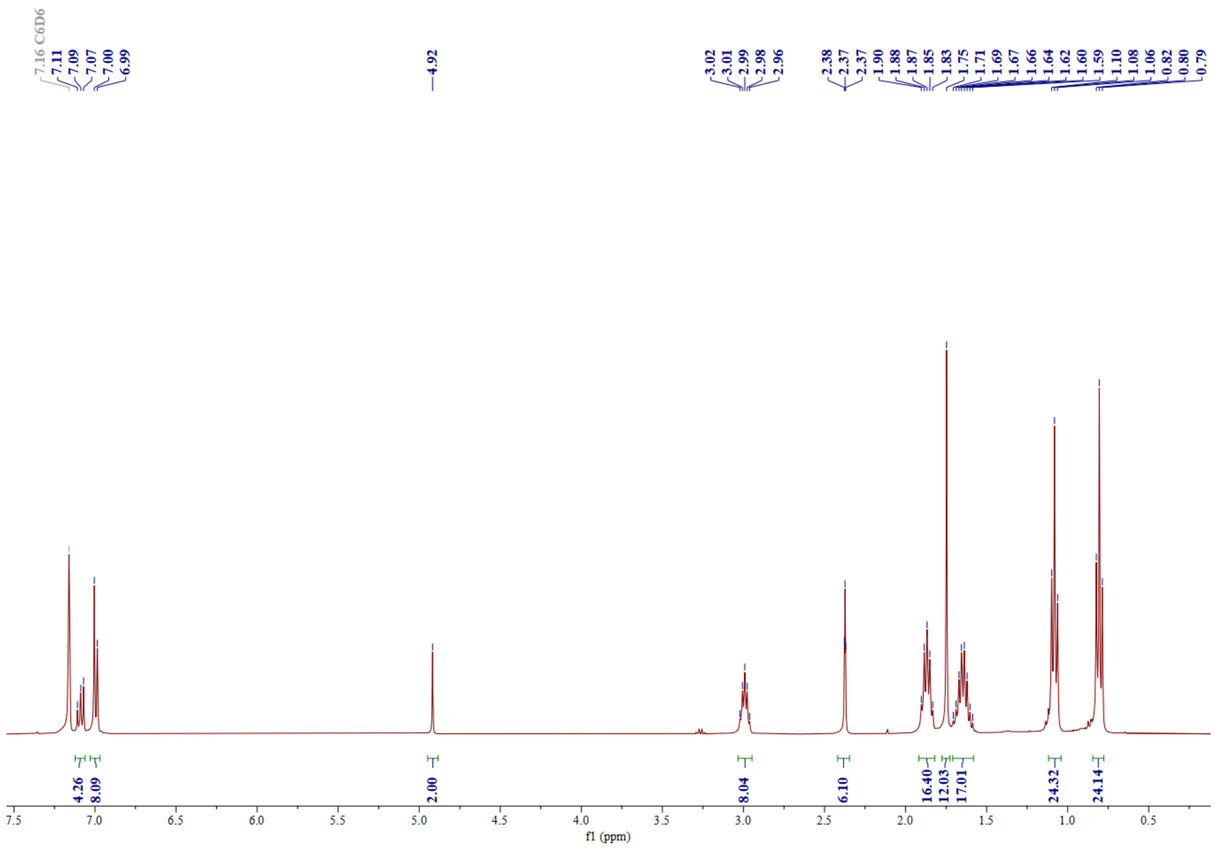

Fig. S4. ^{1}H NMR (400 MHz, C_6D_6, 298K) spectrum of $[(BDI)Y]_2(\mu\text{–}\eta^6,\eta^6\text{-}C_6H_6)$ (**2-Y**), δ, ppm: 7.12–7.06 (m, 4H, *para*-C*H* of aryl), 6.99 (d, J = 7.6 Hz, 8H, *meta*-C*H* of aryl), 4.92 (s, 2H, CC*H*C), 3.03–2.95 (m, 8H, C*H*(CH_2CH_3)$_2$), 2.37 (t, $J_{Y\text{-}H}$ = 1.6 Hz, 6H, C*H* of the bound benzene), 1.92–1.82 (m, 16H, C$H_2$$CH_3$), 1.75 (s, 12H, CC$H_3$), 1.71–1.58 (m, 16H, C$H_2$$CH_3$), 1.08 (t, J = 7.3 Hz, 24H, CH_2CH_3), 0.80 (t, J = 7.4 Hz, 24H, CH_2CH_3).

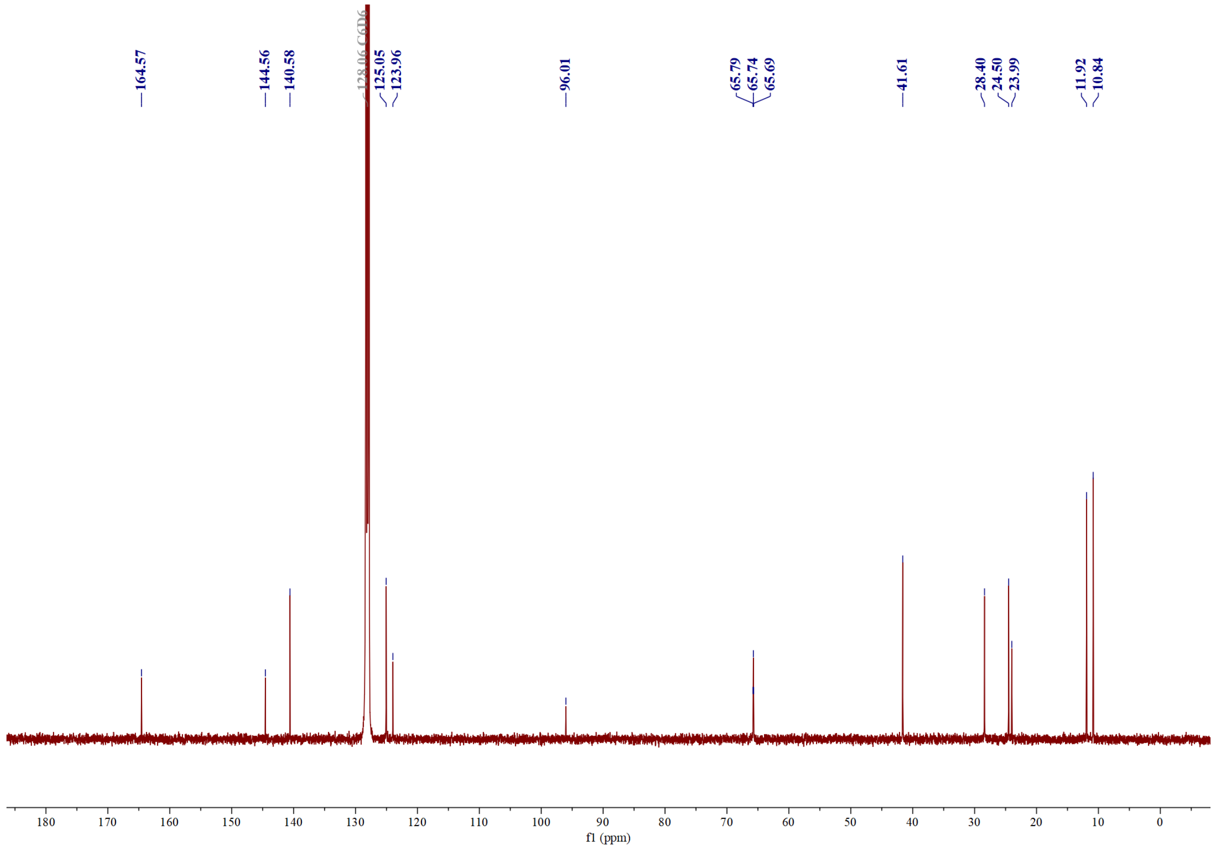

Fig. S5. $^{13}C\{H\}$ NMR (101 MHz, C_6D_6, 298K) spectrum of $[(BDI)Y]_2(\mu\text{–}\eta^6,\eta^6\text{-}C_6H_6)$ (**2-Y**), δ, ppm: 164.57 (*C*(CH_3)N), 144.56, 140.58, 125.05, 123.96 (*C* of aryl), 96.01 (C*C*HC), 65.74 (*C*H of the bound benzene, $J_{Y\text{-}C}$ = 4.8 Hz), 41.61 (*C*H(CH_2CH_3)$_2$), 28.40 (*C*H_2CH_3), 24.50 (*C*H_2CH_3), 23.99 (*C*H(CH_2CH_3)$_2$), 11.92 (CH_2*C*H_3), 10.84 (CH_2*C*H_3).

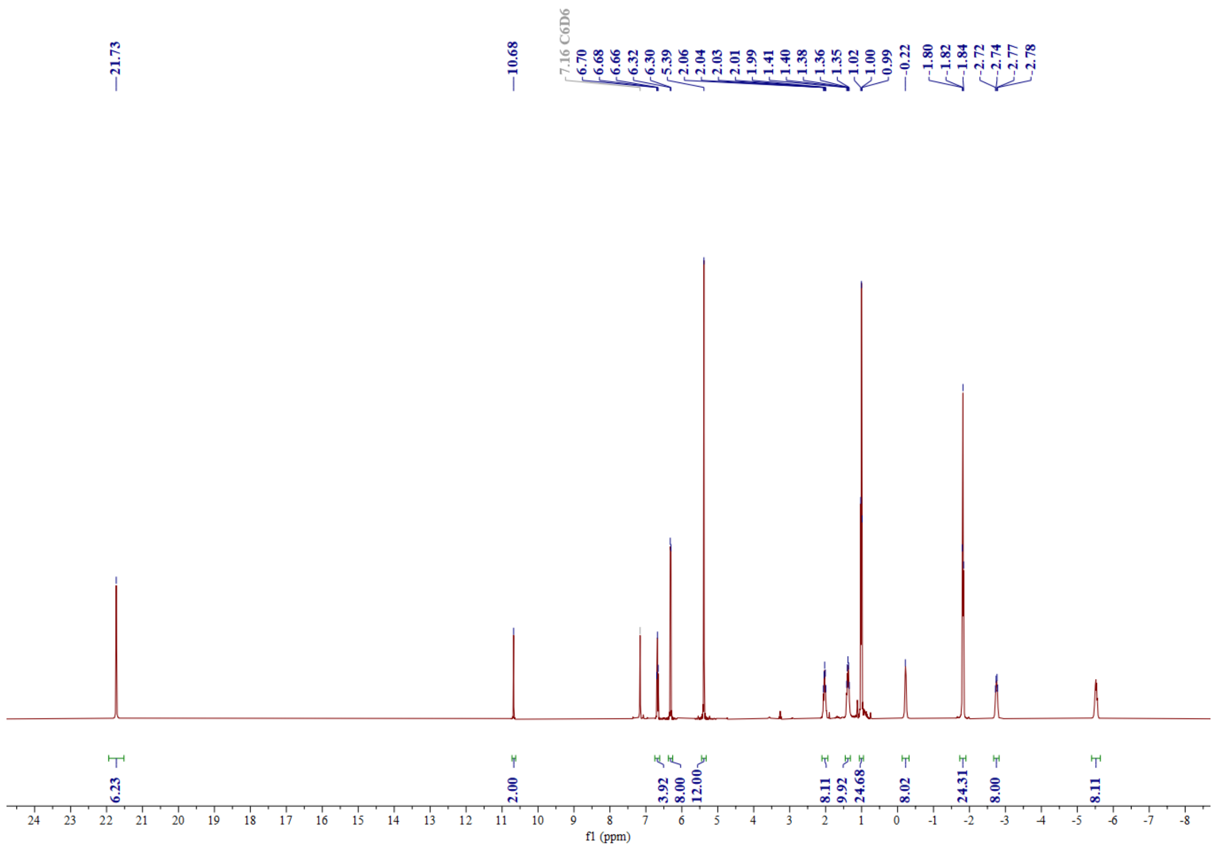

Fig. S6. ^{1}H NMR (400 MHz, C_6D_6, 298K) spectrum of $[(BDI)Sm(THF)]_2(\mu\text{–}\eta^6,\eta^6\text{-}C_6H_6)$ (**2-Sm**), δ, ppm: 21.73 (s, 6H, C*H* of the bound benzene), 10.68 (s, 2H, CC*H*C), 6.68 (t, *J* = 7.8 Hz, 4H, *para*-C*H* of aryl), 6.31 (d, *J* = 8.0 Hz, 8H, *meta*-C*H* of aryl), 5.39 (s, 12H, CCH_3), 2.10–1.95 (m, 8H, CH_2CH$_3$), 1.45–1.31 (m, 8H, CH_2CH$_3$), 1.00 (t, *J* = 7.4 Hz, 24H, CH$_2$CH_3), –0.22 (s, 8H, C*H*(CH$_2$CH$_3$)$_2$), –1.82 (t, *J* = 7.1 Hz, 24H, CH$_2$CH_3), –2.67 to –2.82 (m, 8H, CH_2CH$_3$), –5.40 to –5.64 (m, 8H, CH_2CH$_3$). The peaks of coordinating THF molecules are not observed probably due to the loss of THF molecules under reduced pressure during drying process.

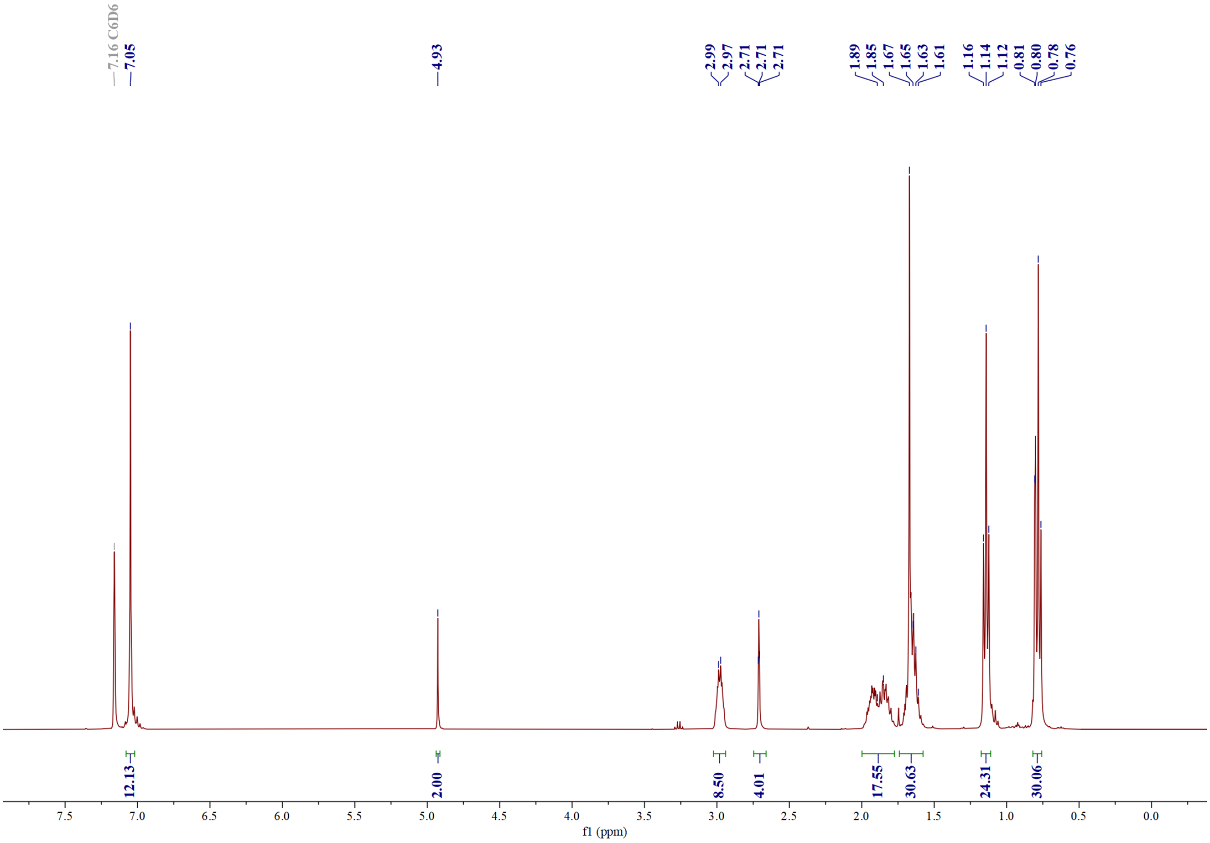

Fig. S7. ^{1}H NMR (400 MHz, C_6D_6, 298K) spectrum of $[(BDI)Y]_2(\mu\text{–}\eta^6,\eta^6\text{-}C_8H_{10})$ (**3-Y**), δ, ppm: 7.05 (s, 12H, C*H* of aryl), 4.93 (s, 2H, CC*H*C), 3.03–2.92 (m, 8H, C*H*$(CH_2CH_3)_2$), 2.71 (t, $J_{Y\text{–}H}$ = 1.7 Hz, 4H, C*H* of the bound *p*-xylene), 2.00–1.77 (m, 16H, CH_2CH_3), 1.74–1.57 (m, 28H, CCH_3 (12H) & CH_2CH_3 (16H)), 1.14 (t, J = 7.3 Hz, 24H, CH_2CH_3), 0.82–0.76 (m, 30H, CH_2CH_3 (24H) & CH_3 of the bound *p*-xylene (6H)).

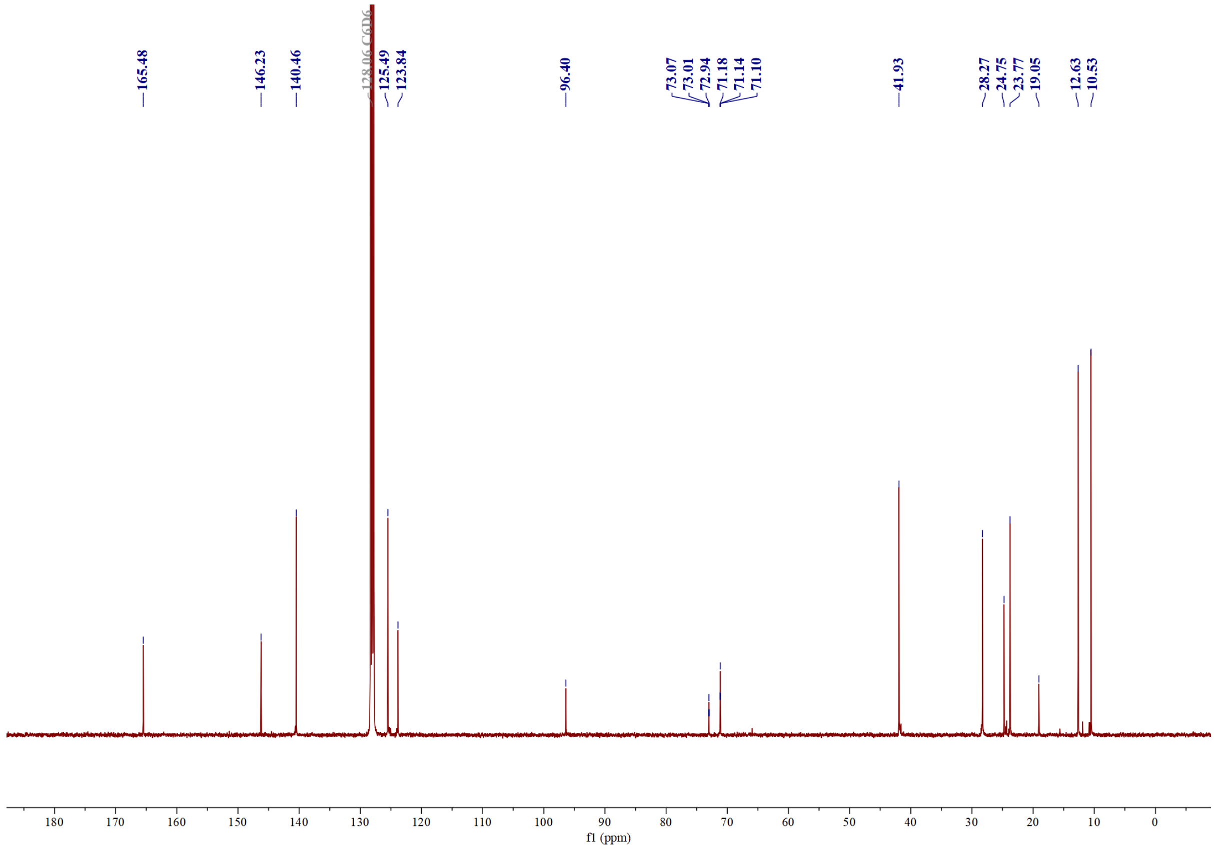

Fig. S8. $^{13}C\{H\}$ NMR (101 MHz, C_6D_6, 298K) spectrum of $[(BDI)Y]_2(\mu\text{–}\eta^6,\eta^6\text{-}C_8H_{10})$ (**3-Y**), δ, ppm: 165.48 (*C*(CH_3)N), 146.23, 140.46, 125.49, 123.84 (*C* of aryl), 96.40 (C*C*HC), 73.01 (*C*Me of the bound xylene, $J_{Y\text{-}C}$ = 6.8 Hz), 71.14 (*C*H of the bound *p*-xylene, $J_{Y\text{-}C}$ = 4.0 Hz), 41.93 (*C*H(CH_2CH_3)$_2$), 28.27 (*C*H_2CH_3), 24.75 (*C*H(CH_2CH_3)$_2$), 23.77 (*C*H_2CH_3), 19.05 (*C*H_3 of the bound *p*-xylene), 12.63 (CH_2*C*H_3), 10.53 (CH_2*C*H_3).

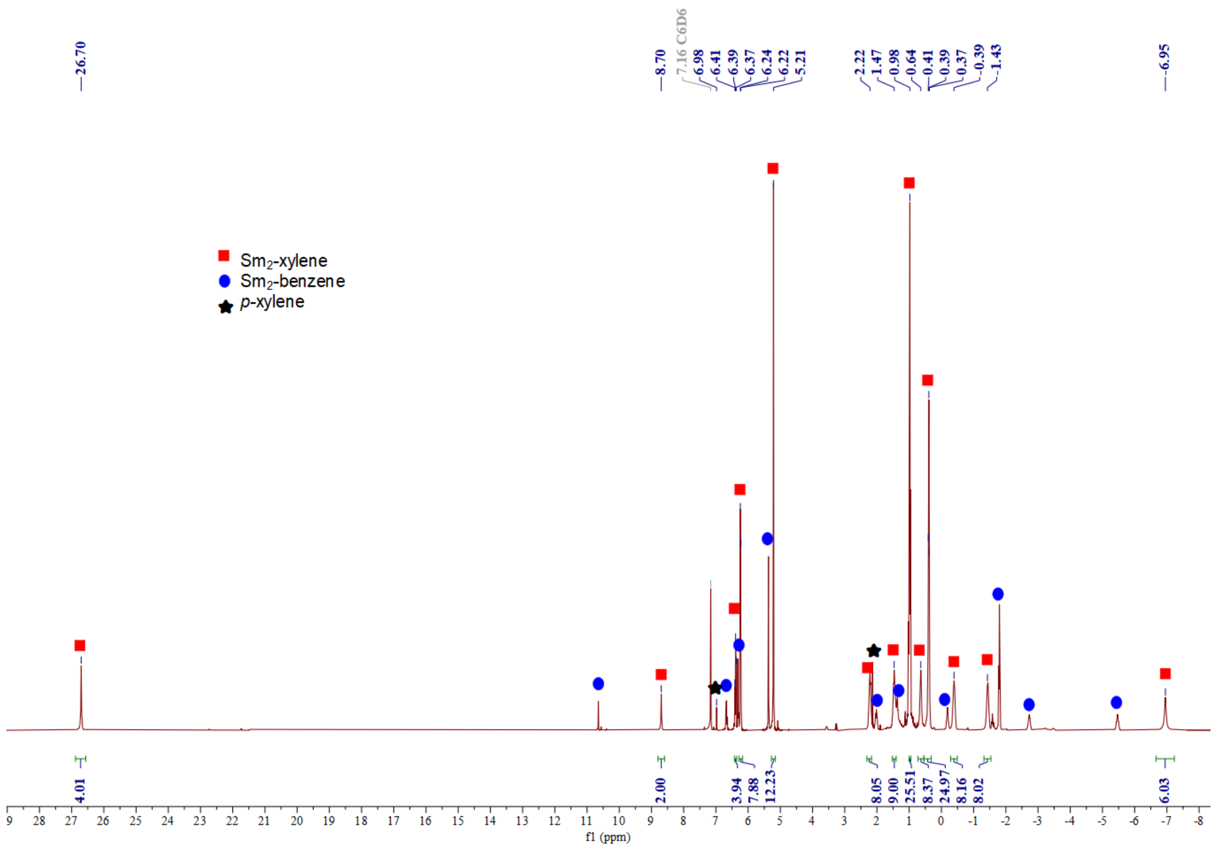

Fig. S9. ^{1}H NMR (400 MHz, 298K) spectrum of [(BDI)Sm(THF)]$_2$(μ–η^6,η^6-C_8H_{10}) (**3-Sm**) in C_6D_6 (5 min after dissolution). 26.70 (s, 4H, C*H* of the bound *p*-xylene), 8.70 (s, 2H, CC*H*C), 6.39 (t, *J* = 7.8 Hz, 4H, *para*-C*H* of aryl), 6.23 (d, *J* = 7.8 Hz, 8H, *meta*-C*H* of aryl), 5.21 (s, 12H, CC*H*$_3$), 2.31–2.15 (m, 8H, C*H*$_2$CH$_3$), 1.54–1.40 (m, 8H, C*H*$_2$CH$_3$), 0.98 (t, *J* = 7.3 Hz, 24H, CH$_2$C*H*$_3$), 0.64 (s, 8H, C*H*(CH$_2$CH$_3$)$_2$), 0.39 (t, *J* = 6.7 Hz, 24H, CH$_2$C*H*$_3$), –0.30 to –0.47(m, 8H, C*H*$_2$CH$_3$), –1.32 to –1.53(m, 8H, C*H*$_2$CH$_3$), –6.95 (s, 6H, C*H*$_3$ of the bound *p*-xylene).

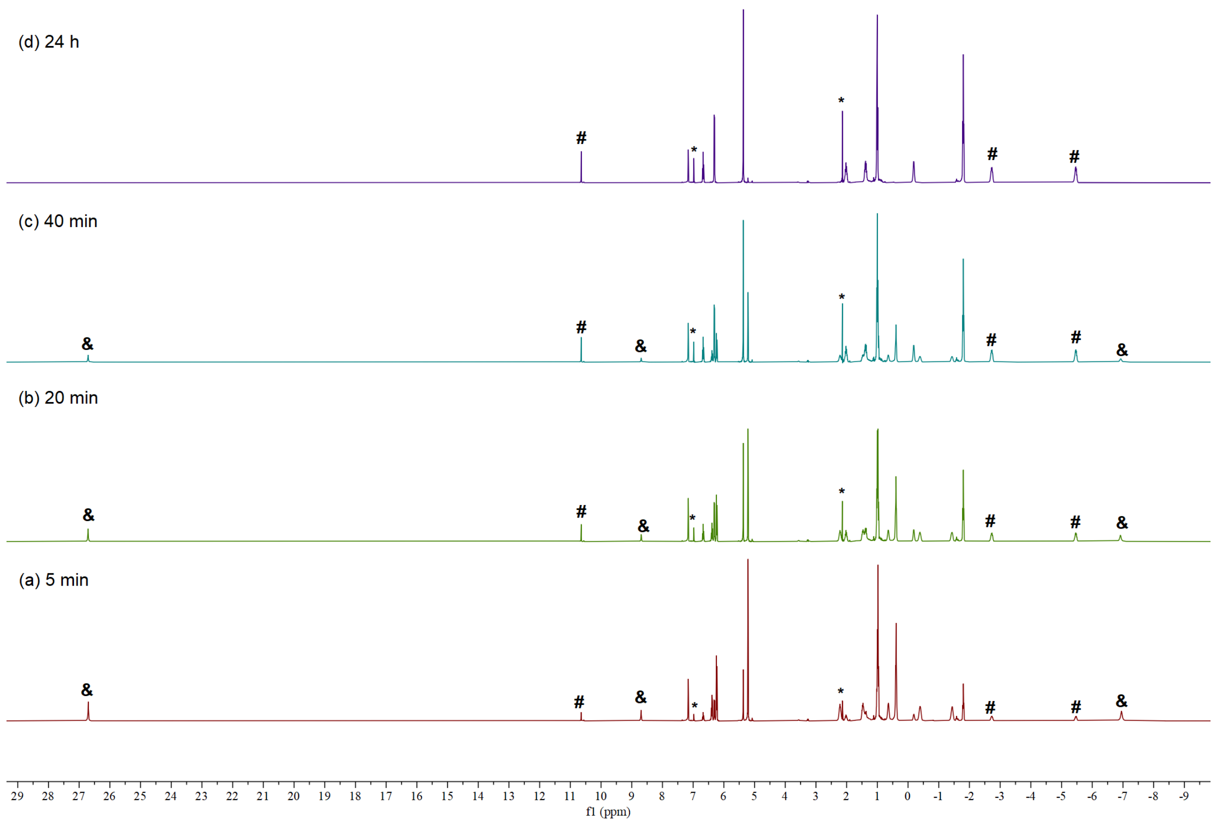

Fig. S10. ^{1}H NMR (400 MHz, C_6D_6, 298K) spectra for monitoring the arene exchange reaction of **3-Sm** (characteristic peaks labelled with "&") in C_6D_6 at 25 °C. #: the characteristic peaks of $[(BDI)Sm(THF)]_2(\mu\text{–}\eta^6,\eta^6\text{-}C_6D_6)$; *: the peaks of free *p*-xylene. From bottom to top: (a) 5 min; (b) 20 min; (c) 40 min; (d) 24 h (full conversion).

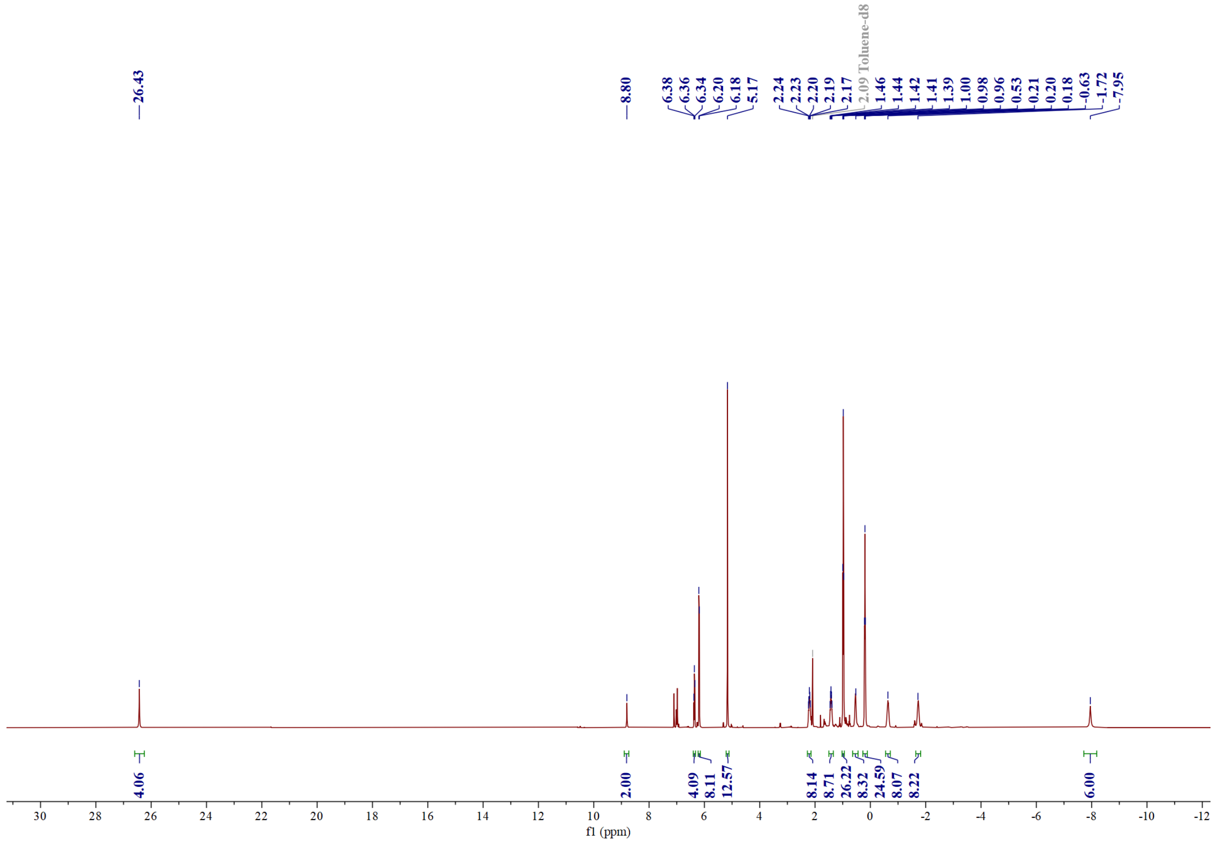

Fig. S11. ^{1}H NMR (400 MHz, 298K) spectrum of $[(BDI)Sm(THF)]_2(\mu\text{–}\eta^6,\eta^6\text{-}C_8H_{10})$ (**3-Sm**) in C_7D_8 (10 min after dissolution). 26.43 (s, 4H, C*H* of the bound *p*-xylene), 8.80 (s, 2H, CC*H*C), 6.36 (t, *J* = 7.7 Hz, 4H, *para*-C*H* of aryl), 6.19 (d, *J* = 7.7 Hz, 8H, *meta*-C*H* of aryl), 5.17 (s, 12H, CC*H*3), 2.28–2.15 (m, 8H, C*H*2CH3), 1.50–1.35 (m, 8H, C*H*2CH3), 0.98 (t, *J* = 7.4 Hz, 24H, CH2C*H*3), 0.53 (s, 8H, C*H*(CH2CH3)2), 0.20 (t, *J* = 6.9 Hz, 24H, CH2C*H*3), –0.54 to –0.72 (m, 8H, C*H*2CH3), –1.64 to –1.82 (m, 8H, C*H*2CH3), –7.95 (s, 6H, C*H*3 of the bound *p*-xylene). The peaks of coordinating THF molecules are not observed probably due to the loss of THF molecules under reduced pressure during drying process.

Note: The arene exchange reaction of **3-Sm** in C_7D_8 was slower than that in C_6D_6. Only a small amount of $[(BDI)Sm(THF)]_2(\mu\text{–}\eta^6,\eta^6\text{-}C_7D_8)$ was observed after 50 min at room temperature.

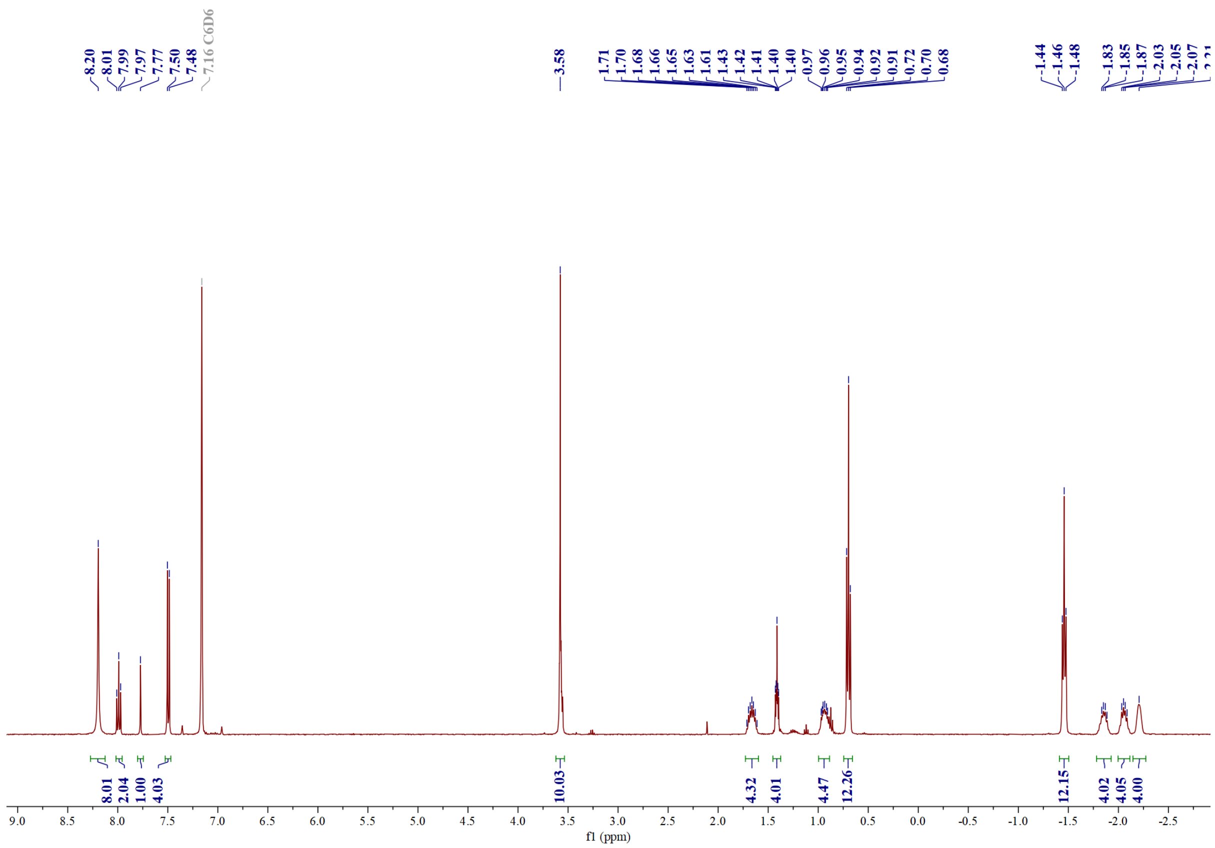

Fig. S12. ^{1}H NMR (400 MHz, C_6D_6, 298K) spectrum of (BDI)Sm(η^8-COT) (**4-Sm**), δ, ppm: 8.20 (s, 8H, C*H* of COT), 7.99 (t, *J* = 7.8 Hz, 2H, *para*-C*H* of aryl), 7.77 (s, 1H, CC*H*C), 7.49 (d, *J* = 7.8 Hz, 4H, *meta*-C*H* of aryl), 3.58 (s, 10H, CCH_3 & OCH_2 of free THF), 1.72–1.59 (m, 4H, CH_2CH$_3$), 1.45–1.37 (m, 4H, OCH$_2$CH_2 of free THF), 1.00–0.89 (m, 4H, CH_2CH$_3$), 0.70 (t, *J* = 7.4 Hz, 12H, CH$_2$CH_3), –1.46 (t, *J* = 7.2 Hz, 12H, CH$_2$CH_3), –1.78 to –1.92 (m, 4H, CH_2CH$_3$), –1.99 to –2.11 (m, 4H, CH_2CH$_3$), –2.21 (s, 4H, C*H*(CH$_2$CH$_3$)$_2$).

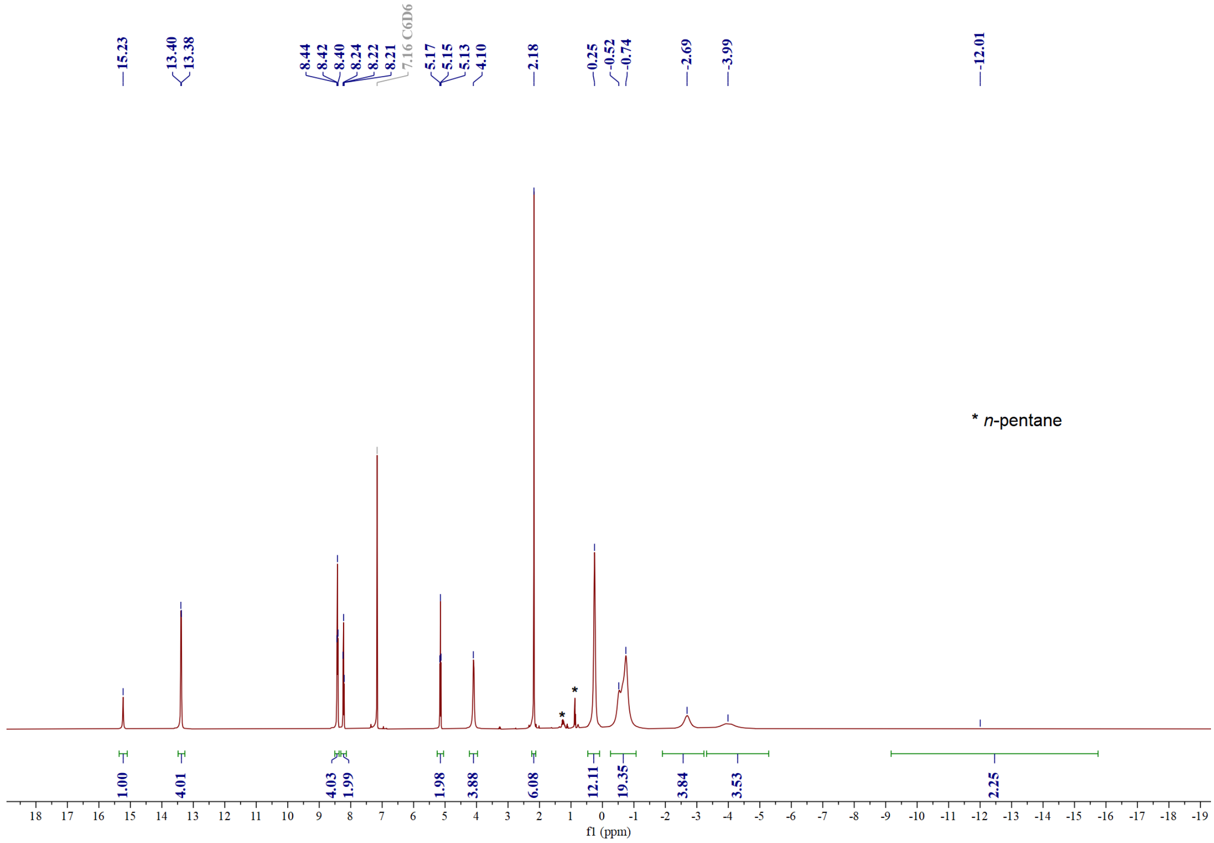

Fig. S13. ^{1}H NMR (400 MHz, C_6D_6, 298K) spectrum of (BDI)Sm(η[4]-C_4Ph_2) (**5-Sm**), δ, ppm: 15.23 (s, 1H, CC*H*C), 13.39 (d, *J* = 7.4 Hz, 4H), 8.42 (t, *J* = 7.0 Hz, 4H, *meta*-C*H* of C_4Ph_2), 8.22 (t, *J* = 7.2 Hz, 2H), 5.15 (t, *J* = 7.6 Hz, 2H), 4.10 (s, 4H), 2.18 (s, 6H, CC*H*3), 0.25 (s, 12H, CH_2CH_3), –0.26 to –1.07 (m, 20H, CH_2CH_3 (12H) & CH_2CH_3 (8H)), –2.69 (s, 4H), –3.99 (s, 4H), –12.01 (br s, 4H). The signals cannot be assigned completely because of the signal broadening and similar signal splitting in aryl groups of ligand backbone and C_4Ph_4.

S3. X-ray Crystallography

S3.1. General

All structures were solved by the intrinsic phasing method with SHELXT[5] and refined by full-matrix least-squares procedures utilizing SHELXL[6, 7] within Olex2 crystallographic software package.[8] The PLATON[9] routine SQUEEZE[10] was used in the refinement of some structures.

S3.2. CCDC Deposition

All crystal structures reported in this work have been deposited to the Cambridge Crystallographic Data Center (CCDC) with the following deposition numbers:

CCDC-2345765: (BDI)YI_2(THF) (**1-Y**)

CCDC-2345766: (BDI)SmI_2(THF) (**1-Sm**)

CCDC-2345769: $[(BDI)Y]_2(\mu\text{–}\eta^6,\eta^6\text{-}C_6H_6)$ (**2-Y**)

CCDC-2345764: $[(BDI)Sm(THF)]_2(\mu\text{–}\eta^6,\eta^6\text{-}C_6H_6)$ (**2-Sm**)

CCDC-2345768: $[(BDI)Y]_2(\mu\text{–}\eta^6,\eta^6\text{-}C_8H_{10})$ (**3-Y**)

CCDC-2345762: $[(BDI)Sm(THF)]_2(\mu\text{–}\eta^6,\eta^6\text{-}C_8H_{10})$ (**3-Sm**)

CCDC-2345767: (BDI)Sm(η^8-COT) (**4-Sm**)

CCDC-2345763: (BDI)Sm(η^4-C_4Ph_2) (**5-Sm**)

(BDI)YI_2(THF) (1-Y)

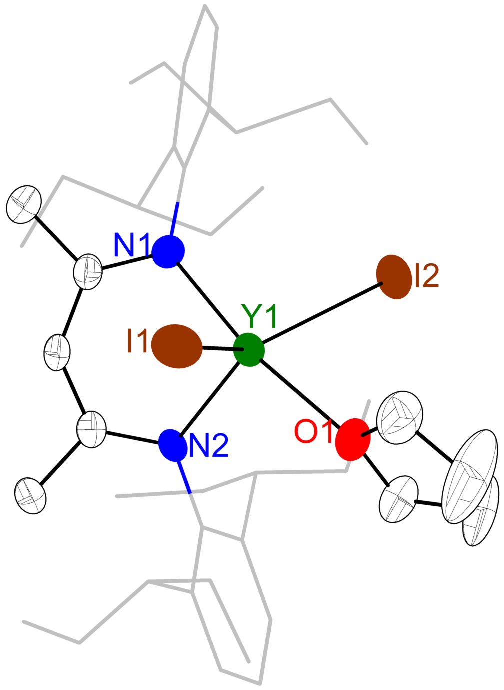

Fig. S14. Representation of (BDI)YI_2(THF) (**1-Y**) with thermal ellipsoids set at 50% probability. Hydrogen atoms and disordered counterpart (THF) were omitted for clarity. Sections of aryl substituents were shown as wireframe for clarity. Selected distances (Å) and angles (°): Y1–N1 2.2760(48), Y1–N2 2.2681(48), Y1–I1 2.9239(7), Y1–I2 2.9469(6), Y1–O1 2.3725(40), N1–Y1–N2 87.736(170), I1–Y1–I2 136.217(21).

Single crystals suitable for X-ray crystallography were grown from a hexanes solution at –35 °C. A total of 24757 reflections (–17 ≤ h ≤ 16, –30 ≤ k ≤ 26, –21 ≤ l ≤ 13) were collected at T = 180.00 K with $2\theta_{max}$ = 58.984°, of which 10611 were unique. The residual peak and hole electron density were 1.72 and –1.22 e$Å^{-3}$. The least-squares refinement converged normally with residuals of R_1 = 0.0662 and GOF = 1.077. Crystal and refinement data for **1-Y**: formula $C_{41}H_{65}I_2N_2OY$, space group $P2_1/n$, a = 12.5793(4) Å, b = 21.9106(6) Å, c = 15.8022(4) Å, β = 99.436(3)°, V = 4296.5(2) $Å^3$, Z = 4, μ = 2.825 mm^{-1}, F(000) = 1912.0, R_1 = 0.1008 and wR_2 = 0.1970 (based on all data).

(BDI)SmI$_2$(THF) (1-Sm)

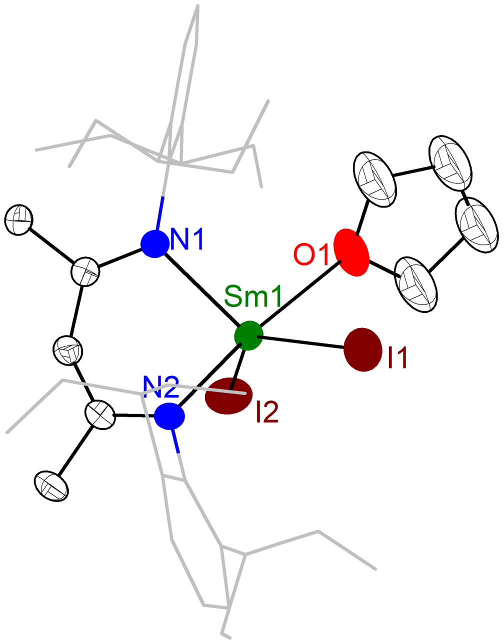

Fig. S15. Representation of (BDI)SmI$_2$(THF) (**1-Sm**) with thermal ellipsoids set at 50% probability. Hydrogen atoms were omitted for clarity. Sections of aryl substituents were shown as wireframe for clarity. Selected distances (Å) and angles (°): Sm1–N1 2.3088(27), Sm1–N2 2.3133(35), Sm1–I1 3.0166(5), Sm1–I2 2.9896(5), Sm1–O1 2.4409(37), N1–Sm1–N2 86.557(109), I1–Sm1–I2 137.905(14).

Single crystals suitable for X-ray crystallography were grown from a Et_2O solution at room temperature. A total of 32901 reflections ($-16 \le h \le 17$, $-29 \le k \le 30$, $-21 \le l \le 22$) were collected at T = 180.00 K with $2\theta_{max}$ = 59.032°, of which 10887 were unique. The residual peak and hole electron density were 1.60 and –1.53 eÅ^{-3}. The least-squares refinement converged normally with residuals of R_1 = 0.0387 and GOF = 1.061. Crystal and refinement data for **1-Sm**: formula $C_{41}H_{65}I_2N_2OSm$, space group $P2_1/n$, a = 12.5729(3) Å, b = 21.8852(4) Å, c = 15.9583(3) Å, β = 99.806(2)°, V = 4326.94(16) Å^3, Z = 4, μ = 2.814 mm^{-1}, F(000) = 2004.0, R_1 = 0.0480 and wR_2 = 0.1038 (based on all data).

[(BDI)Y]$_2$(μ–η^6,η^6-C_6H_6) (2-Y)

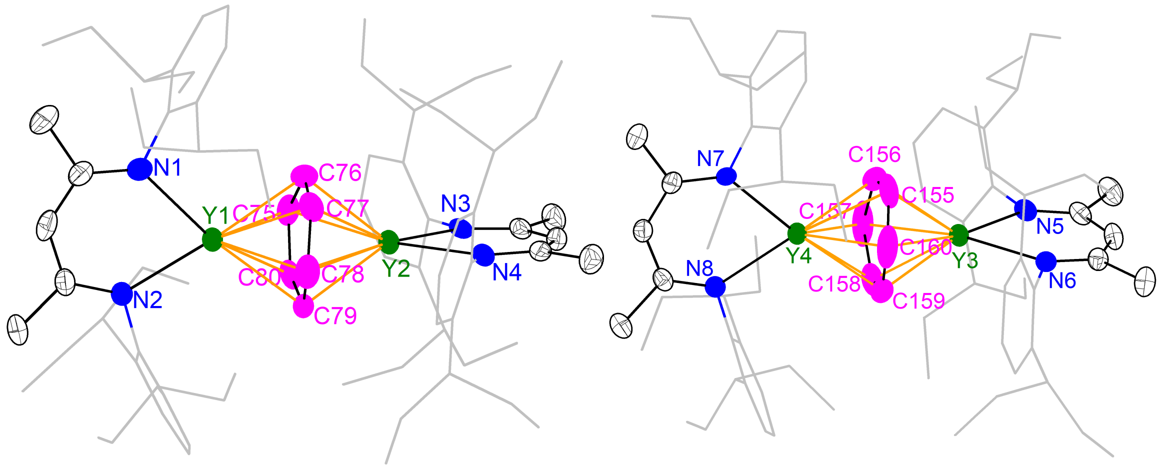

Fig. S16. Representation of [(BDI)Y]$_2$(μ–η^6,η^6-C_6H_6) (**2-Y**) with thermal ellipsoids set at 50% probability. Hydrogen atoms were omitted for clarity. Sections of aryl substituents were shown as wireframe for clarity. Both crystallographically independent molecules in an unsymmetric unit were shown. Selected distances (Å) and angles (°): For independent molecule 1: Y1–N1 2.3848(32), Y1–N2 2.3547(49), Y2–N3 2.3913(40), Y2–N4 2.3748(40), Y1–Y2 3.9984(8), Y1–C75 2.4106(54), Y1–C76 2.5190(65), Y1–C77 2.4780(59), Y1–C78 2.4196(49), Y1–C79 2.5339(40), Y1–C80 2.4673(44), Y2–C75 2.5195(48), Y2–C76 2.4138(43), Y2–C77 2.4643(44), Y2–C78 2.5268(56), Y2–C79 2.4106(61), Y2–C80 2.4645(59), Y1–R_{cent} 2.0022(6), Y2–R_{cent} 1.9962(6), C75–C76 1.4635(77), C76–C77 1.4364(79), C77–C78 1.4493(72), C78–C79 1.4647(73), C79–C80 1.4524(78), C80–C75 1.4440(69), N1–Y1–N2 76.330(151), N3–Y2–N4 75.207(143), Y1–R_{cent}–Y2 179.585(25). Torsion angles: C77–C78/C79–C80 –13.1(8), C75–C76/C78–C79 10.8(5). For independent molecule 2: Y3–N5 2.3715(33), Y3–N6 2.3859(43), Y4–N7 2.3909(33), Y4–N8 2.3681(33), Y3–Y4 3.9997(6), Y3–C155 2.4020(48), Y3–C156 2.4537(57), Y3–C157 2.5247(59), Y3–C158 2.4027(47), Y3–C159 2.4543(47), Y3–C160 2.5221(43), Y4–C155 2.4987(47), Y4–C156 2.4481(60), Y4–C157 2.3928(44), Y4–C158 2.5108(48), Y4–C159 2.4769(57), Y4–C160 2.4161(57), Y3–R_{cent} 2.0017(4), Y4–R_{cent} 1.9982(4), C155–C156 1.4247(117), C156–C157 1.4467(94), C157–C158 1.4480(95), C158–C159 1.4057(104), C159–C160 1.4312(92), C160–C155 1.4415(94), N5–Y3–N6 74.948(135), N7–Y4–N8 75.688(129), Y3–R_{cent}–Y4 178.907(21). Torsion angles: C156–C157/C158–C159 12.7(9), C157–C158/C160–C155 –10.9(5).

Single crystals suitable for X-ray crystallography were grown from a *n*-pentane solution at –35 °C. A total of 189297 reflections (–38 ≤ h ≤ 40, –30 ≤ k ≤ 27, –34 ≤ l ≤ 35) were collected at T = 179.99(10) K with $2\theta_{max}$ = 59.1°, of which 38188 were unique. The residual peak and hole electron density were 1.99 and –1.23 eÅ^{-3}. The least-squares refinement converged normally with residuals of R_1 = 0.0833 and GOF = 1.033. Crystal and refinement data for **2-Y**: formula $C_{80}H_{120}N_4Y_2$, space group $P2_1/c$, a = 28.8724(5) Å, b = 21.9655(2) Å, c = 25.9782(4) Å, β = 116.683(2)°, V = 14720.7(4) Å^3, Z = 8, μ = 1.613 mm^{-1}, F(000) = 5648.0, R_1 = 0.1634 and wR_2 = 0.1870 (based on all data).

[(BDI)Sm(THF)]$_2$(μ–η^6,η^6-C_6H_6) (2-Sm)

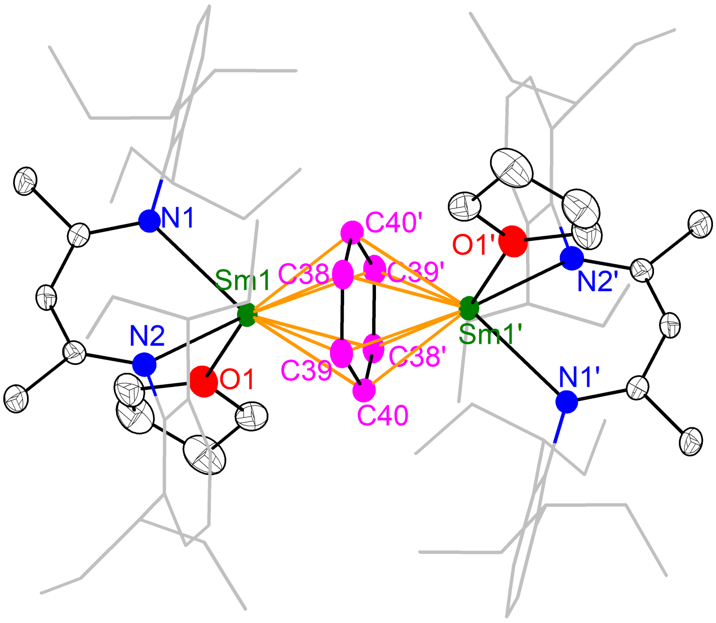

Fig. S17. Representation of [(BDI)Sm(THF)]$_2$(μ–η^6,η^6-C_6H_6) (**2-Sm**) with thermal ellipsoids set at 50% probability. Hydrogen atoms and disordered counterpart (THF) were omitted for clarity. Sections of aryl substituents were shown as wireframe for clarity. Selected distances (Å) and angles (°): Sm1–N1 2.5099(15), Sm1–N2 2.5096(18), Sm1–Sm1′ 4.1906(4), Sm1–C38 2.5590(15), Sm1–C39 2.4927(13), Sm1–C40 2.6025(17), Sm1–C38′ 2.5335(19), Sm1–C39′ 2.5979(22), Sm1–C40′ 2.6025(17), Sm1–R_{cent} 2.0953(3), C38–C39 1.4533(34), C39–C40 1.4501(27), C40–C38′ 1.4547(28), N1–Sm1–N2 76.436(47), Sm1–R_{cent}–Sm1′ 180.000(11). Torsion angles: C40′–C38/C39–C40 12.8(3), C38–C39/C38′–C39′ 0(2).

Single crystals suitable for X-ray crystallography were grown from a *n*-pentane solution containing a few drops of THF at –35 °C. A total of 27011 reflections (–10 ≤ h ≤ 17, –18 ≤ k ≤ 16, –21 ≤ l ≤ 20) were collected at T = 179.99(10) K with $2\theta_{max}$ = 59.192°, of which 10250 were unique. The residual peak and hole electron density were 0.64 and –0.43 eÅ^{-3}. The least-squares refinement converged normally with residuals of R_1 = 0.0219 and GOF = 1.055. Crystal and refinement data for **2-Sm**: formula $C_{88}H_{136}N_4O_2Sm_2$, space group *P*–1, *a* = 12.8287(2) Å, *b* = 13.2492(2) Å, *c* = 15.2084(2) Å, *α* = 110.2990(10)°, *β* = 94.3910(10)°, *γ* = 118.829(2)°, *V* = 2028.26(6) Å^3, *Z* = 1, *μ* = 1.481 mm^{-1}, *F*(000) = 832.0, R_1 = 0.0246 and wR_2 = 0.0558 (based on all data).

$[(BDI)Y]_2(\mu\text{–}\eta^6,\eta^6\text{-}C_8H_{10})$ (3-Y)

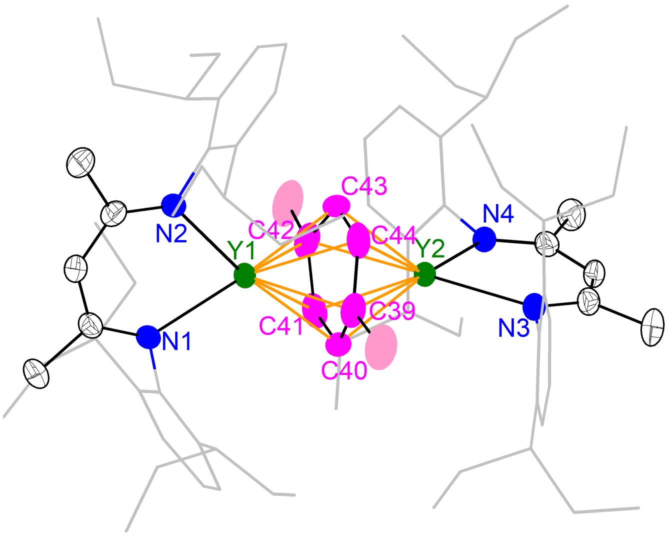

Fig. S18. Representation of $[(BDI)Y]_2(\mu\text{–}\eta^6,\eta^6\text{-}C_8H_{10})$ (**3-Y**) with thermal ellipsoids set at 50% probability. Hydrogen atoms were omitted for clarity. Sections of aryl substituents were shown as wireframe for clarity. Selected distances (Å) and angles (°): Y1–N1 2.3776(25), Y1–N2 2.3919(26), Y2–N3 2.4083(28), Y2–N4 2.4137(25), Y1–Y2 4.0210(7), Y1–C39 2.4128(34), Y1–C40 2.5141(36), Y1–C41 2.4984(33), Y1–C42 2.3934(33), Y1–C43 2.5003(38), Y1–C44 2.5007(40), Y2–C39 2.5950(32), Y2–C40 2.4361(37), Y2–C41 2.4410(39), Y2–C42 2.5696(36), Y2–C43 2.4318(37), Y2–C44 2.4537(34), Y1–R_{cent} 1.9993(5), Y2–R_{cent} 2.0220(5), C39–C40 1.4388(52), C40–C41 1.4584(53), C41–C42 1.4662(55), C42–C43 1.4619(55), C43–C44 1.4451(62), C44–C39 1.4572(56), N1–Y1–N2 77.009(86), N3–Y2–N4 76.222(88), Y1–R_{cent}–Y2 178.704(18). Torsion angles: C44–C39/C40–C41 –14.2(6), C42–C43/C39–C40 12.4(3).

Single crystals suitable for X-ray crystallography were grown from a *n*-pentane solution at –35 °C. A total of 44814 reflections ($-30 \leq h \leq 21$, $-15 \leq k \leq 18$, $-32 \leq l \leq 35$) were collected at T = 180.00 K with $2\theta_{max}$ = 59.032°, of which 18550 were unique. The residual peak and hole electron density were 1.43 and –0.88 eÅ^{-3}. The least-squares refinement converged normally with residuals of R_1 = 0.0565 and GOF = 1.047. Crystal and refinement data for **3-Y**: formula $C_{82}H_{124}N_4Y_2$, space group $P2_1/c$, a = 22.0209(6) Å, b = 13.3172(3) Å, c = 25.7853(6) Å, β = 96.038(3)°, V = 7519.8(3) Å^3, Z = 4, μ = 1.580 mm^{-1}, $F(000)$ = 2888.0, R_1 = 0.0895 and wR_2 = 0.1649 (based on all data).

[(BDI)Sm(THF)]$_2$(μ–η^6,η^6-C_8H_{10}) (3-Sm)

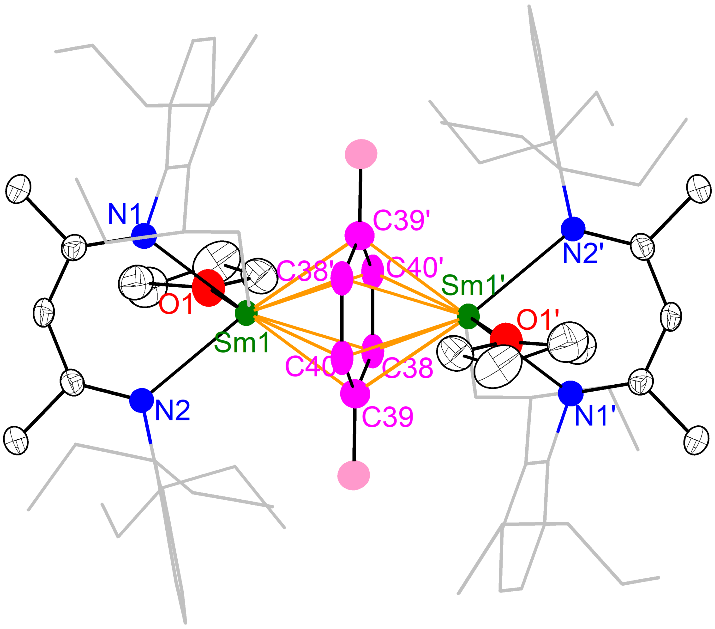

Fig. S19. Representation of [(BDI)Sm(THF)]$_2$(μ–η^6,η^6-C_8H_{10}) (**3-Sm**) with thermal ellipsoids set at 50% probability. Hydrogen atoms and disordered counterpart (THF) were omitted for clarity. Sections of aryl substituents were shown as wireframe for clarity. Selected distances (Å) and angles (°): Sm1–N1 2.5242(17), Sm1–N2 2.5406(18), Sm1–Sm1′ 4.2182(5), Sm1–C38 2.5799(20), Sm1–C39 2.5440(23), Sm1–C40 2.5770(31), Sm1–C38′ 2.5262(31), Sm1–C39′ 2.5978(26), Sm1–C40′ 2.5945(22), Sm1–R_{cent} 2.1091(3), C38–C39 1.4499(42), C39–C40 1.4825(43), C40–C38′ 1.4752(27), N1–Sm1–N2 75.915(65), Sm1–R_{cent}–Sm1′ 180.000. Torsion angles: C40–C38′/C39′–C40′ 5.1(4), C38–C39/C38′–C39′ 0(3).

Single crystals suitable for X-ray crystallography were grown from a *n*-pentane solution containing a few drops of THF at –35 °C. A total of 26322 reflections (–17 ≤ h ≤ 16, –18 ≤ k ≤ 14, –21 ≤ l ≤ 20) were collected at T = 179.99(10) K with $2\theta_{max}$ = 58.7°, of which 10253 were unique. The residual peak and hole electron density were 1.06 and –0.75 eÅ^{-3}. The least-squares refinement converged normally with residuals of R_1 = 0.0304 and GOF = 1.082. Crystal and refinement data for **3-Sm**: formula $C_{90}H_{140}N_4O_2Sm_2$, space group *P*–1, *a* = 12.7862(7) Å, *b* = 13.2072(6) Å, *c* = 15.4523(6) Å, *α* = 64.913(4)°, *β* = 70.485(4)°, *γ* = 61.214(5)°, *V* = 2043.3(2) Å^3, *Z* = 1, *μ* = 1.471 mm^{-1}, *F*(000) = 848.0, R_1 = 0.0381 and wR_2 = 0.0775 (based on all data).

(BDI)Sm(η⁸-COT) (4-Sm)

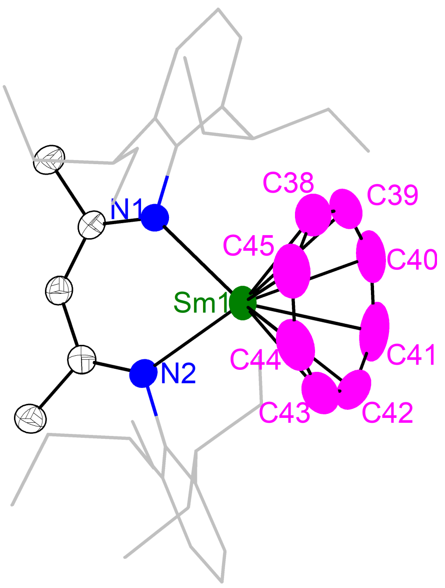

Fig. S20. Representation of (BDI)Sm(η⁸-COT) (**4-Sm**) with thermal ellipsoids set at 50% probability. Hydrogen atoms were omitted for clarity. Sections of aryl substituents were shown as wireframe for clarity. Selected distances (Å) and angles (°): Sm1–N1 2.4606(20), Sm1–N2 2.4280(17), Sm1–C38 2.6342(39), Sm1–C39 2.6366(38), Sm1–C40 2.6239(33), Sm1–C41 2.6207(37), Sm1–C42 2.6332(40), Sm1–C43 2.6274(34), Sm1–C44 2.6257(31), Sm1–C45 2.6271(36), Sm1–R_{cent} 1.8948(3), C38–C39 1.3780(52), C39–C40 1.392(7), C40–C41 1.4121(69), C41–C42 1.4289(61), C42–C43 1.4201(65), C43–C44 1.3538(71), C44–C45 1.3862(61), C45–C38 1.3847(51), N1–Sm1–N2 73.408(59).

Single crystals suitable for X-ray crystallography were grown from a Et_2O solution at –35 °C. A total of 32606 reflections (–19 ≤ h ≤ 19, –20 ≤ k ≤ 24, –21 ≤ l ≤ 21) were collected at T = 180.00 K with $2\theta_{max}$ = 58.992°, of which 10231 were unique. The residual peak and hole electron density were 0.89 and –0.72 eÅ^{-3}. The least-squares refinement converged normally with residuals of R_1 = 0.0321 and GOF = 1.055. Crystal and refinement data for **4-Sm**: formula $C_{45}H_{65}N_2Sm$, space group $P2_1$/n, a = 14.4271(4) Å, b = 17.8919(4) Å, c = 16.0725(4) Å, β = 102.800(3)°, V = 4045.67(18) Å^3, Z = 4, μ = 1.483 mm^{-1}, F(000) = 1644.0, R_1 = 0.0437 and wR_2 = 0.0838 (based on all data).

(BDI)Sm(η^4-C_4Ph_2) (5-Sm)

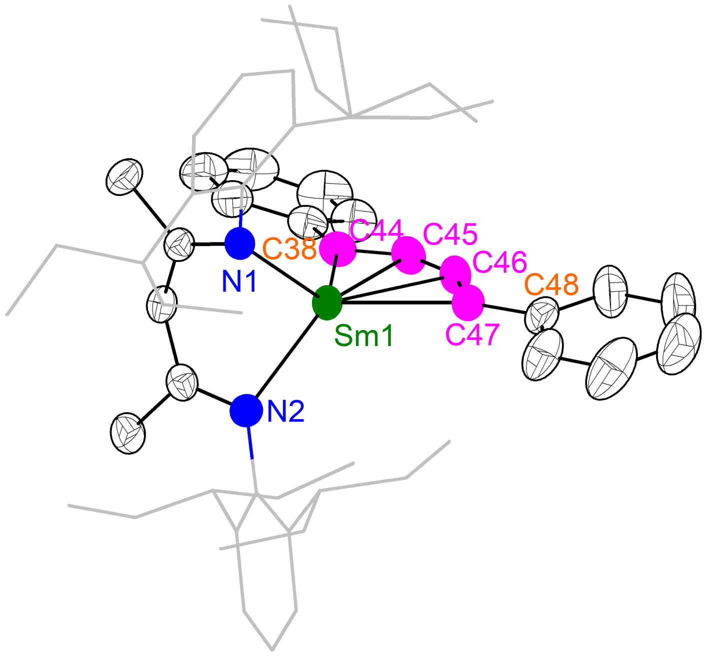

Fig. S21. Representation of (BDI)Sm(η^4-C_4Ph_2) (**5-Sm**) with thermal ellipsoids set at 50% probability. Hydrogen atoms were omitted for clarity. Sections of aryl substituents were shown as wireframe for clarity. Selected distances (Å) and angles (°): Sm1–N1 2.3875(22), Sm1–N2 2.3780(23), Sm1–C44 2.4487(26), Sm1–C45 2.4459(26), Sm1–C46 2.4467(27), Sm1–C47 2.4914(30), C44–C45 1.3195(41), C45–C46 1.3097(40), C46–C47 1.3063(41), C38–C44 1.4670(41), C47–C48 1.4761(40), N1–Sm1–N2 84.043(72), C44–C45–C46 148.978(304), C47–C46–C45 150.998(298), C44–Sm1–C47 92.994(103).

Single crystals suitable for X-ray crystallography were grown from a *n*-pentane solution at –35 °C. A total of 31410 reflections ($-14 \leq h \leq 15$, $-15 \leq k \leq 15$, $-24 \leq l \leq 25$) were collected at T = 180.00 K with $2\theta_{max}$ = 61.432°, of which 12197 were unique. The residual peak and hole electron density were 1.39 and –1.32 eÅ^{-3}. The least-squares refinement converged normally with residuals of R_1 = 0.0412 and GOF = 1.125. Crystal and refinement data for **5-Sm**: formula $C_{53}H_{67}N_2Sm$, space group *P*–1, *a* = 10.8737(4) Å, *b* = 11.7593(5) Å, *c* = 18.5401(8) Å, α = 98.600(4)°, β = 93.568(3)°, γ = 92.844(3)°, *V* = 2335.30(17) Å^3, *Z* = 2, μ = 1.292 mm^{-1}, *F*(000) = 922.0, R_1 = 0.0486 and wR_2 = 0.1071 (based on all data).

Table. S1. Selected distances (Å) and angles (°) of **2-Y** and **3-Y**.

	**2-Y**[a]	**2-Y**[b]		**3-Y**
Y1(3)–N1(5)	2.3848(32)	2.3715(33)	Y1–N1	2.3776(25)
Y1(3)–N2(6)	2.3547(49)	2.3859(43)	Y1–N2	2.3919(26)
Y2(4)–N3(7)	2.3913(40)	2.3909(33)	Y2–N3	2.4083(28)
Y2(4)–N4(8)	2.3748(40)	2.3681(33)	Y2–N4	2.4137(25)
Y1(3)–Y2(4)	3.9984(8)	3.9997(6)	Y1–Y2	4.0210(7)
Y1(3)–C75(155)	2.4106(54)	2.4020(48)	Y1–C39	2.4128(34)
Y1(3)–C76(156)	2.5190(65)	2.4537(57)	Y1–C40	2.5141(36)
Y1(3)–C77(157)	2.4780(59)	2.5247(59)	Y1–C41	2.4984(33)
Y1(3)–C78(158)	2.4196(49)	2.4027(47)	Y1–C42	2.3934(33)
Y1(3)–C79(159)	2.5339(40)	2.4543(47)	Y1–C43	2.5003(38)
Y1(3)–C80(160)	2.4673(44)	2.5221(43)	Y1–C44	2.5007(40)
Y2(4)–C75(155)	2.5195(48)	2.4987(47)	Y2–C39	2.5950(32)
Y2(4)–C76(156)	2.4138(43)	2.4481(60)	Y2–C40	2.4361(37)
Y2(4)–C77(157)	2.4643(44)	2.3928(44)	Y2–C41	2.4410(39)
Y2(4)–C78(158)	2.5268(56)	2.5108(48)	Y2–C42	2.5696(36)
Y2(4)–C79(159)	2.4106(61)	2.4769(57)	Y2–C43	2.4318(37)
Y2(4)–C80(160)	2.4645(59)	2.4161(57)	Y2–C44	2.4537(34)
Y1(3)–R_{cent}	2.0022(6)	2.0017(4)	Y1–R_{cent}	1.9993(5)
Y2(4)–R_{cent}	1.9962(6)	1.9982(4)	Y2–R_{cent}	2.0220(5)
C75(155)–C76(156)	1.4635(77)	1.4247(117)	C39–C40	1.4388(52)
C76(156)–C77(157)	1.4364(79)	1.4467(94)	C40–C41	1.4584(53)
C77(157)–C78(158)	1.4493(72)	1.4480(95)	C41–C42	1.4662(55)
C78(158)–C79(159)	1.4647(73)	1.4057(104)	C42–C43	1.4619(55)

C79(159)–C80(160)	1.4524(78)	1.4312(92)	C43–C44	1.4451(62)
C80(160)–C75(155)	1.4440(69)	1.4415(94)	C44–C39	1.4572(56)
N1(5)–Y1(3)–N2(6)	76.330(151)	74.948(135)	N1–Y1–N2	77.009(86)
N3(7)–Y2(4)–N4(8)	75.207(143)	75.688(129)	N3–Y2–N4	76.222(88)
Y1(3)–R_{cent}–Y2(4)	179.585(25)	178.907(21)	Y1–R_{cent}–Y2	178.704(18)
Torsion angles	C77–C78/ C79–C80 –13.1(8) C75–C76/ C78–C79 10.8(5)	C44–C39/ C40–C41 –14.2(6) C42–C43/ C39–C40 12.4(3)	Torsion angles	C44–C39/ C40–C41 –14.2(6) C42–C43/ C39–C40 12.4(3)

[a] independent molecule 1; [b] independent molecule 2.

Table. S2. Selected distances (Å) and angles (°) of **2-Sm** and **3-Sm**.

	**2-Sm**		**3-Sm**
Sm1–N1	2.5099(15)	Sm1–N1	2.5242(17)
Sm1–N2	2.5096(18)	Sm1–N2	2.5406(18)
Sm1–Sm1′	4.1906(4)	Sm1–Sm1′	4.2182(5)
Sm1–C38	2.5590(15)	Sm1–C38	2.5799(20)
Sm1–C39	2.4927(13)	Sm1–C39	2.5440(23)
Sm1–C40	2.6025(17)	Sm1–C40	2.5770(31)
Sm1–C38′	2.5335(19)	Sm1–C38′	2.5262(31)
Sm1–C39′	2.5979(22)	Sm1–C39′	2.5978(26)
Sm1–C40′	2.6025(17)	Sm1–C40′	2.5945(22)
Sm1–R_{cent}	2.0953(3)	Sm1–R_{cent}	2.1091(3)
C38–C39	1.4533(34)	C38–C39	1.4499(42)
C39–C40	1.4501(27)	C39–C40	1.4825(43)
C40–C38′	1.4547(28)	C40–C38′	1.4752(27)
N1–Sm1–N2	76.436(47)	N1–Sm1–N2	75.915(65)
Sm1–R_{cent}–Sm1′	180.000(11)	Sm1–R_{cent}–Sm1′	180.000
Torsion angles	C40′–C38/C39–C40 12.8(3) C38–C39/C38′–C39′ 0(2)	Torsion angles	C40–C38′/C39′–C40′ 5.1(4) C38–C39/C38′–C39′ 0(3)

S4. UV–Vis–NIR Spectra

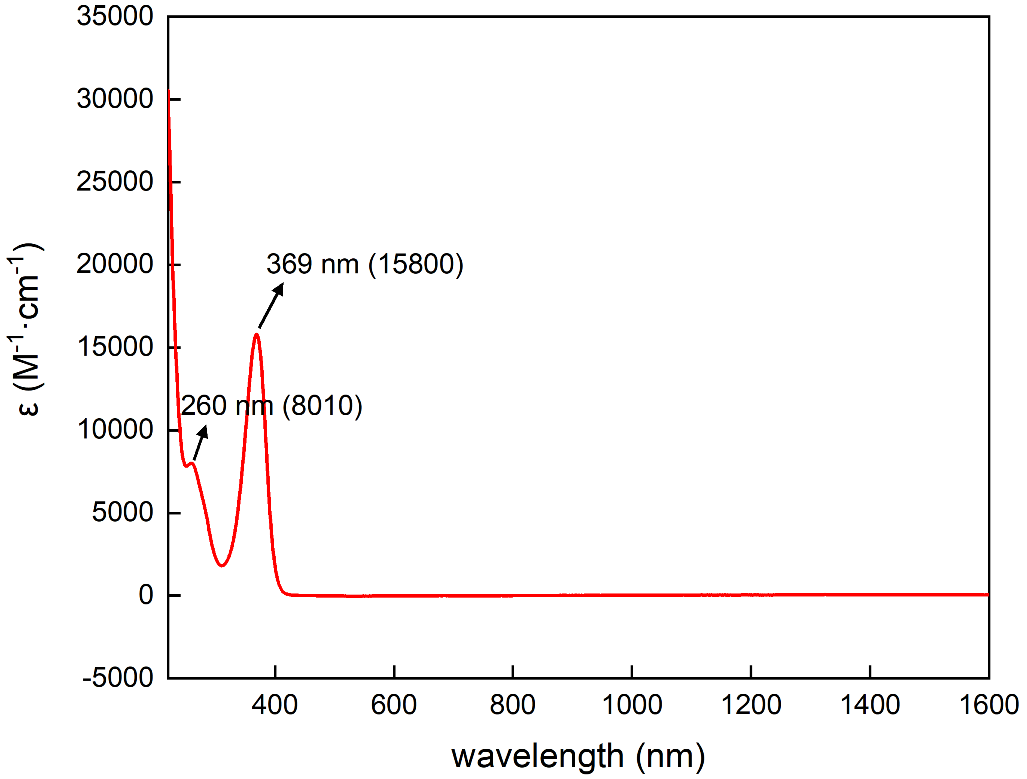

Fig. S22. UV–Vis–NIR spectrum (220–1600 nm) of (BDI)YI_2(THF) (**1-Y**) (298 K, 1.0 mM in Et_2O). λ_{max} / nm (ε / $M^{-1}\cdot cm^{-1}$): 260 (8010), 369 (15800).

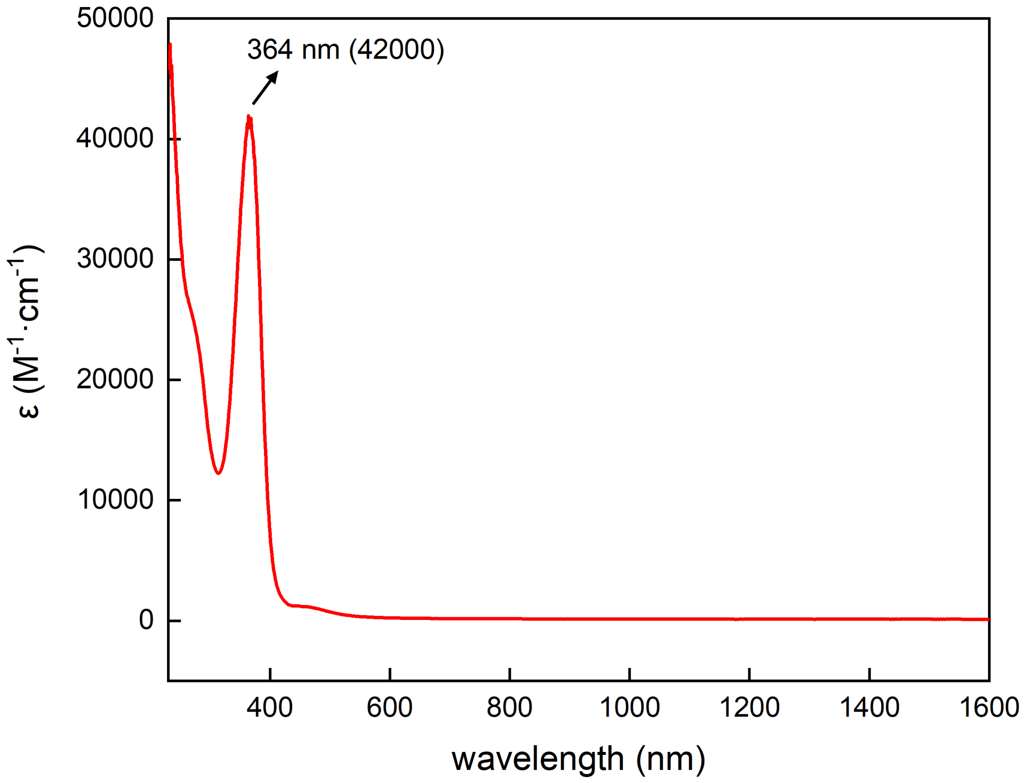

Fig. S23. UV–Vis–NIR spectrum (230–1600 nm) of (BDI)SmI_2(THF) (**1-Sm**) (298 K, 1.0 mM in Et_2O). λ_{max} / nm (ε / $M^{-1}\cdot cm^{-1}$): 364 (42000), 437–487 (1230–900).

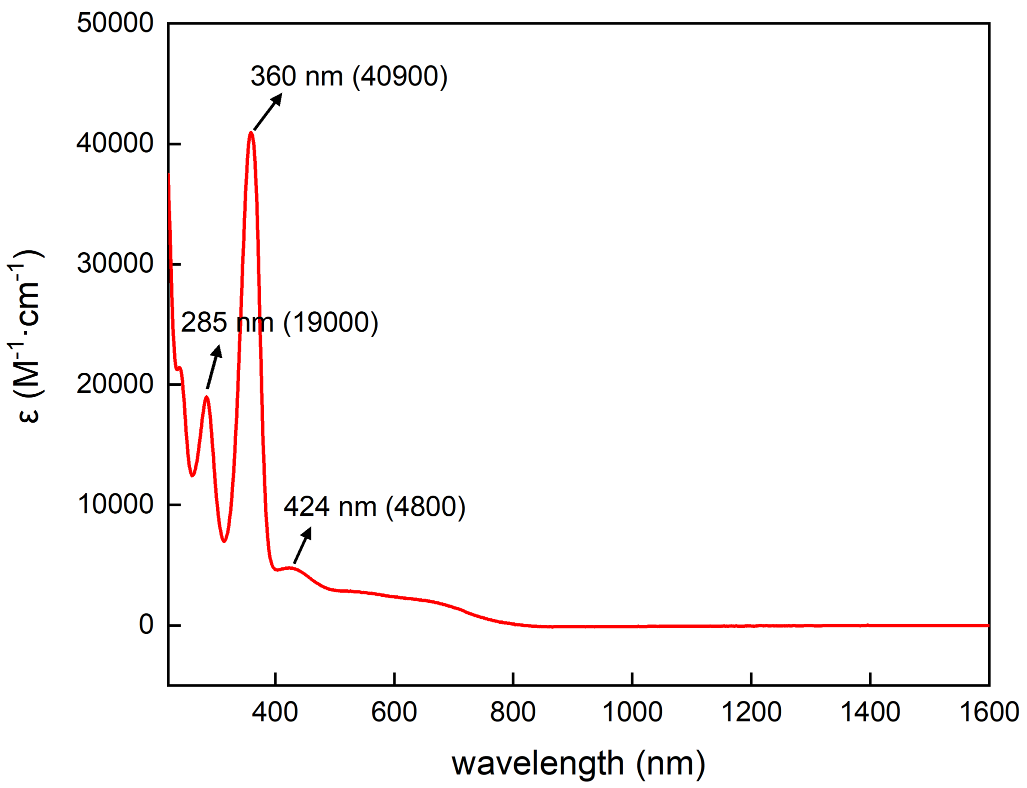

Fig. S24. UV–Vis–NIR spectrum (220–1600 nm) of [(BDI)Y]$_2$(μ–η^6,η^6-C_6H_6) (**2-Y**) (298 K, 0.5 mM in *n*-pentane). λ_{max} / nm (ε / $M^{-1}\cdot cm^{-1}$): 285 (19000), 360 (40900), 424 (4800), 500–726 (2920–1000).

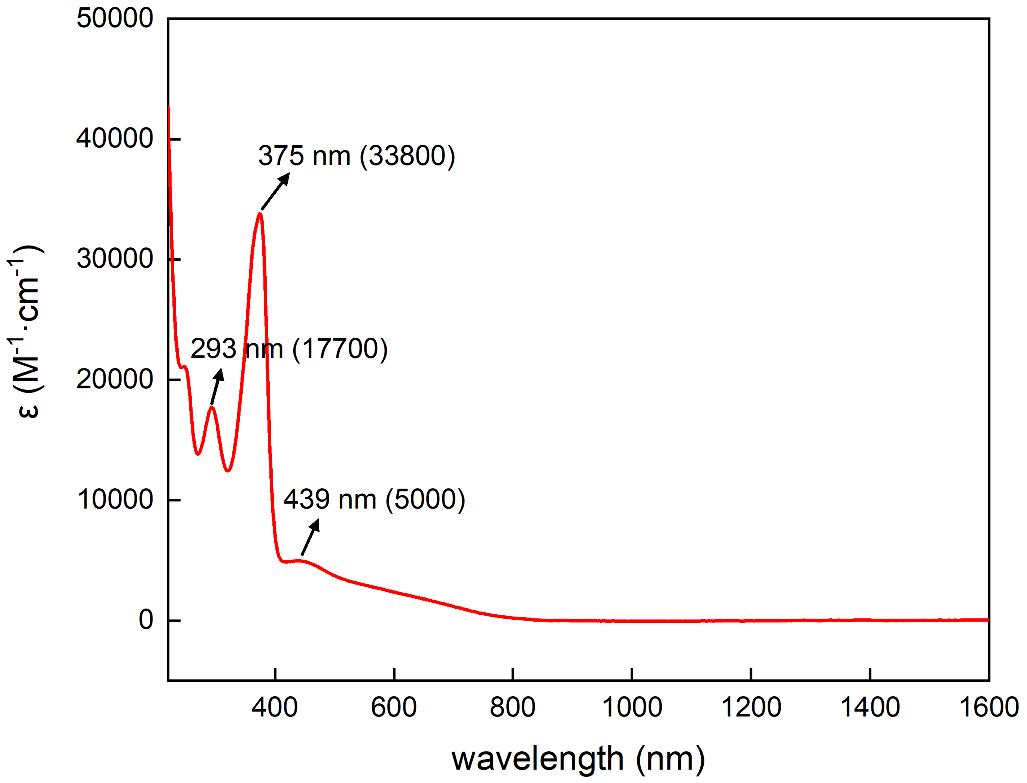

Fig. S25. UV–Vis–NIR spectrum (220–1600 nm) of [(BDI)Sm(THF)]$_2$(μ–η^6,η^6-C_6H_6) (**2-Sm**) (298 K, 0.5 mM in *n*-pentane). λ_{max} / nm (ε / $M^{-1}\cdot cm^{-1}$): 293 (17700), 375 (33800), 439 (5000), 523–651 (3300–1800).

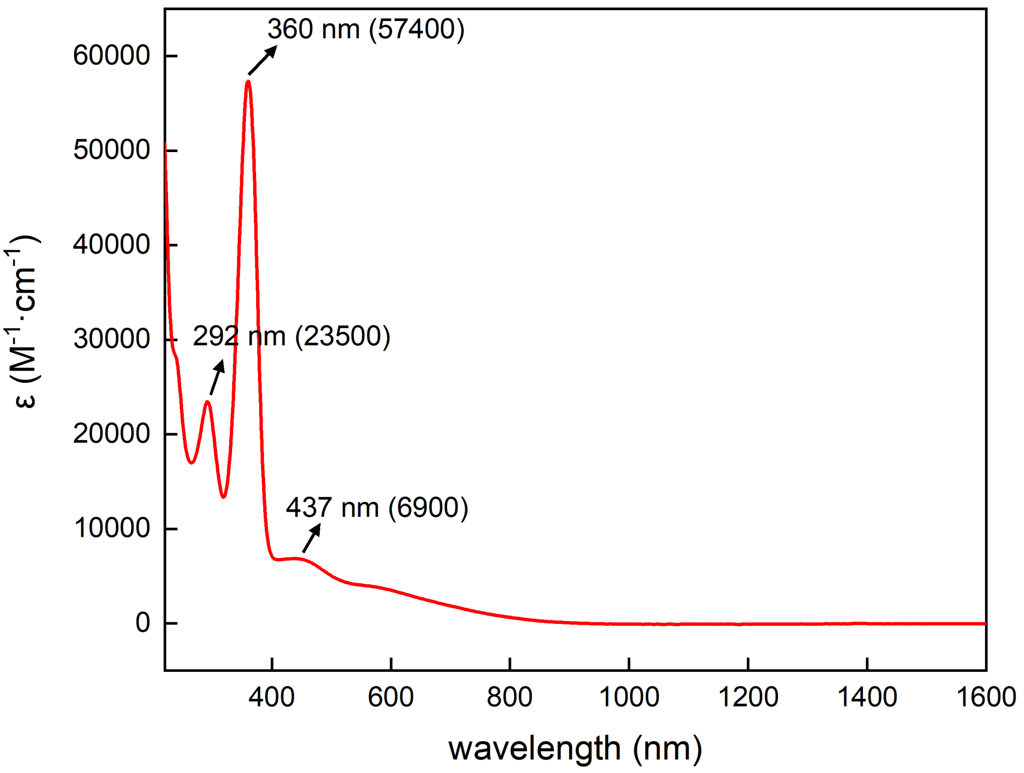

Fig. S26. UV–Vis–NIR spectrum (220–1600 nm) of $[(BDI)Y]_2(\mu\text{–}\eta^6,\eta^6\text{-}C_8H_{10})$ (**3-Y**) (298 K, 0.5 mM in *n*-pentane). λ_{max} / nm (ε / $M^{-1}\cdot cm^{-1}$): 292 (23500), 360 (57400), 437 (6900), 543–690 (4100–2000).

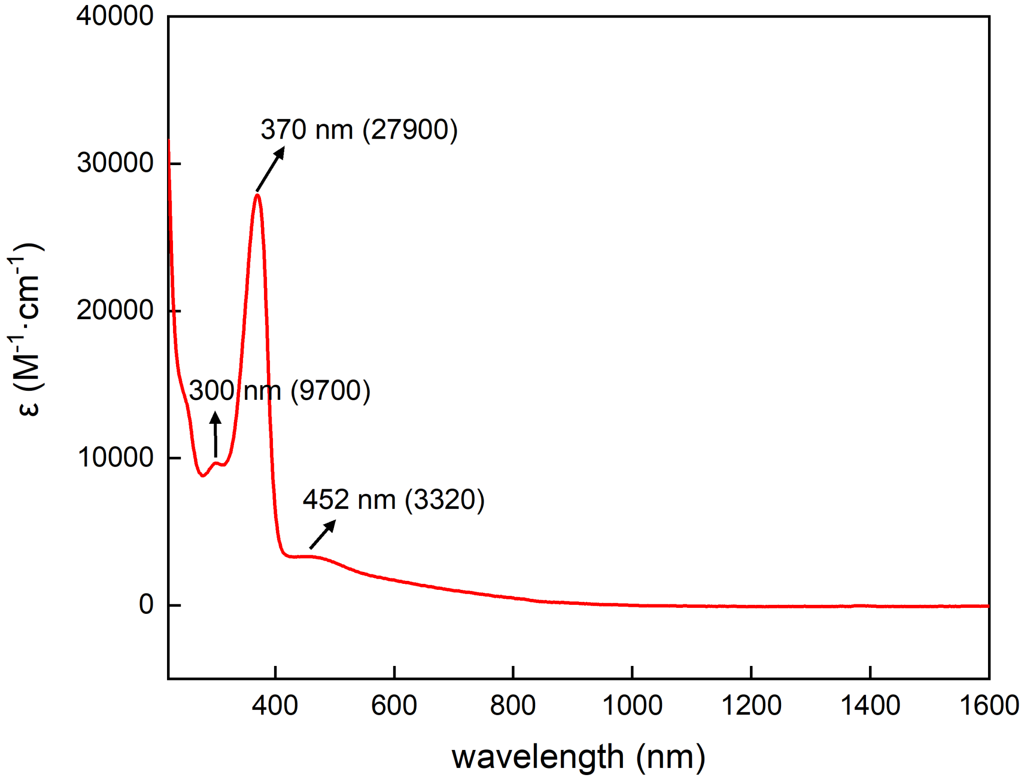

Fig. S27. UV–Vis–NIR spectrum (220–1600 nm) of [(BDI)Sm(THF)]$_2$(μ–η^6,η^6-C_8H_{10}) (**3-Sm**) (298 K, 0.5 mM in *n*-pentane). λ_{max} / nm (ε / $M^{-1}\cdot cm^{-1}$): 300 (9700), 370 (27900), 452 (3320), 545–705 (2180–1000).

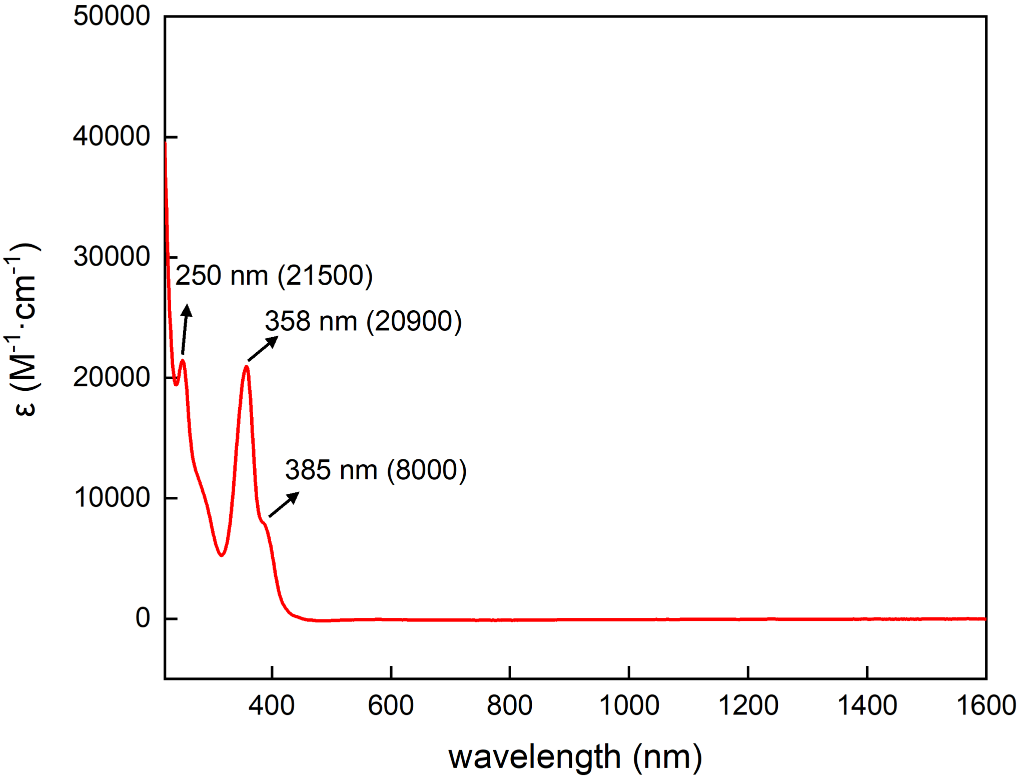

Fig. S28. UV–Vis–NIR spectrum (220–1600 nm) of (BDI)Sm(η^8-COT) (**4-Sm**) (298 K, 1.0 mM in *n*-pentane). λ_{max} / nm (ε / $M^{-1}\cdot cm^{-1}$): 250 (21500), 358 (20900), 385 (8000).

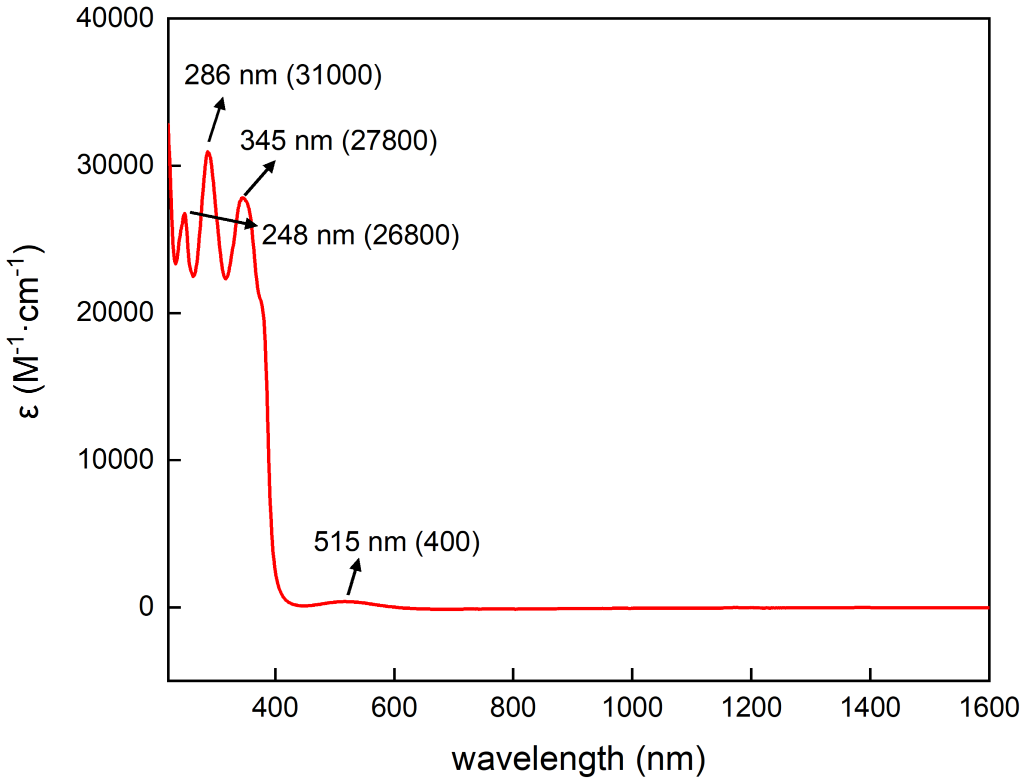

Fig. S29. UV–Vis–NIR spectrum (220–1600 nm) of (BDI)Sm(η^4-C_4Ph_2) (**5-Sm**) (298 K, 1.0 mM in *n*-pentane). λ_{max} / nm (ε / $M^{-1}\cdot cm^{-1}$): 248 (26800), 286 (31000), 345 (27800), 515 (400).

S5. IR Spectra

All IR spectra were recorded in the range of 4000–400 cm^{-1}.

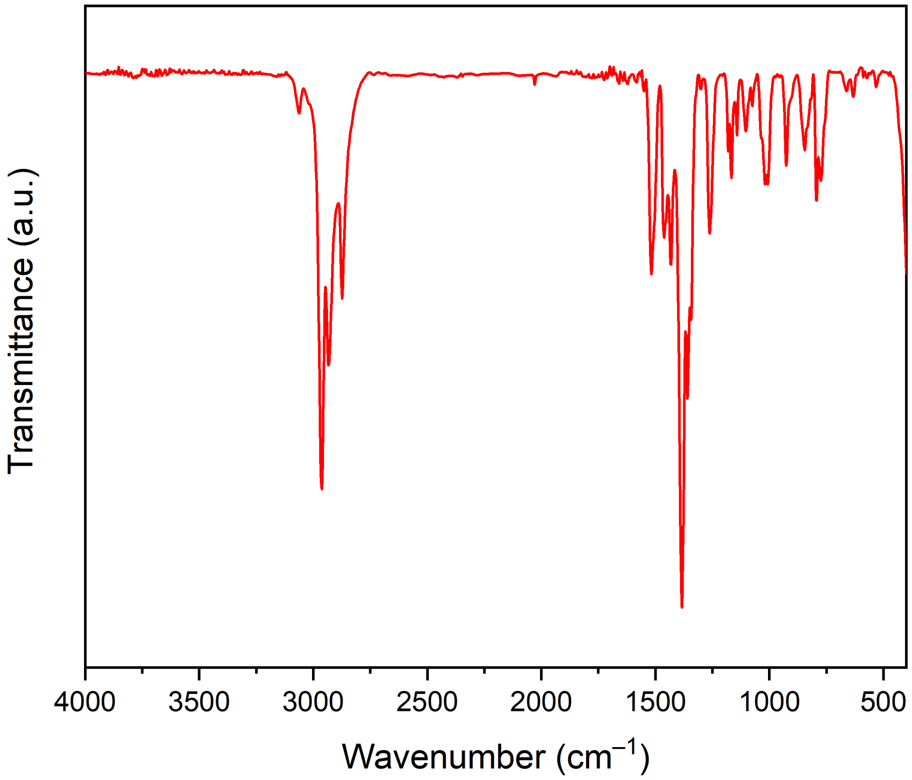

Fig. S30. IR spectrum of (BDI)YI_2(THF) (**1-Y**) in KBr, $\tilde{\nu}$ / cm^{-1}: 774 (w), 794 (w), 845 (w), 926 (w), 1019 (m), 1103 (w), 1142 (w), 1167 (w), 1180 (w), 1262 (m), 1359 (m), 1384 (s), 1432 (m), 1462 (m), 1518 (m), 2874 (m), 2933 (m), 2963 (s).

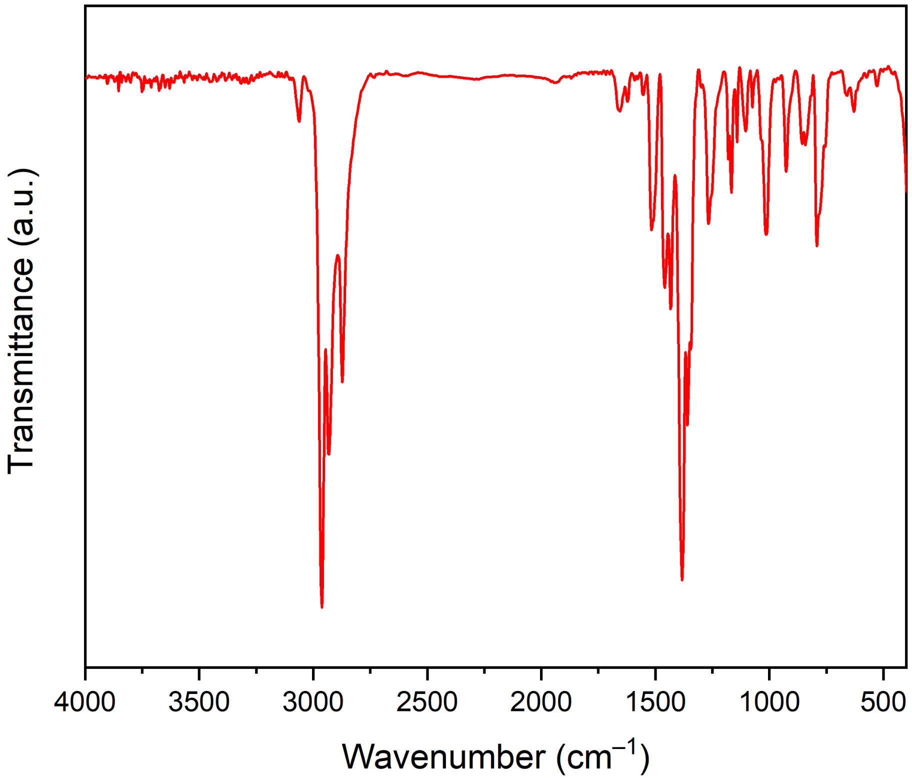

Fig. S31. IR spectrum of (BDI)SmI_2(THF) (**1-Sm**) in KBr, $\tilde{\nu}$ / cm^{-1}: 629 (w), 792 (m), 842 (w), 857 (w), 926 (w), 1014 (m), 1075 (w), 1104 (w), 1142 (w), 1167 (w), 1180 (w), 1267 (m), 1359 (m), 1383 (s), 1433 (m), 1459 (m), 1517 (m), 1533 (w), 1622 (w), 1655 (w), 2873 (m), 2932 (m), 2963 (s), 3062 (w).

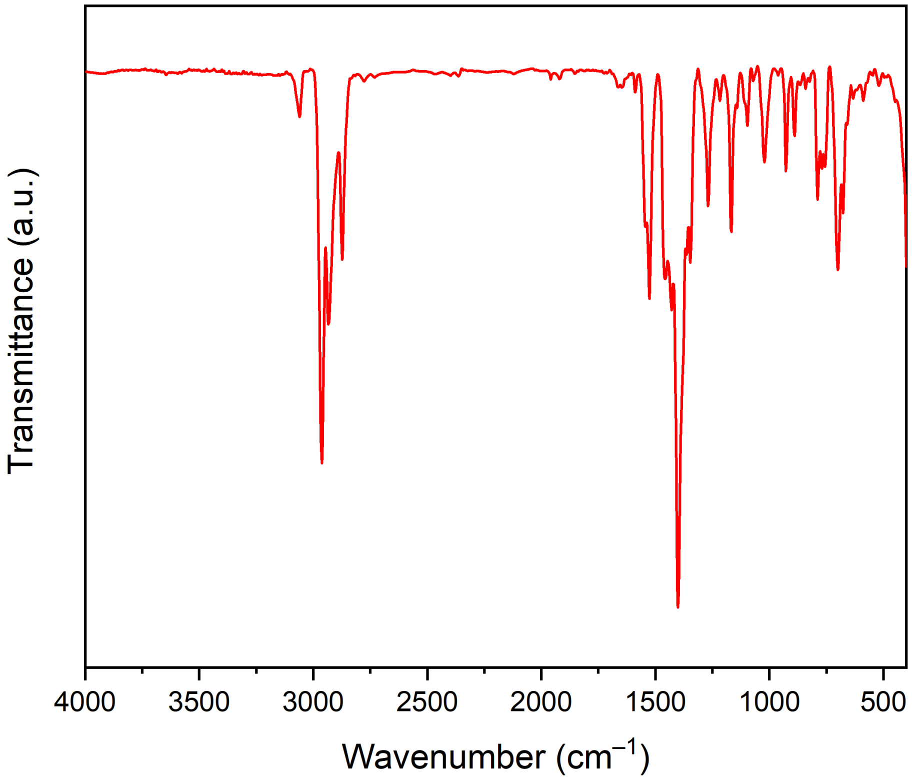

Fig. S32. IR spectrum of $[(BDI)Y]_2(\mu–\eta^6,\eta^6\text{-}C_6H_6)$ (**2-Y**) in KBr, $\tilde{\nu}$ / cm^{-1}: 677 (w), 700 (m), 789 (w), 890 (w), 927 (w), 1022 (m), 1097 (w), 1167 (m), 1268 (m), 1347 (w), 1401 (s), 1429 (w), 1526 (m), 2873 (m), 2933 (m), 2963 (s), 3060 (w).

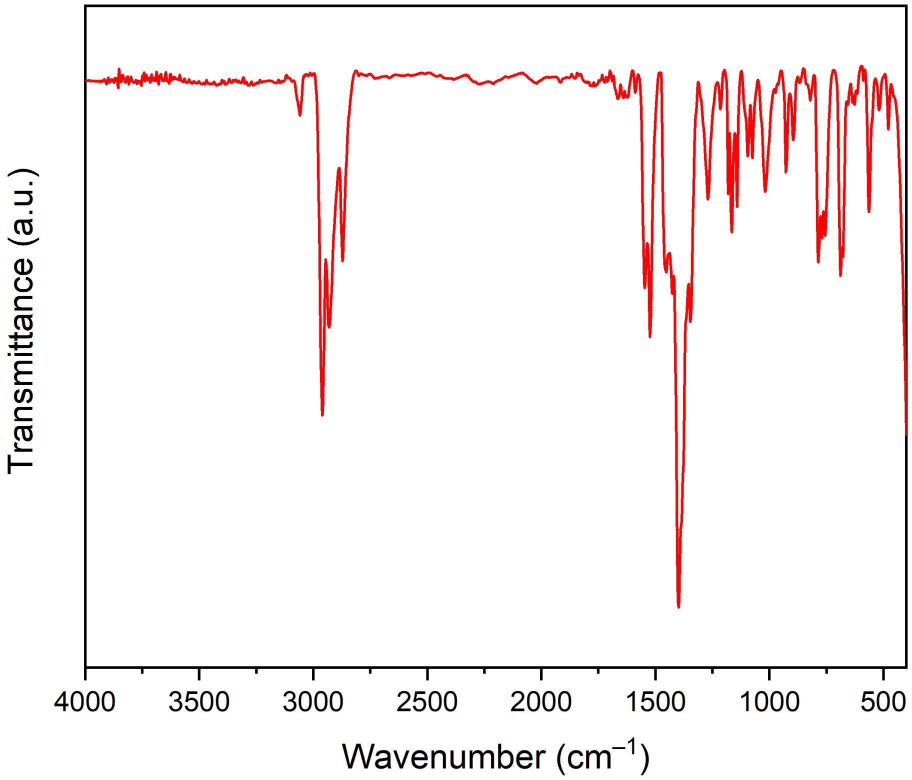

Fig. S33. IR spectrum of $[(BDI)Sm(THF)]_2(\mu\text{–}\eta^6,\eta^6\text{-}C_6H_6)$ (**2-Sm**) in KBr, $\tilde{\nu}$ / cm^{-1}: 478 (w), 563 (w), 688 (m), 755 (w), 770 (w), 786 (m), 895 (w), 927 (w), 1018 (m), 1075 (w), 1095 (w), 1142 (w), 1166 (m), 1180 (w), 1269 (m), 1346 (m), 1398 (s), 1453 (m), 1523 (m), 1546 (m), 2872 (m), 2931 (m), 2960 (s), 3059 (w).

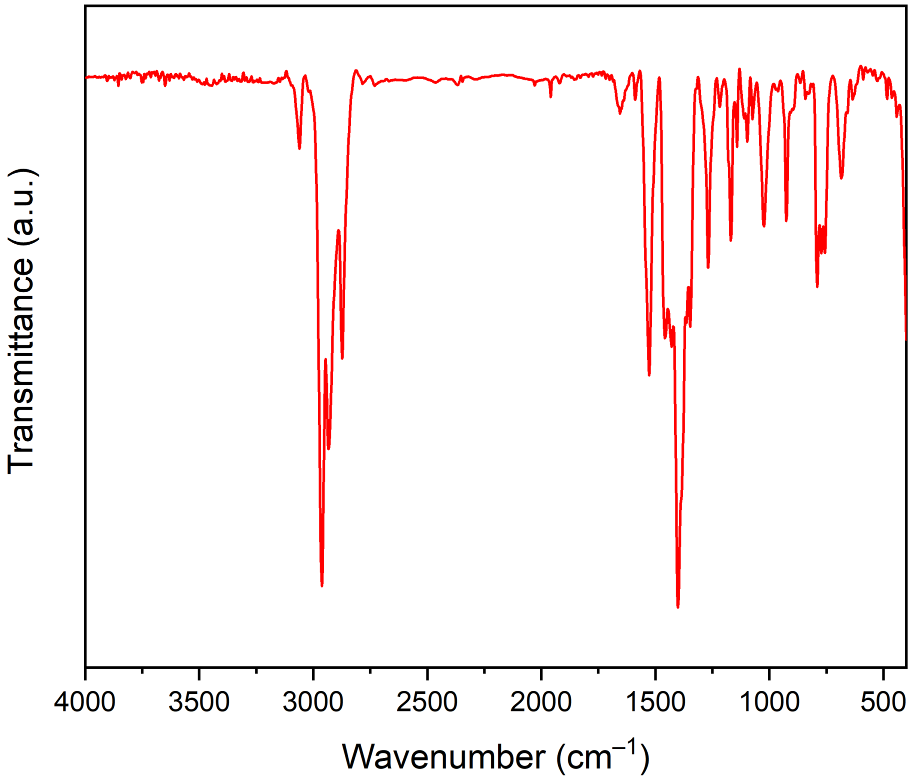

Fig. S34. IR spectrum of $[(BDI)Y]_2(\mu\text{–}\eta^6,\eta^6\text{-}C_8H_{10})$ (**3-Y**) in KBr, $\tilde{\nu}$ / cm^{-1}: 684 (w), 757 (m), 772 (w), 791 (m), 925 (m), 1024 (m), 1097 (w), 1142 (w), 1169 (m), 1268 (m), 1347 (m), 1401 (s), 1429 (w), 1457 (m), 1527 (m), 2873 (m), 2933 (m), 2962 (s), 3060 (w).

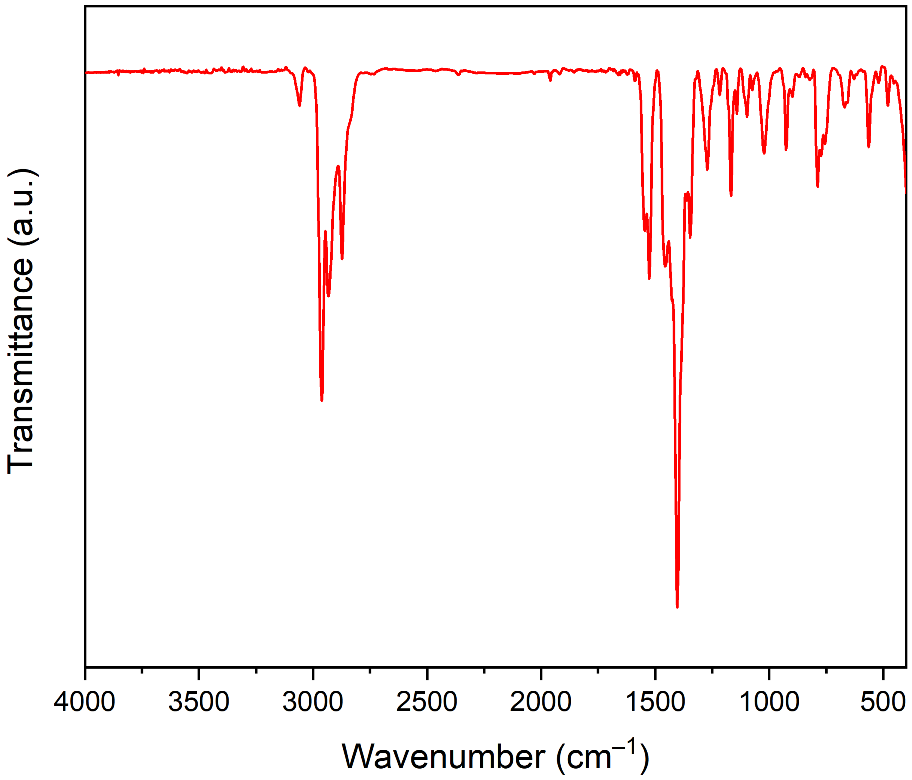

Fig. S35. IR spectrum of $[(BDI)Sm(THF)]_2(\mu–\eta^6,\eta^6-C_8H_{10})$ (**3-Sm**) in KBr, $\tilde{v}$ / cm^{-1}: 479 (w), 563 (788), 670 (w), 788 (m), 925 (w), 1022 (m), 1097 (w), 1141 (w), 1167 (w), 1217 (w), 1271 (m), 1346 (w), 1403 (s), 1456 (m), 1525 (m), 2873 (m), 2933 (m), 2962 (s). 3059 (w).

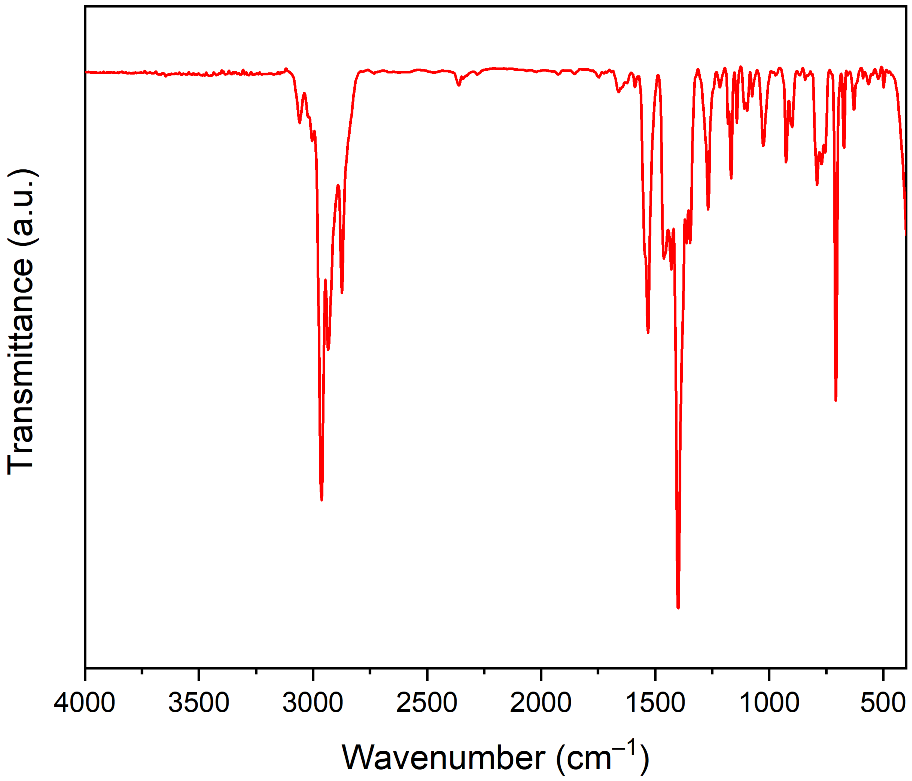

Fig. S36. IR spectrum of (BDI)Sm(η^8-COT) (**4-Sm**) in KBr, $\tilde{\nu}$ / cm^{-1}: 628 (w), 672 (w), 707 (m), 790 (m), 899 (w), 925 (w), 1026 (w), 1096 (w), 1142 (w), 1167 (w), 1267 (m), 1346 (w), 1362 (w), 1399 (s), 1429 (w), 1462 (w), 1531 (m), 2873 (m), 2933 (m), 2963 (s), 3029 (w).

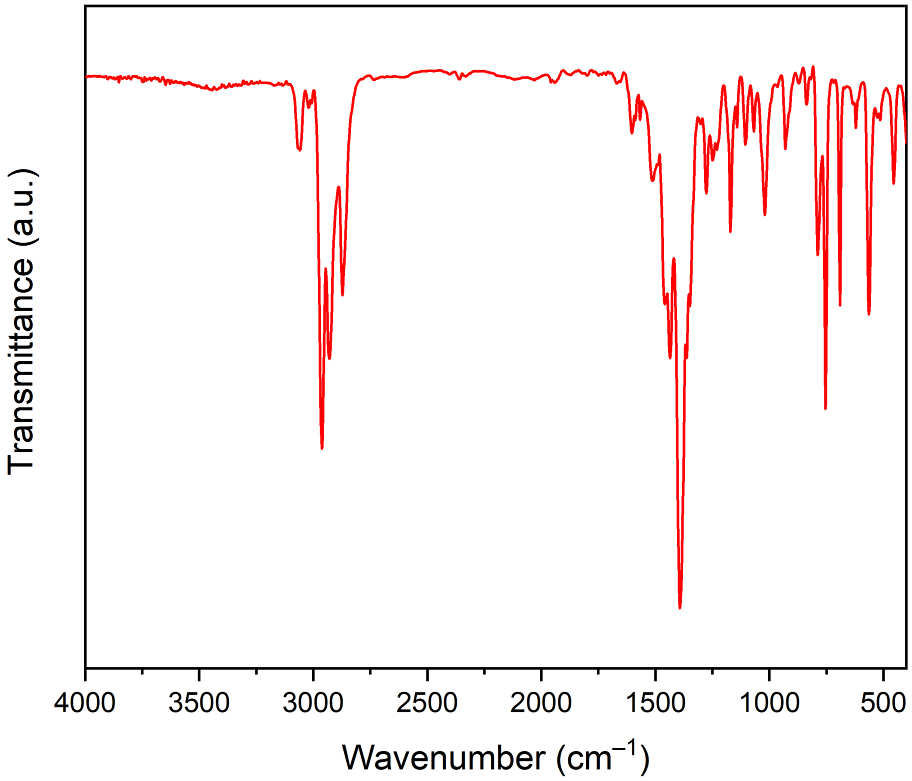

Fig. S37. IR spectrum of (BDI)Sm(η^4-C_4Ph_2) (**5-Sm**) in KBr, $\tilde{\nu}$ / cm^{-1}: 455 (w), 564 (m), 620 (w), 690 (m), 753 (m), 788 (w), 930 (w), 1020 (w), 1069 (w), 1105 (w), 1142 (w), 1170 (w), 1249 (w), 1276 (w), 1392 (s), 1435 (m), 1513 (w), 1603 (w), 2872 (m), 2930 (m), 2962 (s), 3058 (w).

S6. Density Functional Theory (DFT) Calculations

S6.1. Computational Details

DFT calculations were performed on the simplified models of neutral inverse-sandwich rare-earth metal complexes **2-M** and **3-M** (M = Y and Sm) by replacing 3-pentyl by methyl. All DFT calculations were performed ORCA v. 5.0.1,[11] with geometry optimization starting from the single crystal structures. The hybrid functional PBE0[12] with dispersion correction ("D3BJ"),[13, 14] was used and scalar relativistic effect was taken into account with the zeroth order regular approximation (ZORA).[15] For metals, segmented all-electron relativistically contracted (SARC) basis sets were chosen.[16, 17] For the bound arenes and other atoms directly bound to metals (N and O), adapted versions of the valence triple-zeta basis sets with polarization functions, def2-TZVP, were applied and for the other light atoms, the valence double-zeta basis sets with polarization functions, def2-SVP, were selected.[18] Single-point energy calculations were then performed with all atoms at triple-zeta level, except for **3-Sm** due to convergence problem (the single-point calculations of **3-Sm** was done with the same level of theory as geometry optimization). The natural bond orbital (NBO) analysis was carried out with NBO 6.0.[19] Representations of the molecular orbitals were rendered by VMD v. 1.9.3,[20] combined with the wave function analysis program Multiwfn.[21]

S6.2. Comparison between Experimental and Calculated Structures

Table. S3. Key structural parameters of **2-Y**.

Selected distances [Å] and torsion angles [°]	Exp.	Calc.	Diff.
Y1–N1	2.385(5)	2.332	–0.053
Y1–N2	2.355(5)	2.367	0.012
Y2–N3	2.391(4)	2.340	–0.051
Y3–N4	2.375(4)	2.366	–0.009
Y1–C1	2.411(5)	2.392	–0.019
Y1–C2	2.519(6)	2.524	0.005
Y1–C3	2.478(6)	2.450	–0.028
Y1–C4	2.420(5)	2.377	–0.043
Y1–C5	2.534(4)	2.514	–0.020
Y1–C6	2.467(4)	2.443	–0.024
Y2–C1	2.520(5)	2.521	0.001
Y2–C2	2.414(4)	2.394	–0.020
Y2–C3	2.464(4)	2.450	–0.014
Y2–C4	2.527(6)	2.513	–0.014
Y2–C5	2.411(6)	2.367	–0.044
Y2–C6	2.465(6)	2.443	–0.022
Y1–C_{cent}	1.996(1)	1.966	–0.030
Y2–C_{cent}	2.002(1)	1.964	–0.038
C1–C2	1.464(8)	1.464	0
C2–C3	1.436(8)	1.467	0.031
C3–C4	1.449(7)	1.465	0.016
C4–C5	1.465(7)	1.465	0
C5–C6	1.452(8)	1.467	0.015
C1–C6	1.444(7)	1.466	0.022
Avg. C–C (the bound ring)	1.452(8)	1.466	0.014
C3–C4/C5–C6	–13.1(8)	–15.9	–2.8
C1–C2/C4–C5	10.8(5)	14.0	3.2

Table. S4. Key structural parameters of **3-Y**.

Selected distances [Å] and torsion angles [°]	Exp.	Calc.	Diff.
Y1–N1	2.378(2)	2.351	–0.027
Y1–N2	2.392(3)	2.360	–0.032
Y2–N3	2.408(3)	2.392	–0.016
Y3–N4	2.414(3)	2.388	–0.026
Y1–C1	2.413(3)	2.382	–0.031
Y1–C2	2.514(4)	2.523	0.009
Y1–C3	2.498(3)	2.453	–0.045
Y1–C4	2.393(3)	2.373	–0.020
Y1–C5	2.500(4)	2.526	0.026
Y1–C6	2.501(4)	2.463	–0.038
Y2–C1	2.595(3)	2.572	–0.023
Y2–C2	2.436(4)	2.398	–0.038
Y2–C3	2.441(4)	2.450	0.009
Y2–C4	2.570(4)	2.568	–0.002
Y2–C5	2.432(4)	2.394	–0.038
Y2–C6	2.454(3)	2.451	–0.003
Y1–C_{cent}	1.999(1)	1.971	–0.028
Y2–C_{cent}	2.022(1)	1.995	–0.027
C1–C2	1.439(5)	1.466	0.027
C2–C3	1.458(5)	1.471	0.013
C3–C4	1.466(5)	1.465	–0.001
C4–C5	1.462(6)	1.467	0.005
C5–C6	1.445(6)	1.470	0.025
C1–C6	1.457(6)	1.463	0.006
Avg. C–C (the bound ring)	1.455(6)	1.467	0.012
C1–C7	1.519(5)	1.500	–0.019
C4–C8	1.513(5)	1.500	–0.013
C6–C1/C2–C3	–14.2(6)	–17.5	–3.3
C1–C2/C4–C5	12.4(3)	15.6	3.2

Table. S5. Key structural parameters of **2-Sm**.

Selected distances [Å] and torsion angles [°]	Exp.	**2-Sm (^{11}A)**		**2-Sm (^{13}A)**	
		Calc.	Diff.	Calc.	Diff.
Sm1–N1	2.510(2)	2.486	–0.024	2.541	0.031
Sm1–N2	2.510(2)	2.471	–0.039	2.467	–0.043
Sm2–N3	2.510(2)	2.521	0.011	2.528	0.018
Sm3–N4	2.510(2)	2.455	–0.055	2.458	–0.052
Sm1–C1	2.559(2)	2.544	–0.015	2.658	0.099
Sm1–C2	2.493(1)	2.470	–0.023	2.664	0.171
Sm1–C3	2.603(2)	2.564	–0.039	2.688	0.085
Sm1–C4	2.534(2)	2.519	–0.015	2.714	0.180
Sm1–C5	2.598(2)	2.582	–0.016	2.711	0.113
Sm1–C6	2.502(2)	2.494	–0.008	2.680	0.178
Sm2–C1	2.534(2)	2.513	–0.021	2.728	0.194
Sm2–C2	2.598(2)	2.581	–0.017	2.740	0.142
Sm2–C3	2.502(2)	2.484	–0.018	2.691	0.189
Sm2–C4	2.559(2)	2.543	–0.016	2.659	0.100
Sm2–C5	2.493(1)	2.494	0.001	2.651	0.158
Sm2–C6	2.603(2)	2.572	–0.031	2.671	0.068
Sm1–C_{cent}	2.095(1)	2.069	–0.026	2.269	0.174
Sm2–C_{cent}	2.095(1)	2.071	–0.024	2.274	0.179
Sm1–O	2.576(1)	2.560	–0.016	2.590	0.014
Sm2–O	2.576(1)	2.563	–0.013	2.582	0.006
C1–C2	1.453(3)	1.465	0.012	1.491	0.038
C2–C3	1.450(3)	1.454	0.004	1.408	–0.042
C3–C4	1.455(3)	1.451	–0.004	1.414	–0.041
C4–C5	1.453(3)	1.464	0.011	1.491	0.038
C5–C6	1.450(3)	1.454	0.004	1.409	–0.041
C1–C6	1.455(3)	1.453	–0.002	1.412	–0.043
Avg. C–C (the bound ring)	1.453(3)	1.457	0.004	1.437	–0.016
C3–C4/C5–C6	–12.8(3)	–12.1	0.7	–0.6	12.2

C1–C2/C4–C5	0(2)	–0.8	–0.8	0.6	0.6

Table. S6. Key structural parameters of **3-Sm**.

Selected distances [Å] and torsion angles [°]	Exp.	**3-Sm (^{11}A)**		**3-Sm (^{13}A)**	
		Calc.	Diff.	Calc.	Diff.
Sm1–N1	2.524(2)	2.489	–0.035	2.505	–0.019
Sm1–N2	2.541(2)	2.508	–0.033	2.518	–0.023
Sm2–N3	2.524(2)	2.515	–0.009	2.504	–0.020
Sm3–N4	2.541(2)	2.481	–0.060	2.518	–0.023
Sm1–C1	2.526(3)	2.557	0.031	2.676	0.150
Sm1–C2	2.598(3)	2.553	–0.045	2.730	0.132
Sm1–C3	2.594(2)	2.534	–0.060	2.765	0.171
Sm1–C4	2.580(2)	2.540	–0.040	2.770	0.190
Sm1–C5	2.544(2)	2.567	0.023	2.727	0.183
Sm1–C6	2.577(3)	2.519	–0.058	2.680	0.103
Sm2–C1	2.580(2)	2.527	–0.053	2.769	0.189
Sm2–C2	2.544(2)	2.526	–0.018	2.727	0.183
Sm2–C3	2.577(3)	2.519	–0.058	2.681	0.104
Sm2–C4	2.526(3)	2.541	0.015	2.678	0.152
Sm2–C5	2.598(3)	2.571	–0.027	2.732	0.134
Sm2–C6	2.594(2)	2.602	0.008	2.765	0.171
Sm1–C_{cent}	2.109(1)	2.089	–0.020	2.316	0.207
Sm2–C_{cent}	2.109(1)	2.093	–0.016	2.317	0.208
Sm1–O	2.585(3)	2.576	–0.009	2.571	–0.014
Sm2–O	2.585(3)	2.589	0.004	2.573	–0.012
C1–C2	1.450(4)	1.449	–0.001	1.405	–0.045
C2–C3	1.483(4)	1.450	–0.033	1.413	–0.070
C3–C4	1.475(3)	1.465	–0.010	1.489	0.014
C4–C5	1.450(4)	1.439	–0.011	1.405	–0.045
C5–C6	1.483(4)	1.455	–0.028	1.413	–0.070
C1–C6	1.475(3)	1.462	–0.013	1.489	0.014
Avg. C–C (the bound ring)	1.469(4)	1.453	–0.016	1.436	–0.033
C1–C7	1.513(3)	1.499	–0.014	1.495	–0.018

C4–C8	1.513(3)	1.497	–0.016	1.495	–0.018
C3–C4/C5–C6	5.1(4)	–2.5	–7.6	0.7	–4.4
C1–C2/C4–C5	0(3)	2.3	2.3	0	0

S6.3. Representations of Molecular Orbitals (MOs)

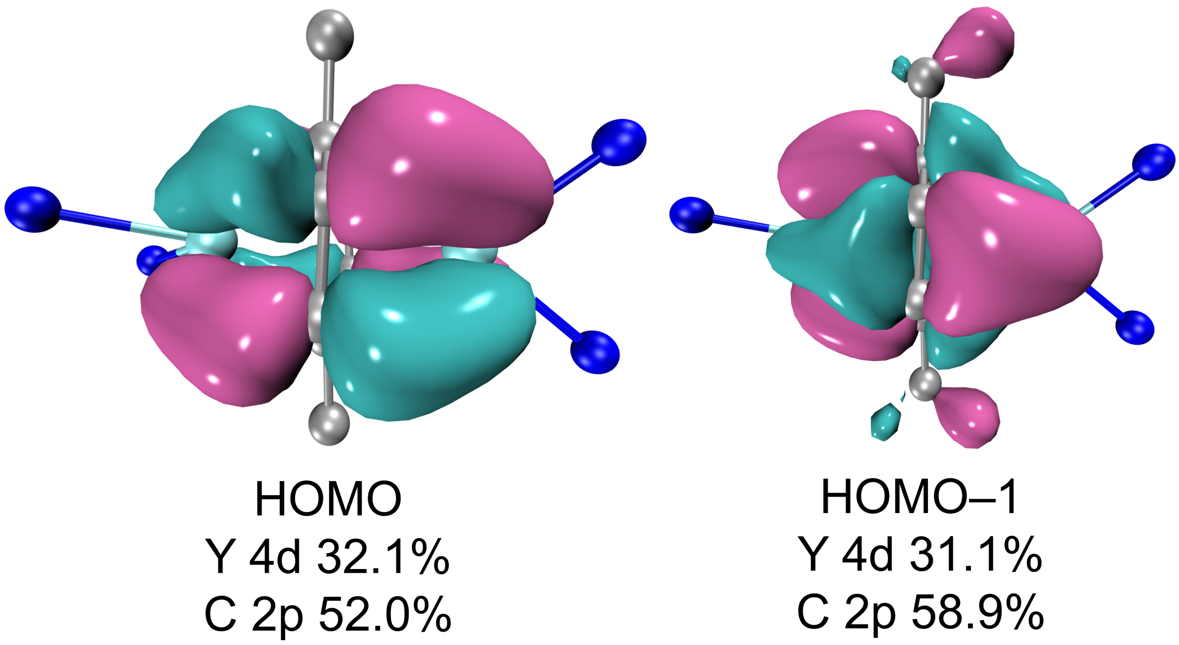

Fig. S38. Kohn-Sham orbitals (isovalue 0.04) with composition analysis for the frontier orbitals of **3-Y**. Only the metal, the atoms directly bound to the metal, and all carbon atoms of the bound arene are shown for clarity. Nitrogen in blue, oxygen in red, metal in cyan, and carbon in grey.

Table. S7. Energy levels of frontier orbitals of **2-Y** and **3-Y**.

E (eV)	**2-Y**	**3-Y**
HOMO	–3.55	–3.36
HOMO–1	–3.59	–3.65

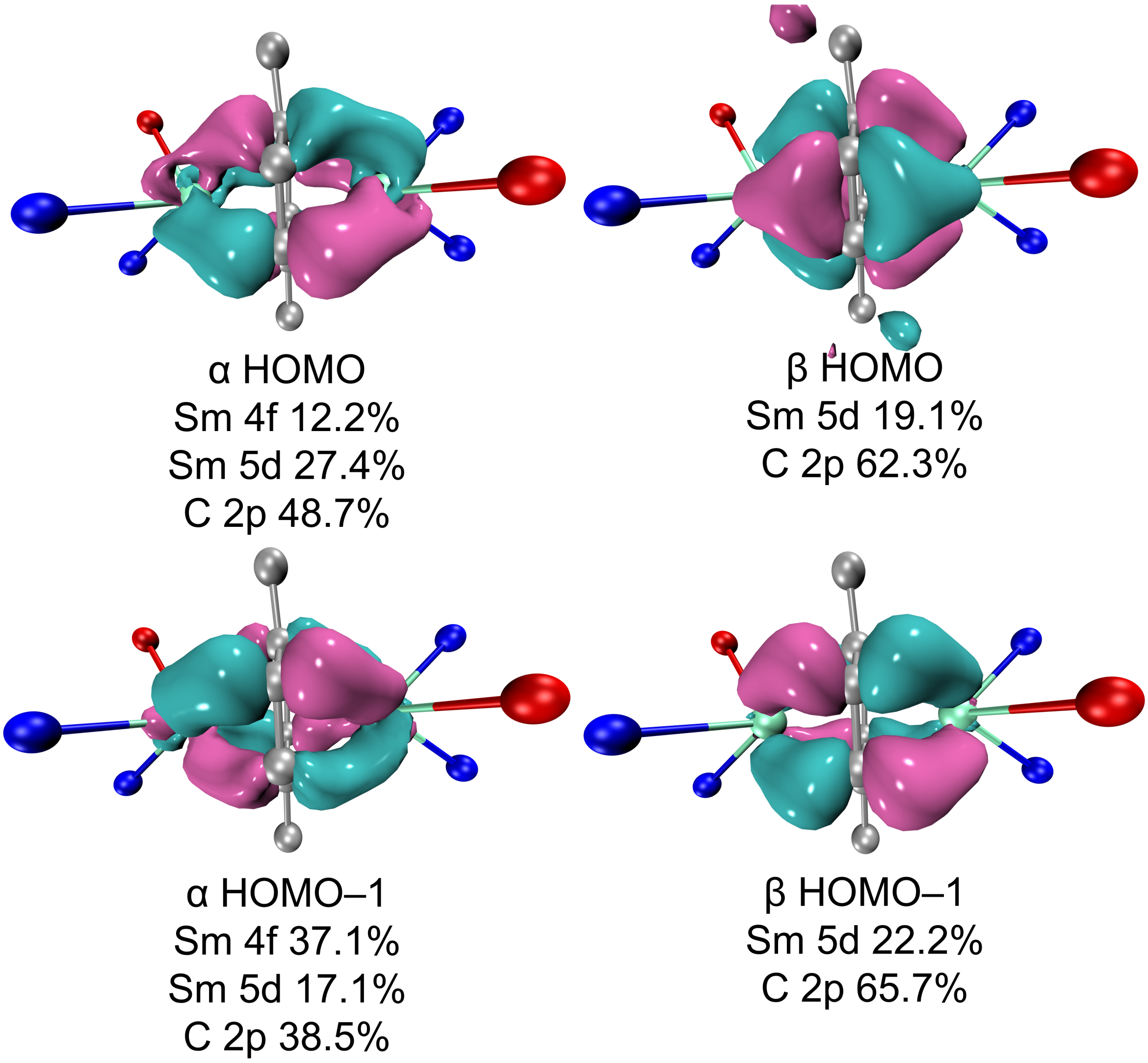

Fig. S39. Kohn-Sham orbitals (isovalue 0.04) with composition analysis for the frontier orbitals of **3-Sm** (**^{11}A**). Only the metal, the atoms directly bound to the metal, and all carbon atoms of the bound arene are shown for clarity. Nitrogen in blue, oxygen in red, metal in cyan, and carbon in grey.

Table. S8. Energy levels of frontier orbitals of **2-Sm** (**^{11}A**) and **3-Sm** (**^{11}A**).

E (eV)	**2-Sm (^{11}A)**		**3-Sm (^{11}A)**	
	α	β	α	β
HOMO	–2.93	–2.84	–2.68	–2.51
HOMO–1	–3.09	–2.96	–2.93	–2.83

S6.4. Population analysis

Table. S9. Natural population analysis for **2-Y**.

Atom	No.	Natural Charge	Core	Valence	Rydberg	Total
Y	1	1.56479	35.9734	1.36435	0.09746	37.43521
Y	2	1.56772	35.97335	1.36226	0.09668	37.43228
N	3	–0.67235	1.9992	5.65049	0.02266	7.67235
N	4	–0.67165	1.9992	5.64973	0.02273	7.67165
N	5	–0.67439	1.9992	5.6527	0.02249	7.67439
N	6	–0.67158	1.99921	5.64977	0.0226	7.67158
C	7	–0.67266	1.99944	4.66232	0.0109	6.67266
H	8	0.23027	0	0.76808	0.00165	0.76973
H	9	0.22339	0	0.77531	0.00129	0.77661
H	10	0.23019	0	0.76829	0.00152	0.76981
C	11	0.31452	1.99916	3.66651	0.01981	5.68548
C	12	–0.432	1.99909	4.41534	0.01756	6.432
H	13	0.21057	0	0.78742	0.002	0.78943
C	14	0.31474	1.99916	3.66625	0.01986	5.68526
C	15	–0.67229	1.99944	4.66185	0.01099	6.67229
H	16	0.22998	0	0.76837	0.00165	0.77002
H	17	0.22355	0	0.77518	0.00126	0.77645
H	18	0.22972	0	0.76873	0.00155	0.77028
C	19	0.12025	1.99879	3.85851	0.02245	5.87975
C	20	–0.02888	1.99907	4.01193	0.01788	6.02888
C	21	–0.21584	1.99914	4.20011	0.01659	6.21584
H	22	0.21192	0	0.7865	0.00159	0.78808
C	23	–0.21813	1.99923	4.20163	0.01727	6.21813
H	24	0.21578	0	0.78284	0.00137	0.78422
C	25	–0.21472	1.99915	4.19921	0.01636	6.21472
H	26	0.21225	0	0.78608	0.00167	0.78775
C	27	–0.02442	1.99906	4.00776	0.0176	6.02442

C	28	–0.62747	1.99944	4.61762	0.01042	6.62747
H	29	0.2274	0	0.77095	0.00165	0.7726
C	30	–0.63295	1.99944	4.62286	0.01065	6.63295
H	31	0.23385	0	0.7644	0.00175	0.76615
C	32	0.1193	1.99878	3.85959	0.02233	5.8807
C	33	–0.02773	1.99907	4.01074	0.01792	6.02773
C	34	–0.21575	1.99914	4.19995	0.01666	6.21575
H	35	0.21211	0	0.78631	0.00159	0.78789
C	36	–0.21809	1.99922	4.2016	0.01727	6.21809
H	37	0.216	0	0.78262	0.00138	0.784
C	38	–0.21481	1.99914	4.19916	0.01651	6.21481
H	39	0.21228	0	0.78605	0.00167	0.78772
C	40	–0.02501	1.99906	4.0083	0.01765	6.02501
C	41	–0.62757	1.99944	4.61774	0.01039	6.62757
H	42	0.22849	0	0.76989	0.00162	0.77151
C	43	–0.63055	1.99944	4.62049	0.01062	6.63055
H	44	0.22181	0	0.77635	0.00184	0.77819
C	45	–0.67187	1.99944	4.66137	0.01106	6.67187
H	46	0.22971	0	0.76867	0.00163	0.77029
H	47	0.22357	0	0.77517	0.00126	0.77643
H	48	0.22975	0	0.7687	0.00155	0.77025
C	49	0.31378	1.99915	3.66714	0.01993	5.68622
C	50	–0.43242	1.99909	4.41571	0.01762	6.43242
H	51	0.21034	0	0.78765	0.00201	0.78966
C	52	0.31366	1.99916	3.66731	0.01988	5.68634
C	53	–0.67211	1.99944	4.66182	0.01085	6.67211
H	54	0.23044	0	0.76795	0.00161	0.76956
H	55	0.22322	0	0.7755	0.00128	0.77678
H	56	0.22955	0	0.7689	0.00155	0.77045
C	57	0.11852	1.99878	3.86048	0.02222	5.88148
C	58	–0.02877	1.99907	4.01191	0.0178	6.02877

C	59	–0.21524	1.99914	4.19955	0.01654	6.21524
H	60	0.21212	0	0.78628	0.0016	0.78788
C	61	–0.21772	1.99923	4.20119	0.0173	6.21772
H	62	0.21596	0	0.78265	0.00139	0.78404
C	63	–0.21469	1.99914	4.19896	0.01658	6.21469
H	64	0.21227	0	0.78611	0.00162	0.78773
C	65	–0.02323	1.99907	4.00648	0.01769	6.02323
C	66	–0.62678	1.99944	4.61688	0.01046	6.62678
H	67	0.22665	0	0.77171	0.00163	0.77335
C	68	–0.63057	1.99944	4.62054	0.0106	6.63057
H	69	0.22105	0	0.77713	0.00183	0.77895
C	70	0.12341	1.9988	3.85541	0.02239	5.87659
C	71	–0.02867	1.99907	4.01182	0.01778	6.02867
C	72	–0.21622	1.99915	4.20052	0.01656	6.21622
H	73	0.21179	0	0.78663	0.00159	0.78821
C	74	–0.21952	1.99923	4.20295	0.01734	6.21952
H	75	0.21569	0	0.78293	0.00138	0.78431
C	76	–0.21428	1.99914	4.19877	0.01637	6.21428
H	77	0.21206	0	0.78628	0.00166	0.78794
C	78	–0.02592	1.99906	4.00922	0.01764	6.02592
C	79	–0.62728	1.99943	4.61761	0.01024	6.62728
H	80	0.22844	0	0.76991	0.00165	0.77156
C	81	–0.63215	1.99944	4.62208	0.01063	6.63215
H	82	0.22232	0	0.77588	0.0018	0.77768
H	83	0.22388	0	0.77432	0.0018	0.77612
H	84	0.21987	0	0.77882	0.00131	0.78013
H	85	0.21877	0	0.77989	0.00134	0.78123
H	86	0.22179	0	0.77638	0.00182	0.77821
H	87	0.2231	0	0.7751	0.0018	0.7769
H	88	0.21943	0	0.77925	0.00131	0.78057
H	89	0.23165	0	0.76662	0.00172	0.76835

H	90	0.21904	0	0.77962	0.00134	0.78096
H	91	0.2238	0	0.7744	0.00181	0.7762
H	92	0.22	0	0.77869	0.00132	0.78
H	93	0.2187	0	0.78	0.0013	0.7813
H	94	0.23309	0	0.76517	0.00174	0.76691
H	95	0.22192	0	0.77624	0.00184	0.77808
H	96	0.21841	0	0.7803	0.00129	0.78159
H	97	0.21929	0	0.77938	0.00133	0.78071
H	98	0.23208	0	0.76618	0.00174	0.76792
C	99	–0.57775	1.99883	4.55246	0.02646	6.57775
H	100	0.26438	0	0.73403	0.00159	0.73562
C	101	–0.58027	1.99877	4.55328	0.02821	6.58027
H	102	0.27112	0	0.72699	0.00189	0.72888
C	103	–0.58369	1.99878	4.55704	0.02787	6.58369
H	104	0.2717	0	0.72635	0.00194	0.7283
C	105	–0.58502	1.99882	4.55955	0.02665	6.58502
H	106	0.26545	0	0.73295	0.00159	0.73455
C	107	–0.58256	1.99878	4.55547	0.02831	6.58256
H	108	0.27138	0	0.72671	0.0019	0.72862
C	109	–0.57877	1.99879	4.55203	0.02796	6.57877
H	110	0.27032	0	0.72777	0.00191	0.72968
Total		0.00000	175.90213	272.88291	1.21496	450.00000

Average charge on yttrium: 1.57

Total charge of the bound benzene (C+H)/(C): –1.87/–3.49

Average charge on nitrogen donors of the BDI ligand: –0.67

Table. S10. Natural population analysis for **3-Y**.

Atom	No.	Natural Charge	Core	Valence	Rydberg	Total
Y	1	1.65854	35.96993	1.24543	0.1261	37.34146
Y	2	1.7125	35.9727	1.21659	0.0982	37.2875
N	3	–0.68441	1.99921	5.66225	0.02295	7.68441
N	4	–0.68076	1.99921	5.65836	0.02319	7.68076
N	5	–0.69831	1.99927	5.67612	0.02292	7.69831
N	6	–0.69964	1.99927	5.67746	0.02291	7.69964
C	7	–0.67097	1.99944	4.66068	0.01085	6.67097
H	8	0.22922	0	0.76926	0.00152	0.77078
H	9	0.22371	0	0.77502	0.00128	0.77629
H	10	0.2288	0	0.76954	0.00166	0.7712
C	11	0.31213	1.99914	3.66841	0.02032	5.68787
C	12	–0.42917	1.99909	4.4123	0.01778	6.42917
H	13	0.21084	0	0.78709	0.00207	0.78916
C	14	0.31244	1.99914	3.66809	0.02033	5.68756
C	15	–0.67082	1.99944	4.6606	0.01077	6.67082
H	16	0.22912	0	0.76935	0.00152	0.77088
H	17	0.22347	0	0.77525	0.00127	0.77653
H	18	0.2289	0	0.7694	0.0017	0.7711
C	19	0.11551	1.99876	3.86287	0.02286	5.88449
C	20	–0.02716	1.99906	4.01007	0.01803	6.02716
C	21	–0.21644	1.99914	4.20042	0.01688	6.21644
H	22	0.21414	0	0.78417	0.00169	0.78586
C	23	–0.21781	1.99923	4.20125	0.01733	6.21781
H	24	0.21691	0	0.78171	0.00138	0.78309
C	25	–0.21427	1.99914	4.19851	0.01661	6.21427
H	26	0.2131	0	0.7853	0.0016	0.7869
C	27	–0.02895	1.99907	4.0117	0.01818	6.02895
C	28	–0.62632	1.99944	4.61566	0.01122	6.62632

H	29	0.22703	0	0.77109	0.00189	0.77297
C	30	–0.62465	1.99944	4.61468	0.01053	6.62465
H	31	0.22645	0	0.77191	0.00164	0.77355
C	32	0.11995	1.99877	3.85834	0.02294	5.88005
C	33	–0.02712	1.99905	4.01004	0.01802	6.02712
C	34	–0.21696	1.99914	4.20108	0.01674	6.21696
H	35	0.214	0	0.7843	0.0017	0.786
C	36	–0.21932	1.99922	4.20276	0.01733	6.21932
H	37	0.21678	0	0.78184	0.00138	0.78322
C	38	–0.21551	1.99914	4.19966	0.01671	6.21551
H	39	0.21295	0	0.78545	0.0016	0.78705
C	40	–0.02989	1.99907	4.01268	0.01815	6.02989
C	41	–0.62812	1.99944	4.61746	0.01122	6.62812
H	42	0.22818	0	0.76992	0.0019	0.77182
C	43	–0.6211	1.99944	4.61127	0.01038	6.6211
H	44	0.22421	0	0.7741	0.00169	0.77579
C	45	–0.67431	1.99944	4.66362	0.01125	6.67431
H	46	0.23192	0	0.76644	0.00164	0.76808
H	47	0.22256	0	0.77618	0.00126	0.77744
H	48	0.23215	0	0.76615	0.0017	0.76785
C	49	0.30957	1.99914	3.67085	0.02044	5.69043
C	50	–0.43407	1.99908	4.41674	0.01824	6.43407
H	51	0.20978	0	0.78797	0.00225	0.79022
C	52	0.30985	1.99914	3.67066	0.02036	5.69015
C	53	–0.67414	1.99944	4.66367	0.01103	6.67414
H	54	0.23179	0	0.76657	0.00163	0.76821
H	55	0.22248	0	0.77621	0.0013	0.77752
H	56	0.23226	0	0.76605	0.00169	0.76774
C	57	0.12972	1.99881	3.84853	0.02294	5.87028
C	58	–0.03049	1.99904	4.01326	0.01819	6.03049
C	59	–0.21513	1.99913	4.19881	0.01719	6.21513

H	60	0.2128	0	0.78559	0.00161	0.7872
C	61	–0.21789	1.99922	4.2011	0.01757	6.21789
H	62	0.2168	0	0.78179	0.00141	0.7832
C	63	–0.21739	1.99913	4.20094	0.01732	6.21739
H	64	0.21323	0	0.78521	0.00156	0.78677
C	65	–0.03579	1.99904	4.01841	0.01834	6.03579
C	66	–0.63398	1.99944	4.62308	0.01146	6.63398
H	67	0.22314	0	0.77502	0.00183	0.77686
C	68	–0.63026	1.99944	4.62001	0.01081	6.63026
H	69	0.22904	0	0.76931	0.00165	0.77096
C	70	0.13037	1.99881	3.8478	0.02303	5.86963
C	71	–0.02924	1.99904	4.01225	0.01794	6.02924
C	72	–0.21435	1.99913	4.19837	0.01685	6.21435
H	73	0.21257	0	0.78579	0.00164	0.78743
C	74	–0.21818	1.99922	4.20155	0.01741	6.21818
H	75	0.21662	0	0.78198	0.0014	0.78338
C	76	–0.21782	1.99914	4.20164	0.01704	6.21782
H	77	0.21326	0	0.78516	0.00158	0.78674
C	78	–0.03654	1.99904	4.01931	0.01819	6.03654
C	79	–0.63386	1.99944	4.62322	0.0112	6.63386
H	80	0.22286	0	0.77528	0.00186	0.77714
C	81	–0.63019	1.99944	4.62008	0.01067	6.63019
H	82	0.22867	0	0.76969	0.00164	0.77133
H	83	0.22187	0	0.77619	0.00194	0.77813
H	84	0.22138	0	0.77717	0.00145	0.77862
H	85	0.22063	0	0.77804	0.00133	0.77937
H	86	0.22455	0	0.7737	0.00176	0.77545
H	87	0.22097	0	0.77707	0.00196	0.77903
H	88	0.22133	0	0.77718	0.00148	0.77867
H	89	0.22009	0	0.77859	0.00131	0.77991
H	90	0.22354	0	0.77466	0.0018	0.77646

H	91	0.21772	0	0.78094	0.00134	0.78228
H	92	0.23487	0	0.76327	0.00187	0.76513
H	93	0.2183	0	0.78037	0.00132	0.7817
H	94	0.22404	0	0.77412	0.00184	0.77596
H	95	0.21759	0	0.78105	0.00136	0.78241
H	96	0.23533	0	0.76284	0.00184	0.76467
H	97	0.21831	0	0.78036	0.00133	0.78169
H	98	0.22389	0	0.77423	0.00188	0.77611
C	99	–0.59855	1.99942	4.58631	0.01282	6.59855
H	100	0.20562	0	0.7925	0.00187	0.79438
H	101	0.22314	0	0.77398	0.00288	0.77686
H	102	0.21608	0	0.78177	0.00215	0.78392
C	103	–0.39567	1.99876	4.36515	0.03175	6.39567
C	104	–0.59955	1.99883	4.57193	0.02879	6.59955
C	105	–0.60228	1.99889	4.57698	0.02641	6.60228
C	106	–0.39955	1.99876	4.36918	0.03161	6.39955
C	107	–0.59791	1.99883	4.57013	0.02895	6.59791
C	108	–0.59617	1.99889	4.5707	0.02658	6.59617
C	109	–0.59858	1.99942	4.58619	0.01296	6.59858
H	110	0.2042	0	0.79375	0.00205	0.7958
H	111	0.22343	0	0.77381	0.00277	0.77657
H	112	0.21776	0	0.78011	0.00213	0.78224
H	113	0.26538	0	0.733	0.00162	0.73462
H	114	0.27234	0	0.72558	0.00208	0.72766
H	115	0.26563	0	0.73275	0.00161	0.73437
H	116	0.27126	0	0.72671	0.00203	0.72874
Total		0.00000	179.89694	284.79409	1.30896	466.00000

Average charge on yttrium: 1.69

Total charge of the bound *p*-xylene excluding two methyl groups (C+H)/(C): –2.02/–3.19

Average charge on nitrogen donors of the BDI ligand: –0.69

Table. S11. Natural population analysis for **2-Sm (**^{11}**A)**.

Atom	No.	Natural Charge	Core	Valence	Rydberg	Total	Natural Spin Density
Sm	1	1.21576	53.95759	6.3465	0.48015	60.78424	5.13626
O	2	–0.53232	1.99963	6.50743	0.02527	8.53232	0.00414
N	3	–0.65186	1.99903	5.62202	0.03081	7.65186	0.00141
N	4	–0.6449	1.99902	5.61437	0.03151	7.6449	–0.00356
C	5	–0.67324	1.99944	4.66277	0.01103	6.67324	–0.00051
H	6	0.23164	0	0.76668	0.00168	0.76836	0.00016
H	7	0.2218	0	0.77695	0.00125	0.7782	–0.00007
H	8	0.22517	0	0.77322	0.00161	0.77483	0.00019
C	9	0.31818	1.99907	3.66196	0.02079	5.68182	0.0042
C	10	–0.44746	1.99907	4.42977	0.01863	6.44746	–0.00302
H	11	0.20851	0	0.78959	0.0019	0.79149	0.00023
C	12	0.32074	1.99906	3.65972	0.02048	5.67926	0.00526
C	13	–0.673	1.99943	4.66249	0.01108	6.673	–0.00068
H	14	0.22621	0	0.77216	0.00163	0.77379	0.00017
H	15	0.22115	0	0.77755	0.00129	0.77885	–0.00011
H	16	0.23363	0	0.76489	0.00148	0.76637	0.00019
C	17	0.13974	1.99869	3.83568	0.02589	5.86026	0.00567
C	18	–0.03405	1.99905	4.01599	0.01901	6.03405	–0.00039
C	19	–0.20805	1.99914	4.19268	0.01623	6.20805	0.00062
H	20	0.2098	0	0.78856	0.00164	0.7902	–0.00001
C	21	–0.22233	1.99922	4.20619	0.01692	6.22233	–0.00112
H	22	0.21403	0	0.78459	0.00138	0.78597	0.00004
C	23	–0.21368	1.99913	4.1979	0.01664	6.21368	0.00054
H	24	0.20878	0	0.78964	0.00158	0.79122	–0.00002
C	25	–0.02752	1.99907	4.00866	0.01979	6.02752	–0.00011
C	26	–0.61817	1.99944	4.60745	0.01127	6.61817	0.00049
H	27	0.21774	0	0.78027	0.00199	0.78226	0

C	28	–0.6206	1.99943	4.61082	0.01035	6.6206	0.00037
H	29	0.23197	0	0.76576	0.00227	0.76803	0.0019
C	30	0.13419	1.9987	3.84085	0.02626	5.86581	0.00503
C	31	–0.02399	1.99906	4.00502	0.0199	6.02399	0.00172
C	32	–0.21602	1.99913	4.20037	0.01652	6.21602	–0.00079
H	33	0.2078	0	0.79063	0.00156	0.7922	0.00002
C	34	–0.21695	1.99922	4.20073	0.017	6.21695	0.00135
H	35	0.21158	0	0.78693	0.00149	0.78842	–0.00004
C	36	–0.20453	1.99914	4.18952	0.01586	6.20453	–0.00098
H	37	0.20858	0	0.78965	0.00177	0.79142	0.00004
C	38	–0.03356	1.99905	4.01591	0.0186	6.03356	0.00176
C	39	–0.6237	1.99943	4.61337	0.0109	6.6237	0.00005
H	40	0.23103	0	0.76669	0.00228	0.76897	0.00163
C	41	–0.61907	1.99944	4.6088	0.01083	6.61907	0.00058
H	42	0.21662	0	0.7814	0.00199	0.78338	0.00016
C	43	–0.07093	1.99926	4.05529	0.01637	6.07093	0.00139
H	44	0.22724	0	0.77045	0.00231	0.77276	0.00028
H	45	0.18161	0	0.81623	0.00216	0.81839	0.0002
C	46	–0.43231	1.99946	4.42215	0.0107	6.43231	–0.0001
H	47	0.22634	0	0.77223	0.00142	0.77366	–0.00009
H	48	0.21288	0	0.78556	0.00156	0.78712	0.00001
C	49	–0.43515	1.99946	4.42557	0.01011	6.43515	0.00013
H	50	0.22627	0	0.77231	0.00142	0.77373	–0.00002
H	51	0.20943	0	0.78894	0.00163	0.79057	–0.00003
C	52	–0.07005	1.99931	4.05295	0.01779	6.07005	0.00187
H	53	0.20087	0	0.79718	0.00195	0.79913	0.00016
H	54	0.19316	0	0.80472	0.00212	0.80684	0.00024
Sm	55	1.21694	53.95728	6.36056	0.46522	60.78306	5.16754
O	56	–0.53572	1.99964	6.51225	0.02383	8.53572	0.00626
N	57	–0.64431	1.99906	5.61255	0.03271	7.64431	0.00039
N	58	–0.64939	1.99901	5.62012	0.03025	7.64939	–0.00247

C	59	–0.67473	1.99944	4.66422	0.01108	6.67473	–0.00072
H	60	0.23284	0	0.76549	0.00168	0.76716	0.00026
H	61	0.22045	0	0.77824	0.00131	0.77955	–0.00013
H	62	0.22634	0	0.77209	0.00157	0.77366	0.00027
C	63	0.31664	1.99908	3.66348	0.0208	5.68336	0.00634
C	64	–0.44919	1.99906	4.43139	0.01874	6.44919	–0.00266
H	65	0.20823	0	0.78986	0.00191	0.79177	0.00022
C	66	0.31856	1.99905	3.66172	0.02067	5.68144	0.00712
C	67	–0.67114	1.99943	4.6608	0.0109	6.67114	–0.00082
H	68	0.22408	0	0.77428	0.00165	0.77592	0.00027
H	69	0.22188	0	0.77687	0.00125	0.77812	–0.0001
H	70	0.23234	0	0.76616	0.0015	0.76766	0.0003
C	71	0.1538	1.99875	3.82148	0.02598	5.8462	0.00329
C	72	–0.03915	1.99905	4.02076	0.01934	6.03915	0.00271
C	73	–0.20493	1.99914	4.18969	0.01611	6.20493	–0.00097
H	74	0.20882	0	0.78942	0.00176	0.79118	0.00003
C	75	–0.22373	1.99921	4.20757	0.01695	6.22373	0.00215
H	76	0.21203	0	0.78644	0.00153	0.78797	–0.00007
C	77	–0.21668	1.99914	4.20101	0.01654	6.21668	–0.00081
H	78	0.20788	0	0.79049	0.00163	0.79212	0.00002
C	79	–0.03232	1.99905	4.01413	0.01915	6.03232	0.00178
C	80	–0.61915	1.99944	4.60831	0.0114	6.61915	0.00057
H	81	0.2153	0	0.78266	0.00204	0.7847	0.00015
C	82	–0.62336	1.99943	4.61346	0.01046	6.62336	–0.00027
H	83	0.23141	0	0.76673	0.00186	0.76859	0.00088
C	84	0.12499	1.99866	3.84861	0.02774	5.87501	0.00634
C	85	–0.02366	1.99907	4.00393	0.02066	6.02366	0.00158
C	86	–0.21322	1.99913	4.19749	0.01659	6.21322	0.00002
H	87	0.20899	0	0.78947	0.00153	0.79101	0
C	88	–0.21772	1.99922	4.20152	0.01699	6.21772	0.00011
H	89	0.21325	0	0.78538	0.00137	0.78675	0

C	90	–0.20642	1.99914	4.19121	0.01607	6.20642	0.0002
H	91	0.20941	0	0.78896	0.00164	0.79059	0.00001
C	92	–0.03509	1.99906	4.01617	0.01986	6.03509	0.00147
C	93	–0.61972	1.99943	4.60949	0.0108	6.61972	0.0003
H	94	0.2309	0	0.76682	0.00228	0.7691	0.00219
C	95	–0.61956	1.99944	4.60942	0.01069	6.61956	0.00072
H	96	0.21775	0	0.78027	0.00198	0.78225	0.00012
C	97	–0.07212	1.99926	4.05638	0.01647	6.07212	0.00112
H	98	0.2278	0	0.76992	0.00228	0.7722	0.00031
H	99	0.18212	0	0.81569	0.00219	0.81788	0.00006
C	100	–0.43339	1.99946	4.42331	0.01062	6.43339	0.00005
H	101	0.2265	0	0.77206	0.00144	0.7735	–0.00005
H	102	0.21362	0	0.78481	0.00157	0.78638	0.00001
C	103	–0.43509	1.99946	4.42537	0.01026	6.43509	0.00012
H	104	0.22643	0	0.77218	0.00139	0.77357	–0.00009
H	105	0.20893	0	0.78942	0.00164	0.79107	–0.00003
C	106	–0.06871	1.99931	4.05203	0.01737	6.06871	0.00146
H	107	0.20213	0	0.79599	0.00188	0.79787	0.00006
H	108	0.19199	0	0.80589	0.00211	0.80801	0.00014
H	109	0.21919	0	0.77935	0.00146	0.78081	0.00009
H	110	0.22482	0	0.7725	0.00268	0.77518	0.00256
H	111	0.21958	0	0.77851	0.00191	0.78042	–0.00014
H	112	0.21494	0	0.78375	0.00131	0.78506	0.00002
H	113	0.21612	0	0.78257	0.00131	0.78388	0.00007
H	114	0.22551	0	0.77269	0.0018	0.77449	0.00005
H	115	0.22804	0	0.76927	0.00269	0.77196	0.00227
H	116	0.21789	0	0.78073	0.00139	0.78211	0.00009
H	117	0.21776	0	0.78081	0.00143	0.78224	0.00008
H	118	0.22535	0	0.77187	0.00278	0.77465	0.00308
H	119	0.21956	0	0.77849	0.00195	0.78044	0.00005
H	120	0.21375	0	0.78491	0.00134	0.78625	0.00006

H	121	0.21641	0	0.78231	0.00128	0.78359	0.00011
H	122	0.22623	0	0.77201	0.00176	0.77377	0.00005
H	123	0.22901	0	0.76855	0.00244	0.77099	0.00175
H	124	0.2181	0	0.78057	0.00133	0.7819	0.00005
C	125	–0.5386	1.99824	4.50304	0.03732	6.5386	–0.06323
H	126	0.25973	0	0.73846	0.00181	0.74027	0.0033
C	127	–0.53991	1.99824	4.50254	0.03913	6.53991	–0.05277
H	128	0.2719	0	0.72616	0.00194	0.7281	0.00328
C	129	–0.51719	1.99822	4.48267	0.0363	6.51719	–0.073
H	130	0.28342	0	0.7146	0.00198	0.71658	0.00382
C	131	–0.53274	1.99827	4.49557	0.0389	6.53274	–0.07569
H	132	0.25822	0	0.73979	0.00199	0.74178	0.00435
C	133	–0.52758	1.99826	4.49117	0.03815	6.52758	–0.06413
H	134	0.27025	0	0.72783	0.00192	0.72975	0.00359
C	135	–0.52189	1.99826	4.48499	0.03863	6.52189	–0.07828
H	136	0.27924	0	0.71883	0.00194	0.72076	0.00398
Total		0	231.85957	341.84428	2.29615	576	10

Average spin density on samarium: 5.15

Average charge on samarium: 1.22

Total charge of the bound benzene (C+H)/(C): –1.56/–3.18

Average charge on nitrogen donors of the BDI ligand: –0.65

Average charge on oxygen of THF: –0.53

Table. S12. Natural population analysis for **3-Sm (^{11}A)**.

Atom	No.	Natural Charge	Core	Valence	Rydberg	Total	Natural Spin Density
Sm	1	1.44316	53.95481	6.10745	0.49458	60.55684	5.09428
O	2	–0.64288	1.99964	6.6146	0.02864	8.64288	0.00644
N	3	–0.74173	1.99906	5.7088	0.03387	7.74173	0.00403
N	4	–0.74019	1.99906	5.70406	0.03706	7.74019	0.00607
C	5	–0.7165	1.99913	4.70685	0.01052	6.7165	–0.00059
H	6	0.24615	0	0.75214	0.00171	0.75385	0.00054
H	7	0.24213	0	0.75663	0.00124	0.75787	–0.00006
H	8	0.25445	0	0.74404	0.0015	0.74555	0.00053
C	9	0.32208	1.99854	3.65973	0.01965	5.67792	0.01233
C	10	–0.45096	1.99869	4.43443	0.01783	6.45096	–0.00641
H	11	0.23193	0	0.76647	0.00161	0.76807	0.00027
C	12	0.31863	1.99855	3.66327	0.01955	5.68137	0.01182
C	13	–0.71746	1.99913	4.70777	0.01057	6.71746	–0.0006
H	14	0.25396	0	0.74445	0.00159	0.74604	0.00055
H	15	0.24189	0	0.75686	0.00125	0.75811	–0.00006
H	16	0.24718	0	0.7512	0.00161	0.75282	0.0005
C	17	0.15229	1.99833	3.82622	0.02316	5.84771	0.00375
C	18	–0.04581	1.99869	4.02896	0.01816	6.04581	0.00166
C	19	–0.22884	1.9988	4.213	0.01704	6.22884	–0.00002
H	20	0.23613	0	0.76211	0.00176	0.76387	0
C	21	–0.23939	1.99887	4.22286	0.01766	6.23939	0.00013
H	22	0.23849	0	0.75992	0.0016	0.76151	0
C	23	–0.23334	1.99881	4.2177	0.01683	6.23334	–0.00012
H	24	0.23604	0	0.76224	0.00172	0.76396	0
C	25	–0.0559	1.99868	4.03885	0.01836	6.0559	0.00153
C	26	–0.66047	1.99917	4.64853	0.01277	6.66047	–0.00052
H	27	0.23499	0	0.76133	0.00368	0.76501	0.00241

C	28	–0.66611	1.99916	4.65466	0.01228	6.66611	0.00061
H	29	0.23871	0	0.75962	0.00168	0.76129	0.00008
C	30	0.1651	1.99836	3.81394	0.02261	5.8349	0.00256
C	31	–0.05047	1.99868	4.03412	0.01767	6.05047	0.00157
C	32	–0.23182	1.99881	4.21613	0.01687	6.23182	–0.00021
H	33	0.23459	0	0.76367	0.00174	0.76541	–0.00001
C	34	–0.24697	1.99887	4.23067	0.01743	6.24697	–0.00009
H	35	0.2368	0	0.76159	0.00161	0.7632	0.00001
C	36	–0.23818	1.99881	4.22211	0.01727	6.23818	–0.00016
H	37	0.23392	0	0.76433	0.00175	0.76608	0
C	38	–0.04467	1.9987	4.02825	0.01772	6.04467	0.00123
C	39	–0.66918	1.99916	4.65711	0.01291	6.66918	0.0008
H	40	0.23914	0	0.7591	0.00176	0.76086	0.00003
C	41	–0.66478	1.99917	4.65291	0.01269	6.66478	0.00036
H	42	0.2405	0	0.75608	0.00342	0.7595	0.00156
C	43	–0.06643	1.99884	4.0495	0.0181	6.06643	0.00094
H	44	0.23389	0	0.76311	0.003	0.76611	0.00014
H	45	0.21015	0	0.78653	0.00332	0.78985	0.00018
C	46	–0.49217	1.99906	4.47851	0.0146	6.49217	0.00004
H	47	0.25217	0	0.74614	0.00169	0.74783	–0.00014
H	48	0.23761	0	0.75984	0.00255	0.76239	0.00001
C	49	–0.4933	1.99906	4.47969	0.01455	6.4933	0.00007
H	50	0.25175	0	0.74659	0.00167	0.74825	–0.00001
H	51	0.2378	0	0.75978	0.00242	0.7622	0.00001
C	52	–0.06692	1.99889	4.04983	0.01819	6.06692	0.00108
H	53	0.23145	0	0.76522	0.00333	0.76855	0.00047
H	54	0.21344	0	0.78317	0.0034	0.78656	0.00016
Sm	55	1.32043	53.95364	6.1752	0.55072	60.67957	5.20124
O	56	–0.63342	1.99963	6.60898	0.02481	8.63342	0.00731
N	57	–0.7106	1.99902	5.66931	0.04227	7.7106	–0.00418
N	58	–0.7437	1.99901	5.70686	0.03783	7.7437	0.00362

C	59	–0.71634	1.99913	4.70687	0.01033	6.71634	–0.00077
H	60	0.24671	0	0.7517	0.00159	0.75329	0.00014
H	61	0.24282	0	0.75587	0.00132	0.75718	–0.00011
H	62	0.25342	0	0.74508	0.0015	0.74658	0.00028
C	63	0.33379	1.99856	3.64753	0.02013	5.66621	0.00723
C	64	–0.46036	1.9987	4.4437	0.01795	6.46036	–0.00057
H	65	0.23429	0	0.76416	0.00156	0.76571	0.00016
C	66	0.31886	1.99854	3.66264	0.01996	5.68114	0.00355
C	67	–0.71388	1.99912	4.70425	0.01051	6.71388	–0.00057
H	68	0.25251	0	0.74596	0.00153	0.74749	0.00013
H	69	0.24156	0	0.75718	0.00126	0.75844	–0.00005
H	70	0.24766	0	0.75084	0.0015	0.75234	0.00012
C	71	0.17102	1.99834	3.80714	0.02349	5.82898	0.00389
C	72	–0.03971	1.99869	4.02338	0.01764	6.03971	0.00234
C	73	–0.23995	1.99882	4.22388	0.01725	6.23995	–0.00069
H	74	0.23303	0	0.76519	0.00178	0.76697	0.00001
C	75	–0.2525	1.99888	4.23576	0.01786	6.2525	0.0012
H	76	0.23453	0	0.76378	0.00169	0.76547	–0.00004
C	77	–0.23389	1.99881	4.21862	0.01646	6.23389	–0.00008
H	78	0.23482	0	0.76347	0.00171	0.76518	0
C	79	–0.0485	1.99867	4.03139	0.01844	6.0485	0.00143
C	80	–0.66787	1.99916	4.65654	0.01216	6.66787	0.00043
H	81	0.24232	0	0.75454	0.00314	0.75768	0.0014
C	82	–0.66639	1.99917	4.6542	0.01303	6.66639	0.00068
H	83	0.2407	0	0.75771	0.00159	0.7593	0.00007
C	84	0.16113	1.99831	3.81813	0.02243	5.83887	0.00436
C	85	–0.05755	1.99861	4.03939	0.01955	6.05755	0.00149
C	86	–0.23015	1.9988	4.21411	0.01724	6.23015	0.0007
H	87	0.23424	0	0.76411	0.00165	0.76576	–0.00002
C	88	–0.24249	1.99887	4.22609	0.01753	6.24249	0.00024
H	89	0.23747	0	0.76094	0.00159	0.76253	–0.00001

C	90	–0.23004	1.99882	4.21424	0.01699	6.23004	0.00014
H	91	0.23566	0	0.76257	0.00177	0.76434	–0.00002
C	92	–0.04597	1.99869	4.03016	0.01712	6.04597	0.00052
C	93	–0.65834	1.99909	4.64467	0.01457	6.65834	–0.00078
H	94	0.24392	0	0.75439	0.00169	0.75608	0.00021
C	95	–0.67503	1.99916	4.66455	0.01132	6.67503	0.00005
H	96	0.2467	0	0.75096	0.00234	0.7533	0.00035
C	97	–0.07169	1.99881	4.05455	0.01833	6.07169	0.00119
H	98	0.24192	0	0.75484	0.00324	0.75808	0.00002
H	99	0.20684	0	0.79004	0.00312	0.79316	0.00019
C	100	–0.48878	1.99906	4.47507	0.01465	6.48878	0.00006
H	101	0.25371	0	0.7446	0.00169	0.74629	–0.00003
H	102	0.23794	0	0.75985	0.00221	0.76206	0.00003
C	103	–0.49516	1.99906	4.48167	0.01443	6.49516	0.00012
H	104	0.25398	0	0.74448	0.00154	0.74602	–0.00001
H	105	0.23958	0	0.75793	0.00249	0.76042	0
C	106	–0.07283	1.99889	4.05537	0.01857	6.07283	0.00205
H	107	0.22931	0	0.76785	0.00284	0.77069	0.00001
H	108	0.21024	0	0.7862	0.00356	0.78976	0.00029
H	109	0.24688	0	0.75133	0.0018	0.75312	0.00005
H	110	0.24004	0	0.75865	0.00131	0.75996	0.00008
H	111	0.23957	0	0.75902	0.00141	0.76043	0.00002
H	112	0.24158	0	0.75539	0.00303	0.75842	0.00146
H	113	0.23942	0	0.75914	0.00145	0.76058	0.00002
H	114	0.24058	0	0.75589	0.00353	0.75942	0.00183
H	115	0.24695	0	0.75132	0.00174	0.75305	0.00002
H	116	0.23774	0	0.7609	0.00136	0.76226	0.00005
H	117	0.24389	0	0.75439	0.00172	0.75611	0.00014
H	118	0.23803	0	0.76061	0.00136	0.76197	0.00004
H	119	0.24105	0	0.75747	0.00148	0.75895	0.00002
H	120	0.23943	0	0.75745	0.00312	0.76057	0.00087

H	121	0.24739	0	0.75122	0.00139	0.75261	–0.00017
H	122	0.22809	0	0.76605	0.00586	0.77191	0.00358
H	123	0.24071	0	0.75722	0.00207	0.75929	0.00005
H	124	0.24025	0	0.75827	0.00148	0.75975	–0.00005
C	125	–0.54578	1.99827	4.50125	0.04625	6.54578	–0.08266
H	126	0.26783	0	0.72915	0.00302	0.73217	0.00376
C	127	–0.33276	1.99813	4.28626	0.04837	6.33276	–0.12074
C	128	–0.60165	1.99824	4.55203	0.05138	6.60165	–0.00459
H	129	0.26586	0	0.73023	0.00391	0.73414	0.00237
C	130	–0.57457	1.99941	4.56026	0.0149	6.57457	0.00938
H	131	0.22145	0	0.77392	0.00463	0.77855	–0.00862
H	132	0.23068	0	0.76697	0.00235	0.76932	–0.00167
H	133	0.21096	0	0.78703	0.00201	0.78904	–0.00333
C	134	–0.54178	1.99827	4.49737	0.04614	6.54178	–0.05727
H	135	0.27249	0	0.7236	0.00391	0.72751	0.00297
C	136	–0.31204	1.99822	4.25891	0.0549	6.31204	–0.12926
C	137	–0.58373	1.99832	4.53602	0.04939	6.58373	–0.00318
H	138	0.2638	0	0.73291	0.00329	0.7362	0.00193
C	139	–0.57566	1.9994	4.56031	0.01595	6.57566	0.01165
H	140	0.21797	0	0.77991	0.00212	0.78203	–0.00479
H	141	0.23157	0	0.76589	0.00254	0.76843	–0.00184
H	142	0.19577	0	0.80073	0.0035	0.80423	–0.01121
Total		0	235.83384	353.58819	2.57797	592	10

Average spin density on samarium: 5.15

Average charge on samarium: 1.38

Total charge of the bound *p*-xylene excluding two methyl groups (C+H)/(C): –1.69/–2.92

Average charge on nitrogen donors of the BDI ligand: –0.73

Average charge on oxygen of THF: –0.64

Table. S13. Calculated natural charges for **2-M** and **3-M** (M = Y and Sm).

Cpd.	avg. M	Total (C+H)/(C) of the bound ring (excluding Me)	avg. N	avg. O	avg. Spin density of M
**2-Y**	1.57	–1.87/–3.49	–0.67	/	/
**3-Y**	1.69	–2.02/–3.19	–0.69	/	/
**2-Sm (^{11}A)**	1.22	–1.56/–3.18	–0.65	–0.53	5.15
**3-Sm (^{11}A)**	1.38	–1.69/–2.92	–0.73	–0.64	5.15

Table. S14. Wiberg bond index for **2-M** and **3-M** (M = Y and Sm).

Complexes	Avg. M–N	Avg. M–O	Avg. M–C	Avg. C–C for the bound arene
**2-Y**	0.23	/	0.25	1.12
**3-Y**	0.20	/	0.22	1.12
**2-Sm (^{11}A)**	0.22	0.16	0.27	1.12
**3-Sm (^{11}A)**	0.19	0.14	0.24	1.11

S7. References

1. T. X. Gentner, B. Rösch, G. Ballmann, J. Langer, H. Elsen and S. Harder, *Angew. Chem. Int. Ed.*, 2019, **58**, 607-611.
2. B. Rösch, T. X. Gentner, J. Langer, C. Färber, J. Eyselein, L. Zhao, C. Ding, G. Frenking and S. Harder, *Science*, 2021, **371**, 1125-1128.
3. D. E. Bergbreiter and J. M. Killough, *J. Am. Chem. Soc.*, 1978, **100**, 2126-2134.
4. K. Izod, S. T. Liddle and W. Clegg, *Inorg. Chem.*, 2004, **43**, 214-218.
5. G. Sheldrick, *Acta Crystallogr. Sect. A*, 2015, **71**, 3-8.
6. G. Sheldrick, *Acta Crystallogr. Sect. A*, 2008, **64**, 112-122.
7. G. Sheldrick, *Acta Crystallogr. Sect. C-Struct. Chem.*, 2015, **71**, 3-8.
8. O. V. Dolomanov, L. J. Bourhis, R. J. Gildea, J. A. K. Howard and H. Puschmann, *J. Appl. Crystallogr.*, 2009, **42**, 339-341.
9. A. Spek, *J. Appl. Crystallogr.*, 2003, **36**, 7-13.
10. A. Spek, *Acta Crystallogr. Sect. C-Struct. Chem.*, 2015, **71**, 9-18.
11. F. Neese, *WIREs Comput. Mol. Sci.*, 2012, **2**, 73-78.
12. C. Adamo and V. Barone, *J. Chem. Phys.*, 1999, **110**, 6158-6170.
13. S. Grimme, J. Antony, S. Ehrlich and H. Krieg, *J. Chem. Phys.*, 2010, **132**, 154104.
14. S. Grimme, S. Ehrlich and L. Goerigk, *J. Comput. Chem.*, 2011, **32**, 1456-1465.
15. E. v. Lenthe, E. J. Baerends and J. G. Snijders, *J. Chem. Phys.*, 1993, **99**, 4597-4610.
16. D. A. Pantazis and F. Neese, *J. Chem. Theory Comput.*, 2009, **5**, 2229-2238.
17. J. D. Rolfes, F. Neese and D. A. Pantazis, *J. Comput. Chem.*, 2020, **41**, 1842-1849.
18. F. Weigend and R. Ahlrichs, *Phys. Chem. Chem. Phys.*, 2005, **7**, 3297-3305.
19. E. D. Glendening, C. R. Landis and F. Weinhold, *J. Comput. Chem.*, 2013, **34**, 1429-1437.
20. W. Humphrey, A. Dalke and K. Schulten, *J. Mol. Graph.*, 1996, **14**, 33-38.
21. T. Lu and F. Chen, *J. Comput. Chem.*, 2012, **33**, 580-592.
